# How is overall survival assessed in randomised clinical trials in cancer and are subsequent treatment lines considered? A systematic review

**DOI:** 10.1186/s13063-023-07730-1

**Published:** 2023-11-06

**Authors:** Kara-Louise Royle, David Meads, Jennifer K. Visser-Rogers, Ian R. White, David A. Cairns

**Affiliations:** 1 Leeds Cancer Research UK Clinical Trials Unit, Leeds Institute of Clinical Trials Research, University of Leeds, Leeds, UK; 2 Academic Unit of Health Economics, Leeds Institute of Health Sciences, University of Leeds, Leeds, UK; 3 PHASTAR, London, UK; 4 MRC Clinical Trials Unit at UCL, London, UK

**Keywords:** Systematic review, Overall survival, Randomised controlled trial, Cancer, Subsequent treatment lines

## Abstract

**Background:**

Overall survival is the “gold standard” endpoint in cancer clinical trials. It plays a key role in determining the clinical- and cost-effectiveness of a new intervention and whether it is recommended for use in standard of care. The assessment of overall survival usually requires trial participants to be followed up for a long period of time. In this time, they may stop receiving the trial intervention and receive subsequent anti-cancer treatments, which also aim to extend survival, during trial follow-up. This can potentially change the interpretation of overall survival in the context of the clinical trial. This review aimed to determine how overall survival has been assessed in cancer clinical trials and whether subsequent anti-cancer treatments are considered.

**Methods:**

Two searches were conducted using MEDLINE within OVID© on the 9th of November 2021. The first sought to identify papers publishing overall survival results from randomised controlled trials in eight reputable journals and the second to identify papers mentioning or considering subsequent treatments. Papers published since 2010 were included if presenting or discussing overall survival in the context of treating cancer.

**Results:**

One hundred and thirty-four papers were included. The majority of these were presenting clinical trial results (98, 73%). Of these, 45 (46%) reported overall survival as a (co-) primary endpoint. A lower proportion of papers including overall survival as a (co-) primary endpoint compared to a secondary endpoint were published in recent years. The primary analysis of overall survival varied across the papers. Fifty-nine (60%) mentioned subsequent treatments. Seven papers performed additional analysis, primarily when patients in the control arm received the experimental treatment during trial follow-up (treatment switching).

**Discussion:**

Overall survival has steadily moved from being the primary to a secondary endpoint. However, it is still of interest with papers presenting overall survival results with the caveat of subsequent treatments, but little or no investigation into their effect. This review shows that there is a methodological gap for what researchers should do when trial participants receive anti-cancer treatment during trial follow-up. Future research will identify the stakeholder opinions, on how this methodological gap should be addressed.

**Supplementary Information:**

The online version contains supplementary material available at 10.1186/s13063-023-07730-1.

## Introduction

Overall survival (OS), disease-free survival, progression-free survival and cause-specific survival are all used to assess the clinical effectiveness of experimental treatments in cancer clinical trials. However, due to subjectivity and variability in the definitions of response, progression, and cause of death. OS is the only endpoint that has a definite event which cannot be questioned. As a result of this, OS is often referred to as the “gold standard” endpoint for assessing the clinical effectiveness of experimental interventions for the treatment of cancer. In England, OS is used in conjunction with other evidence in appraisals by NICE (National Institute for Health and Care Excellence) to determine whether interventions should be recommended for use in the NHS. A recent review of Health Technology Assessment (HTA) systems in the European Union, UK, Canada and Australia, found that the model in England which uses clinical and cost-effectiveness data in their review alongside other evidence is similar to eight other HTA bodies including France and Sweden. In addition, there were 46 other HTA bodies across multiple countries including Italy, The Netherlands and Spain which also use both clinical and cost-effectiveness data in their appraisals [1].

To ensure consistency between clinical trials, guidance exists on their analysis and interpretation. The International Council on Harmonisation of Technical Requirements for Registration of Pharmaceuticals for Human Use (ICH) topic E9 is entitled “Statistical Principles for Clinical Trials” and includes, for example, advice on how to analyse a trial when stratification factors are used during randomisation [2]. In addition, for time-to-event endpoints like OS, a stakeholder consultation on presenting results using Kaplan-Meier plots was published in 2019 [3]. Finally, OS itself has the published definitive definition as time from randomisation to death from any cause [4].

The assessment of OS requires trial participants to be followed up for what can be a considerable length of time. This poses a problem when it comes to interpreting the trial results, as participants may discontinue their trial intervention prior to death. For example, participants may discontinue at disease progression or earlier due to withdrawal from trial treatment (as a result of toxicity or patient choice), or an intervention stopping rule. Trial participants may then go on to receive multiple subsequent lines of anti-cancer treatment during trial follow-up. These subsequent treatments will also aim to control a patient’s cancer and therefore may improve OS. This improvement may reduce the number of OS events observed in the trial and in turn reduce the power available to detect a difference in OS between the experimental and control interventions. Consequentially, using England as an example, this could lead to a beneficial intervention not being recommended for use in the NHS, resulting in sub-optimal patient outcomes.

This issue with interpretation is well known and is referenced in clinical trial recommendations [4]. Methodological solutions exist to account for scenarios where participants in the control arm switch to receive the experimental intervention, by design or in a later line of therapy (treatment switching). These methods were summarised as part of a technical support document in 2014 and include the rank preserving structural failure time model (RPSFTM) and the Inverse Probability Censoring Weights (IPCW) method [5].

It is currently unknown how widely the published guidance on the analysis of OS is followed and how researchers at present perceive subsequent treatment lines as a whole compared to the specific scenario of treatment switching. Therefore, the aim of this systematic review was to determine how OS is currently analysed in practice and whether subsequent lines of treatment are mentioned in the main manuscript or supplementary material of cancer clinical trials publications.

## Methods

### Search strategy

The electronic database MEDLINE using OVID® was searched on 9 November 2021. To ensure that the search was not biased towards trials that did or did not report the limitations surrounding the analysis of OS in terms of subsequent treatments, two approaches were taken (Table 1). The first approach aimed to gain a broad view of the analysis of OS in cancer clinical trials. Due to the breadth of publications available, the search was restricted to several major journals focusing on general medicine and cancer: *Lancet*, *Lancet Oncology*, *JCO*, *PLOS One*, *PLOS Medicine*, *BMJ*, *JAMA* and *New England Journal of Medicine*. The second approach aimed to target papers which were specifically discussing the issue around subsequent treatments in conjunction with the analysis of OS in cancer clinical trials. Both searches were restricted to the English language and to papers published since 1 January 2010. The results of the two searches were combined with the Boolean operator OR and all abstracts were extracted into Microsoft Excel (Microsoft, Redmond, WA, USA) and Endnote^TM^ (Clarivate, Philadelphia, PA, USA).

**Table 1:** Literature search terms

**Approach 1 — Overall Survival**				
**Concept**	"Overall Survival".tw.	Randomi?ed Controlled Trial.tw.	Cancer.tw.	
**Synonyms**	OS mortality	RCT	Carcinoma Neoplasm Sarcoma	
**MESH**	"Survival Analysis"	Randomized Controlled Trials as Topic	Neoplasms	
**Approach 2 — Subsequent Treatment Lines**				
**Concept**	"Overall Survival".tw.	Randomi?ed Controlled Trial.tw.	"Subsequent Treatments"	Cancer.tw.
**Synonyms**	OS mortality	RCT	(Line* adj3 therap*) (Line* adj3 treatment*) (Subsequent adj3 therap*) (Subsequent adj3 treatment*) (Subsequent adj3 medication*)	Carcinoma Neoplasm Sarcoma
**MESH**	"Survival Analysis"	Randomized Controlled Trials as Topic		Neoplasms

Overview of the literature search. Each row for each approach (concept, synonyms, MESH term) was combined with the Boolean operator OR and each column was combined with the Boolean operator AND. Terms which were required to be in the title or abstract were suffixed with .tw., full phrases were specified with quotation marks, terms which could have multiple endings were suffixed with a * and terms which could have English or American spellings were noted with a? So both were included. Any terms where phrases could be given in several ways were specified using the adj3 term which looks for the terms within 3 words of each other. MESH subjects were considered as focussed terms only

*OS* Overall survival, *RCT* randomised controlled trial

### Initial screening process

**Fig. 1:**
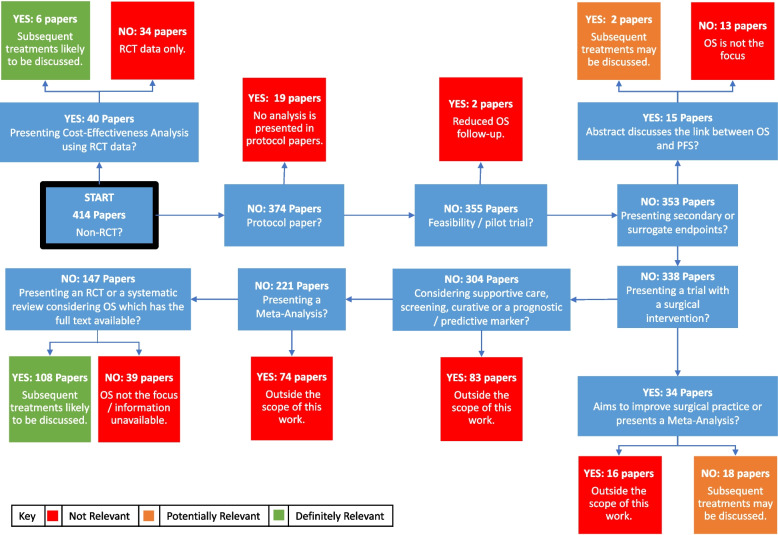
Screening process for abstracts. Flow diagram showing the process for abstracts to be included in the review

The results were screened based on their abstracts. The screening process was conducted by K-LR, reviewed by DAC and any uncertainty discussed. The screening process categorised the papers into red (not relevant), amber (potentially relevant), and green (relevant) (Fig. 1). Any paper which did not mention OS in the abstract was classed as red. In addition, if the abstract or full publication was not available it was also classed as red.

### Reading process

All amber and green papers which remained were read in full by K-LR. and categorised as presenting clinical effectiveness results of a clinical trial, presenting a cost-effectiveness analysis, or a methodology or review paper discussing multiple treatment lines. Category-specific information was extracted from each publication (Table 2) and summarised using descriptive statistics. Note that for the review, crossover by design was included in the definition a subsequent treatment line. Any uncertainty in the categorisation or interpretation of the information being extracted was discussed and agreed with DAC. The papers presenting cost-effectiveness were also read by DM given his experience as a Health Economist performing and reviewing cost-effective analysis and any disagreements discussed. The Kappa coefficient of agreement was also calculated. The PRISMA (Preferred Reporting Items for Systematic Reviews and Meta-Analyses) standards were followed for reporting (Additional file 1, Supplementary Tables 1 and 2) [6].

**Table 2:** Information extracted from each article type

**Article type**	**Information extracted**
Clinical effectiveness paper	*Overall survival (OS)* • Endpoint level (primary, secondary, exploratory, unclear) • Definition • The analysis used for the interpretation of the primary endpoint • Whether a Kaplan-Meier Plot for OS was included ○ Whether an at-risk table was included with the plot ○ Whether the at-risk table included the number censored/number of events ○ Whether confidence intervals were included on the plot • Whether a logrank test for OS was conducted ○ Whether the test was adjusted or unadjusted for other factors. • Whether a Cox proportional hazards model for OS was included ○ Whether the results were adjusted or unadjusted for other factors. *Subsequent treatment*^a^ • Whether the number or percent of trial participants receiving treatment in follow-up is included in the results or appendix. • Whether a summary of the number of treatment lines and/or types of treatments trial participants received. • Whether subsequent treatments are discussed in the discussion. ○ If yes — what are their thoughts? • Whether they did some form of analysis to account for subsequent treatments. ○ If yes — what analysis?
Cost-effectiveness paper	*Cost-effectiveness* • What OS results fed into the cost-effectiveness analysis and how were they determined. *Subsequent treatment* • Whether subsequent treatment costs were incorporated into the cost-effectiveness analysis • Whether subsequent treatments are discussed in the discussion. ○ If yes — what are their thoughts? • Whether they did some form of analysis to account for subsequent treatments. ○ If yes — what analysis?
Methodology/review paper	*General information* • Treatment overview or methodology paper? *Subsequent treatment* • Whether subsequent treatments are discussed. ○ If yes — what are their thoughts?

^a^Note that subsequent treatments included cross-over by design

## Results

### Overview and sample size

A total of 415 results were identified from the literature search. Following the screening process 281 (68%) were classed as red, 26 (6%) amber and 108 (26%) green (Fig. 1). No changes were made to the screening process following the DAC review. Therefore, a total of 134 papers were included in the review. Following the reading process these were identified to be 98 (73%) clinical effectiveness papers, 27 (20%) methodology or review papers and 9 (7%) cost-effectiveness papers.

### Clinical effectiveness papers

#### Overall survival

The 98 categorised clinical effectiveness papers along with citations are listed in Additional file 1, Supplementary Table 3. In total 45 (46%) of the clinical effectiveness papers reported OS to be the primary endpoint of the trial. Forty-eight papers (49%) reported OS as a secondary endpoint. Five papers (5%) stated that OS was an exploratory endpoint only.

OS was defined to be time from randomisation to death from any cause in 87 (89%) papers. Two papers (2%) defined the starting point to be diagnosis, three papers (3%) intervention initiation, one paper enrolment (1%) and one paper pre-inclusion (1%), but it was unclear what these meant in relation to the point of randomisation. Four papers (4%) did not explicitly define OS within the text, two of which were not presenting OS results.

**Fig. 2:**
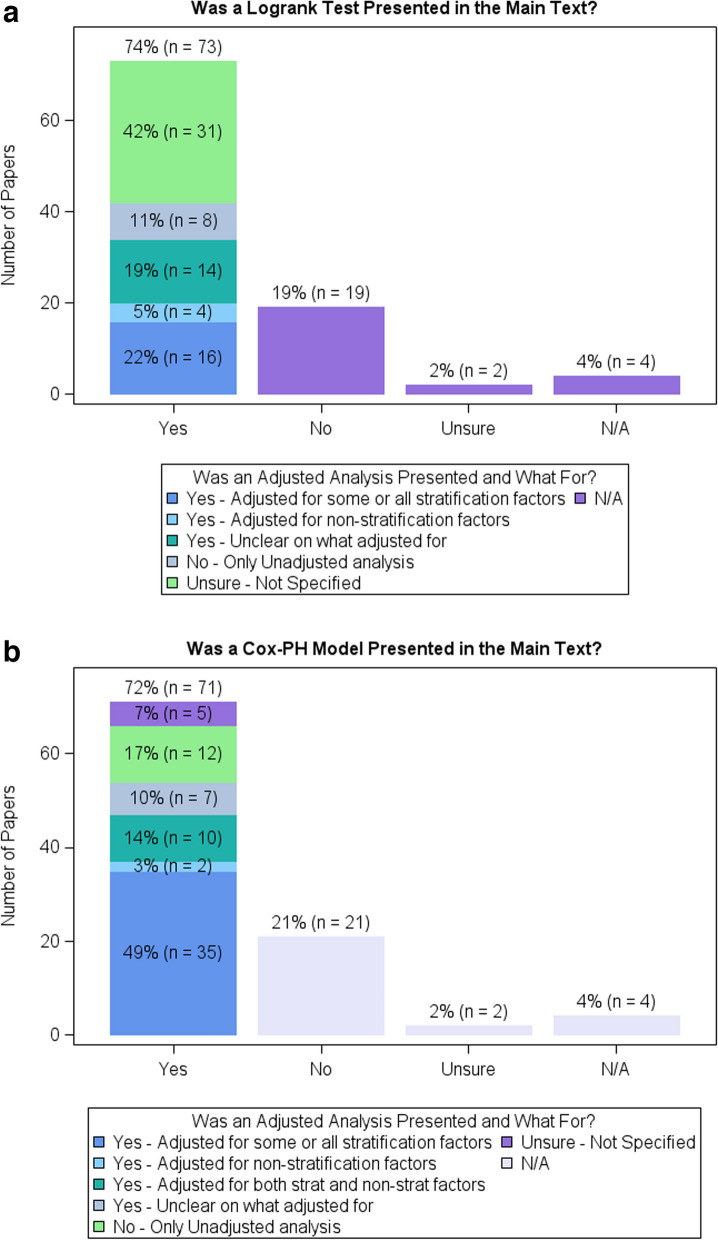
**a** Was a logrank test presented in the main text? Stacked bar chart showing whether a logrank test was presented in the main text of the effectiveness papers (quantified by the percentages at the top of the bar) and whether it was adjusted or unadjusted for additional factors (quantified by the percentages within the bar). **b** Was a Cox-PH model presented in the main text? Stacked bar chart showing whether a Cox-proportional hazards model was presented in the main text of the effectiveness papers (quantified by the percentages at the top of the bar) and whether it was adjusted or unadjusted for additional factors (quantified by the percentages within the bar)

Most papers (86/98, 88%) presented a Kaplan-Meier curve for OS in the main manuscript. Of these, the majority included the numbers at risk (79/86, 92%). However, only a small proportion included the number of events or number censored in their at-risk table (13/79, 16%). None of the papers provided confidence intervals on their Kaplan-Meier curve. Eight papers, with mature OS data, did not present a Kaplan-Meier curve for OS in their main manuscript. Four papers included them in the appendix [7–10] and four omitted a Kaplan-Meier curve entirely [11–14]. A similar proportion of papers presented a logrank test (73/98, 74%) and/or a Cox proportional hazards model (71/98, 72%). However, the level of adjustment (also referred to as stratification) of the tests varied across papers (Fig. 2). Of the 73 papers which included a logrank test, 31 (42%) were not clear as to whether they reported adjusted or unadjusted results (Fig. 2a). In contrast, around half of the papers which presented results of the Cox model reported results which clearly had been adjusted for some or all of the trial’s stratification factors (Fig. 2b, 35/71, 49%).

A summary of the primary analysis methods used for OS is shown by endpoint level in Table 3, by year in Additional file 1, Supplementary Table 4 and by cancer type in Additional file 1, Supplementary Table 5. The most popular primary analysis method was the combination of a *p*-value from a logrank test and the hazard ratio from a Cox proportional hazards model. In total 36 of the 98 papers (37%) papers interpreted OS results using the *p*-value from a stratified or unstratified logrank test and the hazard ratio from an adjusted or unadjusted Cox model. Of the 98 papers, 15 (15%) used a hazard ratio and *p*-value from an adjusted Cox model to interpret OS. Of these 15, 12 (80%), were when OS was a primary or co-primary endpoint. When considered by year (Additional file 1, Supplementary Table 4) of publication the proportion of papers reporting OS as a (co-)primary endpoint fluctuates over the years. Since 2018 OS was more often a secondary rather than (co-)primary endpoint (Additional file 1, Supplementary Table 4). Considering cancer type (Additional file 1, Supplementary Table 5), upper gastrointestinal/hepato-biliary cancers had the highest proportion of papers presenting OS as a primary endpoint.

**Table 3:** Summary of the clinical effectiveness papers by endpoint type

	**Primary or co-primary (*n*=45)**	**Secondary (*n*=48)**	**Exploratory (*n*=5)**	**Total (*n*=98)**
**Was a Kaplan-Meier curve for OS presented in the main text?**				
Yes	40 (88.9%)	41 (85.4%)	5 (100.0%)	86 (87.8%)
No	2 (4.4%)	6 (12.5%)	0 (0.0%)	8 (8.2%)
N/A^a^	3 (6.7%)	1 (2.1%)	0 (0.0%)	4 (4.1%)
**Were the numbers at risk included in the Kaplan-Meier curve?**				
Yes	36 (80.0%)	38 (79.2%)	5 (100.0%)	79 (80.6%)
No	4 (8.9%)	3 (6.3%)	0 (0.0%)	7 (7.1%)
N/A	5 (11.1%)	7 (14.6%)	0 (0.0%)	12 (12.2%)
**Were the number of patients censored or number of events included in the at risk table**				
Yes	9 (20.0%)	3 (6.3%)	1 (20.0%)	13 (13.3%)
No	27 (60.0%)	35 (72.9%)	4 (80.0%)	66 (67.3%)
N/A	9 (20.0%)	10 (20.8%)	0 (0.0%)	19 (19.4%)
**Were confidence intervals included in the Kaplan-Meier curve?**				
No	40 (88.9%)	41 (85.4%)	5 (100.0%)	86 (87.8%)
N/A	5 (11.1%)	7 (14.6%)	0 (0.0%)	12 (12.2%)
**Primary analysis**				
Hazard ratio and 95% CI from an unadjusted Cox model	1 (2.2%)	0 (0.0%)	0 (0.0%)	1 (1.0%)
Hazard ratio and 95% CI from an adjusted Cox model	1 (2.2%)	1 (2.1%)	0 (0.0%)	2 (2.0%)
Hazard ratio and *P*-value from a logrank test	8 (17.8%)	11 (22.9%)	0 (0.0%)	19 (19.4%)
Hazard ratio and *P*-value from a stratified logrank test.	3 (6.7%)	3 (6.3%)	0 (0.0%)	6 (6.1%)
Hazard ratio and *p*-value from an adjusted Cox model	12 (26.7%)	2 (4.2%)	1 (20.0%)	15 (15.3%)
Hazard ratio and *p*-value from an unadjusted Cox model	2 (4.4%)	3 (6.3%)	0 (0.0%)	5 (5.1%)
Hazard ratio from a Cox model and *p*-value from a logrank test	3 (6.7%)	7 (14.6%)	0 (0.0%)	10 (10.2%)
Hazard ratio from an adjusted Cox model and *p*-value from a logrank test.	2 (4.4%)	4 (8.3%)	0 (0.0%)	6 (6.1%)
Hazard ratio from an adjusted Cox model and *p*-value from a stratified logrank test.	5 (11.1%)	5 (10.4%)	3 (60.0%)	13 (13.3%)
Hazard ratio from an unadjusted Cox model and *p*-value from a logrank test.	1 (2.2%)	4 (8.3%)	0 (0.0%)	5 (5.1%)
Hazard ratio from an unadjusted Cox model and *p*-value from a stratified logrank test.	0 (0.0%)	2 (4.2%)	0 (0.0%)	2 (2.0%)
N/A^a^	3 (6.7%)	1 (2.1%)	0 (0.0%)	4 (4.1%)
Other	4 (8.9%)	5 (10.4%)	1 (20.0%)	10 (10.2%)
**Were subsequent treatments mentioned in the paper?**				
Yes	33 (73.3%)	23 (47.9%)	3 (60.0%)	59 (60.2%)
No	12 (26.7%)	25 (52.1%)	2 (40.0%)	39 (39.8%)
**Was the percentage or number of participants who received later lines included in the main text or appendix?**				
Yes	31 (68.9%)	18 (37.5%)	1 (20.0%)	50 (51.0%)
No	1 (2.2%)	5 (10.4%)	2 (40.0%)	8 (8.2%)
N/A	13 (28.9%)	25 (52.1%)	2 (40.0%)	40 (40.8%)
**Was a breakdown or summary of the number of subsequent treatments (two, three, four) included in the main text or appendix?**				
Yes	3 (6.7%)	1 (2.1%)	0 (0.0%)	4 (4.1%)
No	28 (62.2%)	22 (45.8%)	3 (60.0%)	53 (54.1%)
N/A^b^	14 (31.1%)	25 (52.1%)	2 (40.0%)	41 (41.8%)
**Was a breakdown or summary of the type of subsequent treatments (treatment 1, treatment 2, treatment 3) included in the main text or appendix?**				
Yes	26 (57.8%)	18 (37.5%)	1 (20.0%)	45 (45.9%)
No	5 (11.1%)	5 (10.4%)	2 (40.0%)	12 (12.2%)
N/A^b^	14 (31.1%)	25 (52.1%)	2 (40.0%)	41 (41.8%)
**Was additional analysis conducted to account for subsequent treatment lines?**				
Yes	2 (4.4%)	3 (6.3%)	2 (40.0%)	7 (7.1%)
No	30 (66.7%)	20 (41.7%)	1 (20.0%)	51 (52.0%)
N/A^b^	14 (31.1%)	25 (52.1%)	2 (40.0%)	41 (41.8%)
**Were subsequent treatment lines mentioned in the discussion?**				
Yes	22 (48.9%)	15 (31.3%)	2 (40.0%)	39 (39.8%)
No	11 (24.4%)	8 (16.7%)	1 (20.0%)	20 (20.4%)
N/A	12 (26.7%)	25 (52.1%)	2 (40.0%)	39 (39.8%)
**How were subsequent treatments mentioned in the discussion?**				
The uptake of subsequent treatments is given as a reason for the discrepancy between OS and surrogate endpoints.	0 (0.0%)	2 (4.2%)	0 (0.0%)	2 (2.0%)
The uptake of subsequent treatments is given as a reason for better OS results or a reduced event rate than expected.	4 (8.9%)	1 (2.1%)	1 (20.0%)	6 (6.1%)
The uptake of subsequent treatments is given as a reason for reduced OS effect/stated as may have affected the results/used to caveat the results.	6 (13.3%)	7 (14.6%)	1 (20.0%)	14 (14.3%)
The lack of uptake of subsequent treatments is given as a reason for no OS effect/may have negatively affected OS.	4 (8.9%)	0 (0.0%)	0 (0.0%)	4 (4.1%)
Subsequent treatments are stated to not have affected the OS results as an OS benefit was observed or OS was similar between those who did and did not receive a subsequent treatment.	2 (4.4%)	1 (2.1%)	0 (0.0%)	3 (3.1%)
Randomisation/balance of subsequent treatment lines is given as a reason as to why subsequent treatment lines will not have affected the OS results.	2 (4.4%)	2 (4.2%)	0 (0.0%)	4 (4.1%)
None or limited options of subsequent treatment lines for patients are given as a reason as to why subsequent treatment lines will not have affected the OS results.	0 (0.0%)	2 (4.2%)	0 (0.0%)	2 (2.0%)
Other	4 (8.9%)	0 (0.0%)	0 (0.0%)	4 (4.1%)
N/A	23 (51.1%)	33 (68.8%)	3 (60.0%)	59 (60.2%)

*N/A* Not applicable, *OS* Overall survival

^a^Not applicable here refers to papers where OS is a pre-specified endpoint, but the data is not mature at the time of the publication [15–18]

^b^Note one paper stated no patients received subsequent treatments so is classed as N/A for these summaries

#### Subsequent treatment lines

In total, 59 of the 98 (60%) papers mentioned subsequent treatment lines in the main manuscript or supplementary material. Of those that mentioned subsequent treatment lines, the majority (50/59, 85%,) provided the number or percentage of participants who received a later treatment line during trial follow-up. Four of the 59 (7%) papers provided a breakdown of the number of later lines a participant received. However, 45 of the 59 (76%) provided a summary of the types of treatments participants received during follow-up. Finally, seven of the 59 (12%) papers performed additional analysis addressing subsequent treatments. The most common reason for the additional analysis was due to participants switching to receive the experimental intervention [10, 19–21]. Miles et al. [19] included time-dependent covariates in the Cox proportional hazards model to adjust for trial participants crossing over from placebo to active treatment. Dimopoulos et al. [22] conducted a post hoc landmark analysis which measured OS from time of progression. Hodi et al. [23] included a sensitivity analysis for OS which censored participants at the point of subsequent treatment. Middleton et al. [20] conducted a post hoc landmark analysis which compared the survival of patients on the active arm who received standard chemotherapy after progression with those who were still alive on the standard chemotherapy arm at this timepoint. Goldhirsch et al. [10] conducted an analysis which cut off follow-up at specific timepoints to report how the effect changed over time after patients switched from observation to active in follow-up. Penichoux et al. [12] included time-dependent covariates in various Cox proportional hazards models to account for progression, toxicity and introduction of third-line treatment, they also modelled progression and toxicity events as repeating events using shared gamma frailty models where death was classed as the definitive event. Finally, Gianni et al. [21] conducted an analysis which censored patients at the point of cross-over from observation to active treatment. In the discussion section, 39 of the 59 (66%) papers mentioned subsequent treatment lines (Table 3). The most common discussion point for 14 of the 39 (36%) papers was “The uptake of subsequent treatments is given as a reason for reduced OS effect / stated as may have affected the results / used to caveat the results*.*” However, four papers also stated that “The lack of uptake of subsequent treatments is given as a reason for no OS effect / may have negatively affected OS.”

### Cost-effectiveness papers

In total nine papers whose primary purpose was to report cost-effectiveness analysis were identified during the literature review (Additional file 1, Supplementary Table 6). There were no disagreements between K-LR and DM following review. Therefore, the Kappa coefficient was one for each summary.

The majority of the papers (6/9, 67%) used a partitioned survival analysis model where the OS estimate was extrapolated from a Kaplan-Meier plot (5/9, 56%). Five of the nine (56%) papers included subsequent costs and four of the nine (44%) papers performed additional analysis to account for subsequent lines of therapy. One paper used the rank preserving structural failure time model (RPSFTM) [24], another used the two-stage method [25] and the other two adjusted for the cross-over in the trial, but the method used was not specified [26, 27].

### Methodology papers

In total, 27 methodology/review papers were identified (Additional file 1, Supplementary Table 7). Of the papers which mentioned subsequent treatments (14/27, 52%), the majority (9/14, 64%) classed them as a limitation for the interpretation of OS in clinical trials [28–36]. One paper (1/14, 7%) stated that they were not a limitation for interpretation if they reflected the care patients would receive after the trial [37] and four papers (4/14, 29%) did not mention subsequent treatments in this context [38–41].

## Discussion

This review identified 134 papers which had reported or discussed OS in the last decade with the aim to determine how it was currently analysed and if subsequent treatment lines were being accounted for and/or discussed.

The main finding in terms of the analysis of OS is that whilst it is generally defined consistently as an endpoint, and is commonly visualised with the Kaplan-Meier curve, the primary analysis is not as consistent as might have been expected. The authors expected that the majority of papers would report the results of a Cox proportional hazards model adjusted for at least the trial’s stratification factors, where both the hazard ratio and the *p*-value from the adjusted model would be used to interpret OS. This would be in line with guidance in ICH E9 which states, “If one or more factors are used to stratify the design, it is appropriate to account for those factors in the analysis” [2]. However, this was not the case. The majority of authors used a combination of a Cox model and a logrank test to interpret their primary analysis and make their conclusions. Whilst this may be statistically sound, it could be considered inconsistent as the confidence interval around the hazard ratio for the Cox model does not directly relate to the *p*-value from the logrank test as it would under the laws of duality if the *p*-value from the Cox model was used. It was reassuring to see that where a Cox model was included in the text, as a secondary analysis for OS, the majority of papers did report that it was adjusted for some or all of the stratification factors. This was less clear when a logrank test was presented. This is unexpected given that ICH E9 was first published in 1998, before the exclusion publication date for this literature review. In order to encourage consistency and transparency in reporting researchers in the future should consider planning and describing their analysis using the estimand framework proposed within ICHE9 (R1) [42]. These results are consistent with the overview of reporting survival analysis endpoints by Batson et al. [43] which was identified as a methodology/review paper in the search (ORN 209). In the paper, Batson and colleagues concluded that the majority of papers identified in their review reported the results of a Cox model and a logrank test with varying levels of adjustment. However, it is important to note that they were focusing on time-to-event endpoints broadly rather than just OS. It is also interesting that whilst a recent survey found that respondents consider adding a confidence interval to a Kaplan-Meier plot useful [3] no papers included in this review included confidence intervals.

The findings around subsequent treatment lines were less surprising. As whilst the majority which mentioned subsequent treatment lines summarised the types of treatments being received. A finding which complements a recent review by Oliver et al. [44] which was conducted over a shorter time period. Only seven papers, five of which were primary or interim results papers [10, 20–23], implemented some form of analysis to account for anti-cancer treatment received during trial follow-up. The majority of these only considered when participants switched to the alternative trial intervention and only two used methods akin to that described by Latimer et al. [5] (censoring at point of switch [21], sequencing models [12]). However, both were published prior to these recommendations. The work conducted by Penichoux et al. [12] using a shared frailty model to model progression and toxicity events as repeating events seems novel and does not seem to have received a lot of attention with only three papers citing this work. One is reporting the results of a single-arm study so is not relevant to this review [45]. One of the remaining citations by Malka et al. [46] is an editorial piece which references Penichoux’s work as an example of how to use random effects to address patients receiving the same intervention at different treatment lines. The final paper to cite Penichoux’s work is relevant to this research. Petracci et al. [47] used recurrent event analysis to assess the overall effectiveness of an intervention, in terms of progression-free survival, when it could have been used in first or second line by trial design. In the paper, the authors propose OS as an area for future work. There is a question as to whether this is restricted to the context of trials comparing specific sequences of treatment or whether it could be applied to account for subsequent lines of therapy that are not defined by trial design. As whilst Latimer et al. [5] suggest that sequencing models are a naïve approach given the need for data on each treatment line is hard to come by, there is a question as to whether this is still the case given the utilisation of routine data and patients awareness of how their data can be used through strategies such as DATA-CAN. In terms of the papers which discussed subsequent treatment lines, the most common discussion point was that uptake of subsequent treatments may have reduced the OS effect, affected the results or used to caveat the results. Interestingly the lack of subsequent treatment lines was also given as a reason for no OS effect. Whilst we agree with Oliver et al. that each paper reporting OS should summarise subsequent anti-cancer treatment [44]. We believe that descriptive statistics alone are not sufficient to support the interpretation of OS when patients receive anti-cancer therapy during trial follow-up. Instead, methodological work is required to develop estimation techniques to disentangle, if present, the effects of the trial experimental treatment and subsequent treatment lines.

Conversely, whilst only nine cost-effectiveness papers were identified they did seem to have a consistent approach to their primary analysis. In addition, where it was clear, the majority of papers did include subsequent treatment costs with a good proportion going further and including additional analysis which adjusted the OS estimate for subsequent treatment lines. Given the contrast to the clinical effectiveness papers, there is a question as to whether adjusting for subsequent lines is being left to be considered by those that evaluate the intervention for HTA review, which often requires cost-effectiveness to be fully analysed, rather than at the publication of the primary clinical effectiveness results.

The majority of the identified methodology/review papers were discussing treatment as a whole and did not consider subsequent lines in the context being considered here. As expected, a number of the methodology papers mentioned subsequent lines as a reason for assessing surrogate endpoints in their field [28, 30, 35] as this is in line with existing guidance [4]. This may also explain the shift in OS from a primary to secondary endpoint as seen in the analysis of the clinical effectiveness papers. In addition, one of the papers published in 2011 stated that subsequent lines are only an issue for the interpretation of clinical trial results if they fall outside of standard of care (SoC) [37]. Whilst this may be true, cancer research has accelerated in the last decade with more varied treatment options being available to patients. For example, in England, there are calls for guidance to be published quickly, and appraisals to be conducted efficiently, so that patients can get access to new treatments faster as part of the NHS, where, once approved, treatments should be available to patients within three months [48]. Currently there are 377 trials in cancer listed as “ongoing” on the ISCRTN database [49]. In addition, between 1 Jan 2021 and 1 Jan 2022, there were 35 technology appraisal guidelines published by NICE to advise on the treatment of cancer [50]. This is more than four times the eight appraisals which were published in between 1 Jan 2010 and 1 Jan 2011 [51]. This rapidly evolving clinical landscape means that the SoC is changing during the trial timeline and will continue to change after the trial closes and the treatment is incorporated into standard practice. Therefore, if OS is analysed without consideration of the subsequent lines, it is possible that the results will soon be invalid after publication. Methodological research is needed now to better account for subsequent lines so that the true effect of the new experimental intervention in trials is understood and considered when interventions are reviewed for use in SoC. This would work towards ensuring that interventions are properly evaluated so that beneficial interventions are not rejected due to subsequent lines diminishing any effect, and the time and resources it takes for a phase III confirmatory trial to report OS results are not wasted.

This review provides evidence that whilst OS is less frequently the primary endpoint, it is still of interest, and even when treatment switching is not the case, subsequent treatments are being considered as a limitation. This is the same across the different perspectives from the clinical effectiveness papers to the methodological and cost-effectiveness papers. At present, the approach to the assessment of both OS and subsequent treatment lines is not consistent. Whilst there will never be a one size fits all to the analysis of clinical trials, there is a need for an approach to subsequent treatment lines to aid interpretation of clinical trial results as more interventions are developed and recommended for use in standard or care.

There are some limitations to this systematic review. As there was only one researcher conducting the review, the more general search to gauge practice was restricted to only include top UK and international journals. It may be that the exploratory analysis which we are interested in here is reported in other, less-prominent journals. However, this was accounted for in the two approaches where the first search was not restricted by journal. In addition, articles prior to 2010 were excluded from the search. However, this is considered to be less of an issue as the technology support document advocating accounting for subsequent lines in the form of treatment switching was published in 2014 [5]. Having an additional researcher conduct and read all the results may have also helped to overcome some of the inconsistencies in interpretation of the terminology. As whilst all uncertainties were discussed with DAC, in some cases it was unclear whether stratification of a Cox model referred to applying different baseline hazards for different strata or including the variable in the model as an independent variable (the latter more commonly referred to as adjustment). However, the logrank tests were also reported to be adjusted when they can only be stratified. Finally, only papers which were written in English and available through free-text or institutional access were included. Papers written in other languages or behind a paywall may also have considered subsequent treatment lines and how they impact on the analysis of OS.

## Conclusion

Over the last decade, OS has changed from being the primary endpoint of cancer clinical trials to more commonly a secondary endpoint. Despite this, subsequent treatment lines in relation to OS are being consistently mentioned in articles, confirming that whilst OS may not be the primary endpoint, the analysis and results are still of interest. The majority of articles concluded that subsequent lines were a limitation of the interpretation of their OS analysis. However, analysis accounting for subsequent lines was generally only conducted in the event of treatment switching. The implemented methods were often naïve, and researchers did not utilise the more sophisticated and less well-known methods of IPCW or the RPSFTM. In fact, RPSFTM was only observed in one paper aiming to determine cost-effectiveness rather than clinical effectiveness. There is a question of whether researchers are aware of these more advanced methods (a possible educational gap) or whether they are just not being published (a reporting gap). This will be explored through the next stage of this research, in which stakeholders (patients and the public, statisticians and other data analysts, healthcare professionals, payers and industry partners) will be invited to complete a questionnaire on their views of OS and its analysis. Within the questionnaire, statisticians and other data analysts (e.g. health economists) will be asked to answer whether they have heard of and whether they have applied the various methods to analyse OS in cancer clinical trials. The overarching aim of the questionnaire will be to agree a consensus across the stakeholder groups in terms of the direction of this research and how statistical methodology should be extended or developed to account for subsequent treatment lines in the analysis of cancer clinical trials.

### Supplementary Information

**Additional file 1: Supplementary Table 1.** PRISMA Abstract Checklist. **Supplementary Table 2.** PRISMA Reporting Checklist. **Supplementary Table 3. **Summarised clinical effectiveness papers. **Supplementary Table 4.** Summary of the clinical effectiveness papers by year of publication. **Supplementary Table 5.** Summary of the Effectiveness papers by cancer. **Supplementary Table 6.** Summarised cost-effectiveness papers. **Supplementary Table 7.** Summarised Methodology / Review papers.

## Data Availability

The material from this literature review is available upon request.

## Abbreviations

BMJ: British Medical Journal
HTA: Health Technology Assessment
ICH: International Council for Harmonisation
IPCW: Inverse Probability Censoring Weights
ISCRTN: International Standard Randomised Controlled Trial Number
JAMA: Journal of the American Medical Association
JCO: Journal of Clinical Oncology
NHS: National Health Service
NICE: National Institute for Health and Care Excellence
OS: Overall Survival
PLOS: Public Library of Science
PRISMA: Preferred Reporting Items for Systematic Reviews and Meta-Analyses
RPSFTM: Rank preserving structural failure time model
SoC: Standard of care
UK: United Kingdom
USA: United States of America
