# Supplementary material for: How is overall survival assessed in randomised clinical trials in cancer and are subsequent treatment lines considered? A systematic review
Source: Trials. 2023 Nov 6;24:708. doi: 10.1186/s13063-023-07730-1 (PMC10626781; doi:10.1186/s13063-023-07730-1)
Supplement: Supplementary file 1 — Additional file 1: Supplementary Table 1. PRISMA Abstract Checklist. Supplementary Table 2. PRISMA Reporting Checklist. Supplementary Table 3. Summarised clinical effectiveness papers. Supplementary Table 4. Summary of the clinical effectiveness papers by year of publication. Supplementary Table 5. Summary of the Effectiveness papers by cancer. Supplementary Table 6. Summarised cost-effectiveness papers. Supplementary Table 7. Summarised Methodology / Review papers. [file 13063_2023_7730_MOESM1_ESM.docx]

# How is overall survival assessed in randomised clinical trials in cancer and are subsequent treatment lines considered? A systematic review – Supplementary Material

Supplementary Table 1: PRISMA Abstract Checklist

| **Section and Topic** | **Item #** | **Checklist item** | **Reported (Yes/No)** | |
| --- | --- | --- | --- | --- |
| **TITLE** | | | |  |
| Title | 1 | Identify the report as a systematic review. | Yes | |
| **BACKGROUND** | | | |  |
| Objectives | 2 | Provide an explicit statement of the main objective(s) or question(s) the review addresses. | Yes | |
| **METHODS** | | | |  |
| Eligibility criteria | 3 | Specify the inclusion and exclusion criteria for the review. | Yes | |
| Information sources | 4 | Specify the information sources (e.g. databases, registers) used to identify studies and the date when each was last searched. | Yes | |
| Risk of bias | 5 | Specify the methods used to assess risk of bias in the included studies. | N/A | |
| Synthesis of results | 6 | Specify the methods used to present and synthesise results. | N/A | |
| **RESULTS** | | | |  |
| Included studies | 7 | Give the total number of included studies and participants and summarise relevant characteristics of studies. | Yes | |
| Synthesis of results | 8 | Present results for main outcomes, preferably indicating the number of included studies and participants for each. If meta-analysis was done, report the summary estimate and confidence/credible interval. If comparing groups, indicate the direction of the effect (i.e. which group is favoured). | Yes | |
| **DISCUSSION** | | | |  |
| Limitations of evidence | 9 | Provide a brief summary of the limitations of the evidence included in the review (e.g. study risk of bias, inconsistency and imprecision). | Yes | |
| Interpretation | 10 | Provide a general interpretation of the results and important implications. | Yes | |
| **OTHER** | | | |  |
| Funding | 11 | Specify the primary source of funding for the review. | Yes | |
| Registration | 12 | Provide the register name and registration number. | N/A | |

*From:*  Page MJ, McKenzie JE, Bossuyt PM, Boutron I, Hoffmann TC, Mulrow CD, et al. The PRISMA 2020 statement: an updated guideline for reporting systematic reviews. BMJ 2021;372:n71. doi: 10.1136/bmj.n71

For more information, visit: <http://www.prisma-statement.org/>

Supplementary Table 2: PRISMA Reporting Checklist

| **Section and Topic** | **Item #** | **Checklist item** | **Location where item is reported** |
| --- | --- | --- | --- |
| **TITLE** | | |  |
| Title | 1 | Identify the report as a systematic review. | Page 1 |
| **ABSTRACT** | | |  |
| Abstract | 2 | See the PRISMA 2020 for Abstracts checklist. | Supplementary Table 1 |
| **INTRODUCTION** | | |  |
| Rationale | 3 | Describe the rationale for the review in the context of existing knowledge. | Page 4 |
| Objectives | 4 | Provide an explicit statement of the objective(s) or question(s) the review addresses. | Page 4 |
| **METHODS** | | |  |
| Eligibility criteria | 5 | Specify the inclusion and exclusion criteria for the review and how studies were grouped for the syntheses. | Figure 1 |
| Information sources | 6 | Specify all databases, registers, websites, organisations, reference lists and other sources searched or consulted to identify studies. Specify the date when each source was last searched or consulted. | Page 4 - 5 |
| Search strategy | 7 | Present the full search strategies for all databases, registers and websites, including any filters and limits used. | Table 1 |
| Selection process | 8 | Specify the methods used to decide whether a study met the inclusion criteria of the review, including how many reviewers screened each record and each report retrieved, whether they worked independently, and if applicable, details of automation tools used in the process. | Page 5 |
| Data collection process | 9 | Specify the methods used to collect data from reports, including how many reviewers collected data from each report, whether they worked independently, any processes for obtaining or confirming data from study investigators, and if applicable, details of automation tools used in the process. | Page 5 |
| Data items | 10a | List and define all outcomes for which data were sought. Specify whether all results that were compatible with each outcome domain in each study were sought (e.g. for all measures, time points, analyses), and if not, the methods used to decide which results to collect. | Table 2 |
|  | 10b | List and define all other variables for which data were sought (e.g. participant and intervention characteristics, funding sources). Describe any assumptions made about any missing or unclear information. | Table 2 |
| Study risk of bias assessment | 11 | Specify the methods used to assess risk of bias in the included studies, including details of the tool(s) used, how many reviewers assessed each study and whether they worked independently, and if applicable, details of automation tools used in the process. | Not a meta-analysis |
| Effect measures | 12 | Specify for each outcome the effect measure(s) (e.g. risk ratio, mean difference) used in the synthesis or presentation of results. | Page 5 |
| Synthesis methods | 13a | Describe the processes used to decide which studies were eligible for each synthesis (e.g. tabulating the study intervention characteristics and comparing against the planned groups for each synthesis (item #5)). | Not a meta-analysis |
|  | 13b | Describe any methods required to prepare the data for presentation or synthesis, such as handling of missing summary statistics, or data conversions. | Not a meta-analysis |
|  | 13c | Describe any methods used to tabulate or visually display results of individual studies and syntheses. | Not a meta-analysis |
|  | 13d | Describe any methods used to synthesize results and provide a rationale for the choice(s). If meta-analysis was performed, describe the model(s), method(s) to identify the presence and extent of statistical heterogeneity, and software package(s) used. | Not a meta-analysis |
|  | 13e | Describe any methods used to explore possible causes of heterogeneity among study results (e.g. subgroup analysis, meta-regression). | Not a meta-analysis |
|  | 13f | Describe any sensitivity analyses conducted to assess robustness of the synthesized results. | Not a meta-analysis |
| Reporting bias assessment | 14 | Describe any methods used to assess risk of bias due to missing results in a synthesis (arising from reporting biases). | Not a meta-analysis |
| Certainty assessment | 15 | Describe any methods used to assess certainty (or confidence) in the body of evidence for an outcome. | Not a meta-analysis |
| **RESULTS** | | |  |
| Study selection | 16a | Describe the results of the search and selection process, from the number of records identified in the search to the number of studies included in the review, ideally using a flow diagram. | Figure 1 |
|  | 16b | Cite studies that might appear to meet the inclusion criteria, but which were excluded, and explain why they were excluded. | Figure 1 |
| Study characteristics | 17 | Cite each included study and present its characteristics. | Figure 1 |
| Risk of bias in studies | 18 | Present assessments of risk of bias for each included study. | Not a meta-analysis |
| Results of individual studies | 19 | For all outcomes, present, for each study: (a) summary statistics for each group (where appropriate) and (b) an effect estimate and its precision (e.g. confidence/credible interval), ideally using structured tables or plots. | Not a meta-analysis |
| Results of syntheses | 20a | For each synthesis, briefly summarise the characteristics and risk of bias among contributing studies. | Not a meta-analysis |
|  | 20b | Present results of all statistical syntheses conducted. If meta-analysis was done, present for each the summary estimate and its precision (e.g. confidence/credible interval) and measures of statistical heterogeneity. If comparing groups, describe the direction of the effect. | Not a meta-analysis |
|  | 20c | Present results of all investigations of possible causes of heterogeneity among study results. | Not a meta-analysis |
|  | 20d | Present results of all sensitivity analyses conducted to assess the robustness of the synthesized results. | Not a meta-analysis |
| Reporting biases | 21 | Present assessments of risk of bias due to missing results (arising from reporting biases) for each synthesis assessed. | Not a meta-analysis |
| Certainty of evidence | 22 | Present assessments of certainty (or confidence) in the body of evidence for each outcome assessed. | Not a meta-analysis |
| **DISCUSSION** | | |  |
| Discussion | 23a | Provide a general interpretation of the results in the context of other evidence. | Page 8 - 11 |
|  | 23b | Discuss any limitations of the evidence included in the review. | Page 10 - 11 |
|  | 23c | Discuss any limitations of the review processes used. | Page 10 - 11 |
|  | 23d | Discuss implications of the results for practice, policy, and future research. | Page 11 |
| **OTHER INFORMATION** | | |  |
| Registration and protocol | 24a | Provide registration information for the review, including register name and registration number, or state that the review was not registered. | Not a meta-analysis |
|  | 24b | Indicate where the review protocol can be accessed, or state that a protocol was not prepared. | Not prepared |
|  | 24c | Describe and explain any amendments to information provided at registration or in the protocol. | N/A |
| Support | 25 | Describe sources of financial or non-financial support for the review, and the role of the funders or sponsors in the review. | Page 12 |
| Competing interests | 26 | Declare any competing interests of review authors. | Page 12 |
| Availability of data, code and other materials | 27 | Report which of the following are publicly available and where they can be found: template data collection forms; data extracted from included studies; data used for all analyses; analytic code; any other materials used in the review. | Page 12 |

*From:*  Page MJ, McKenzie JE, Bossuyt PM, Boutron I, Hoffmann TC, Mulrow CD, et al. The PRISMA 2020 statement: an updated guideline for reporting systematic reviews. BMJ 2021;372:n71. doi: 10.1136/bmj.n71

For more information, visit: <http://www.prisma-statement.org/>

Supplementary Table 3: Summarised clinical effectiveness papers

| **ORN** | **Title** | **Year** | **Cancer** | **OS Endpoint** | **Primary analysis** | **Was a Kaplan-Meier curve for OS presented in the main text?** | **Were the numbers at risk included in the Kaplan-Meier curve?** | **Were the number of patients censored or number of events included in the at risk table** | **was a logrank test for OS presented in the main text?** | **If included, was an logrank test for OS presented using unadjusted or adjusted or both techniques in the main text (separate from subgroup analysis)?** | **If included, what level of adjustment was considered for an logrank test for OS in the main text (separate from subgroup analysis)?** | **Was a Cox-PH Model for OS presented in the main text (separate from subgroup analysis)?** | **If included, was a Cox-PH Model for OS presented using unadjusted or adjusted or both techniques in the main text (separate from subgroup analysis)?** | **If included, what level of adjustment was considered for a Cox-PH Model for OS in the main text (separate from subgroup analysis)?** | **Were subsequent treatments mentioned in the paper?** | **Was the percentage or number of participants who received later lines included in the main text or appendix?** | **Was a breakdown or summary of the number of subsequent treatments (Two, Three, Four) included in the main text or appendix?** | **Was a breakdown or summary of the type of subsequent treatments (Treatment 1, Treatment 2, Treatment 3) included in the main text or appendix?** | **Were subsequent treatment lines mentioned in the discussion?** | **How were subsequent treatments mentioned in the discussion?** | **Was additional analysis conducted to account for subsequent treatment lines?** |
| --- | --- | --- | --- | --- | --- | --- | --- | --- | --- | --- | --- | --- | --- | --- | --- | --- | --- | --- | --- | --- | --- |
| 1 | Hepatectomy Followed by mFOLFOX6 Versus Hepatectomy Alone for Liver-Only Metastatic Colorectal Cancer (JCOG0603): A Phase II or III Randomized Controlled Trial. ^1^ | 2021 | Bowel / Lower GI | Secondary | Hazard ratio from an adjusted Cox model and p-value from an stratified logrank test. | Yes | Yes | No | Yes | Adjusted Analysis | Yes - Unclear on what adjusted for | Yes | Adjusted Analysis | Yes - Unclear on what adjusted for | Yes | Yes | No | Yes | Yes | The uptake of subsequent treatments is given as a reason for the discrepancy between OS and surrogate endpoints. | No |
| 2 | Late Toxicity After Adjuvant Conventional Radiation Versus Image-Guided Intensity-Modulated Radiotherapy for Cervical Cancer (PARCER): A Randomized Controlled Trial. ^2^ | 2021 | Gynaecological | Secondary | Hazard ratio and P-value from a logrank test | No | N/A | N/A | Yes | Not Specified | Unsure - Not Specified | No | N/A | N/A | No | N/A | N/A | N/A | N/A | N/A | N/A |
| 5 | Five-Year Outcomes With Pembrolizumab Versus Chemotherapy for Metastatic Non-Small-Cell Lung Cancer With PD-L1 Tumor Proportion Score >= 50. ^3^ | 2021 | Lung | Secondary | Hazard ratio and 95% CI from an adjusted Cox model | Yes | Yes | No | No | N/A | N/A | Yes | Adjusted Analysis | Yes - Adjusted for some or all stratification factors | Yes | Yes | No | Yes | Yes | The uptake of subsequent treatments is given as a reason for reduced OS effect / stated as may have affected the results / used to caveat the results. | No |
| 7 | Modified FOLFIRINOX versus S-1 as second-line chemotherapy in gemcitabine-failed metastatic pancreatic cancer patients: A randomised controlled trial (MPACA-3). ^4^ | 2021 | Upper GI/HPB | Primary or Co-Primary | Hazard ratio and p-value from an adjusted Cox model | Yes | Yes | No | Yes | Unadjusted Analysis | No - Only Unadjusted analysis | Yes | Both Adjusted and Unadjusted Analysis | Yes - Adjusted for non-stratification factors | Yes | Yes | No | Yes | Yes | The uptake of subsequent treatments is given as a reason for reduced OS effect / stated as may have affected the results / used to caveat the results. | No |
| 13 | Bevacizumab Plus mFOLFOX6 Versus mFOLFOX6 Alone as First-Line Treatment for RAS Mutant Unresectable Colorectal Liver-Limited Metastases: The BECOME Randomized Controlled Trial. ^5^ | 2020 | Bowel / Lower GI | Secondary | Hazard ratio and P-value from a logrank test | Yes | Yes | No | Yes | Not Specified | Unsure - Not Specified | No | N/A | N/A | Yes | No | No | No | Yes | The uptake of subsequent treatments is given as a reason for reduced OS effect / stated as may have affected the results / used to caveat the results. | No |
| 23 | Bevacizumab biosimilar LY01008 compared with bevacizumab (Avastin) as first-line treatment for Chinese patients with unresectable, metastatic, or recurrent non-squamous non-small-cell lung cancer: A multicenter, randomized, double-blinded, phase III trial. ^6^ | 2021 | Lung | Secondary | Other (Median OS and logrank p-value.) | Yes | Yes | No | Yes | Not Specified | Unsure - Not Specified | No | N/A | N/A | No | N/A | N/A | N/A | N/A | N/A | N/A |
| 24 | Prostate-Only Versus Whole-Pelvic Radiation Therapy in High-Risk and Very High-Risk Prostate Cancer (POP-RT): Outcomes From Phase III Randomized Controlled Trial. ^7^ | 2021 | Genito-urinary | Secondary | Hazard ratio from a Cox model and p-value from a logrank test | Yes | Yes | No | Yes | Not Specified | Unsure - Not Specified | Yes | Unadjusted Analysis | No - Only Unadjusted analysis | No | N/A | N/A | N/A | N/A | N/A | N/A |
| 37 | Second-line FOLFOX chemotherapy versus active symptom control for advanced biliary tract cancer (ABC-06): a phase 3, open-label, randomised, controlled trial. ^8^ | 2021 | Upper GI/HPB | Primary or Co-Primary | Hazard ratio and p-value from an adjusted Cox model | Yes | Yes | Yes | No | N/A | N/A | Yes | Adjusted Analysis | Yes - Adjusted for some or all stratification factors | Yes | Yes | Yes | Yes | Yes | Randomisation / balance of subsequent treatment lines is given as a reason as to why subsequent treatment lines will not have affected the OS results. | No |
| 45 | Intracranial Efficacy and Survival With Tucatinib Plus Trastuzumab and Capecitabine for Previously Treated HER2-Positive Breast Cancer With Brain Metastases in the HER2CLIMB Trial. ^9^ | 2020 | Breast | Exploratory | Hazard ratio from an adjusted Cox model and p-value from an stratified logrank test. | Yes | Yes | No | Yes | Adjusted Analysis | Yes - Unclear on what adjusted for | Yes | Adjusted Analysis | Yes - Adjusted for some or all stratification factors | No | N/A | N/A | N/A | N/A | N/A | N/A |
| 52 | Sunitinib Versus Sorafenib as Initial Targeted Therapy for mCC-RCC With Favorable/Intermediate Risk: Multicenter Randomized Trial CROSS-J-RCC. ^10^ | 2020 | Genito-urinary | Secondary | Hazard ratio from a Cox model and p-value from a logrank test | Yes | Yes | No | Yes | Not Specified | Unsure - Not Specified | Yes | Unadjusted Analysis | No - Only Unadjusted analysis | Yes | Yes | Yes | Yes | Yes | None or limited options of subsequent treatment lines for patients are given as a reason as to why subsequent treatment lines will not have affected the OS results. | No |
| 54 | Cemiplimab monotherapy for first-line treatment of advanced non-small-cell lung cancer with PD-L1 of at least 50%: a multicentre, open-label, global, phase 3, randomised, controlled trial. ^11^ | 2021 | Lung | Primary or Co-Primary | Hazard ratio from an adjusted Cox model and p-value from an stratified logrank test. | Yes | Yes | Yes | Yes | Adjusted Analysis | Yes - Adjusted for some or all stratification factors | Yes | Adjusted Analysis | Yes - Adjusted for some or all stratification factors | Yes | Yes | No | Yes | Yes | The uptake of subsequent treatments is given as a reason for reduced OS effect / stated as may have affected the results / used to caveat the results. | No |
| 67 | Metronomic capecitabine as maintenance treatment after first line induction with XELOX for metastatic colorectal cancer patients. ^12^ | 2020 | Bowel / Lower GI | Secondary | Hazard ratio from a Cox model and p-value from a logrank test | Yes | No | N/A | Yes | Not Specified | Unsure - Not Specified | Yes | Unadjusted Analysis | No - Only Unadjusted analysis | Yes | Yes | No | Yes | Yes | The uptake of subsequent treatments is given as a reason for reduced OS effect / stated as may have affected the results / used to caveat the results. | No |
| 69 | Carfilzomib or bortezomib in combination with lenalidomide and dexamethasone for patients with newly diagnosed multiple myeloma without intention for immediate autologous stem-cell transplantation (ENDURANCE): a multicentre, open-label, phase 3, randomised, controlled trial. ^13^ | 2020 | Haematological | Secondary | Hazard ratio from an adjusted Cox model and p-value from an stratified logrank test. | Yes | Yes | Yes | Yes | Adjusted Analysis | Yes - Unclear on what adjusted for | Yes | Adjusted Analysis | Yes - Unclear on what adjusted for | No | N/A | N/A | N/A | N/A | N/A | N/A |
| 71 | Sequencing of Androgen-Deprivation Therapy With External-Beam Radiotherapy in Localized Prostate Cancer: A Phase III Randomized Controlled Trial. ^14^ | 2020 | Genito-urinary | Secondary | Hazard ratio from an unadjusted cox model and p-value from an stratified logrank test. | Yes | Yes | No | Yes | Adjusted Analysis | Yes - Adjusted for some or all stratification factors | Yes | Adjusted Analysis | Yes - Adjusted for some or all stratification factors | No | N/A | N/A | N/A | N/A | N/A | N/A |
| 78 | Liver transplantation in hepatocellular carcinoma after tumour downstaging (XXL): a randomised, controlled, phase 2b/3 trial. ^15^ | 2020 | Upper GI/HPB | Primary or Co-Primary | Hazard ratio from an adjusted Cox model and p-value from an stratified logrank test. | Yes | Yes | Yes | Yes | Adjusted Analysis | Yes - Adjusted for some or all stratification factors | Yes | Adjusted Analysis | Yes - Adjusted for some or all stratification factors | No | N/A | N/A | N/A | N/A | N/A | N/A |
| 82 | Weekly dose-dense chemotherapy in first-line epithelial ovarian, fallopian tube, or primary peritoneal carcinoma treatment (ICON8): primary progression free survival analysis results from a GCIG phase 3 randomised controlled trial. ^16^ | 2019 | Gynaecological | Primary or Co-Primary | N/A | N/A | N/A | N/A | N/A | N/A | N/A | N/A | N/A | N/A | No | N/A | N/A | N/A | N/A | N/A | N/A |
| 91 | Oxaliplatin-Based Adjuvant Chemotherapy for Rectal Cancer After Preoperative Chemoradiotherapy (ADORE): Long-Term Results of a Randomized Controlled Trial. ^17^ | 2019 | Bowel / Lower GI | Secondary | Hazard ratio and p-value from an adjusted Cox model | Yes | Yes | No | No | N/A | N/A | Yes | Adjusted Analysis | Yes - Adjusted for some or all stratification factors | Yes | Yes | No | Yes | No | N/A | No |
| 96 | The effect of modulated electro-hyperthermia on local disease control in HIV-positive and -negative cervical cancer women in South Africa: Early results from a phase III randomised controlled trial. ^18^ | 2019 | Gynaecological | Secondary | N/A | N/A | N/A | N/A | N/A | N/A | N/A | N/A | N/A | N/A | No | N/A | N/A | N/A | N/A | N/A | N/A |
| 97 | Combination of icotinib and chemotherapy as first-line treatment for advanced lung adenocarcinoma in patients with sensitive EGFR mutations: A randomized controlled study. ^19^ | 2019 | Lung | Secondary | Hazard ratio and P-value from a logrank test | Yes | Yes | No | Yes | Not Specified | Unsure - Not Specified | No | N/A | N/A | Yes | Yes | No | Yes | Yes | The uptake of subsequent treatments is given as a reason for reduced OS effect / stated as may have affected the results / used to caveat the results. | No |
| 98 | Erlotinib Versus Gemcitabine Plus Cisplatin as Neoadjuvant Treatment of Stage IIIA-N2 EGFR-Mutant Non-Small-Cell Lung Cancer (EMERGING-CTONG 1103): A Randomized Phase II Study. ^20^ | 2019 | Lung | Secondary | Hazard ratio from a Cox model and p-value from a logrank test | Yes | Yes | No | Yes | Unadjusted Analysis | No - Only Unadjusted analysis | Yes | Unadjusted Analysis | No - Only Unadjusted analysis | No | N/A | N/A | N/A | N/A | N/A | N/A |
| 103 | Atezolizumab plus bevacizumab versus sunitinib in patients with previously untreated metastatic renal cell carcinoma (IMmotion151): a multicentre, open-label, phase 3, randomised controlled trial. ^21^ | 2019 | Genito-urinary | Primary or Co-Primary | Hazard ratio from an adjusted Cox model and p-value from an stratified logrank test. | Yes | Yes | Yes | Yes | Adjusted Analysis | Yes - Adjusted for some or all stratification factors | Yes | Adjusted Analysis | Yes - Adjusted for some or all stratification factors | Yes | Yes | No | Yes | No | N/A | No |
| 105 | Atezolizumab with or without cobimetinib versus regorafenib in previously treated metastatic colorectal cancer (IMblaze370): a multicentre, open-label, phase 3, randomised, controlled trial. ^22^ | 2019 | Bowel / Lower GI | Primary or Co-Primary | Hazard ratio and P-value from a stratified logrank test. | Yes | Yes | Yes | Yes | Adjusted Analysis | Yes - Unclear on what adjusted for | Yes | Adjusted Analysis | Yes - Unclear on what adjusted for | Yes | Yes | No | Yes | No | N/A | No |
| 126 | Randomized Trial of Systemic Therapy After Involved-Field Radiotherapy in Patients With Early-Stage Follicular Lymphoma: TROG 99.03. ^23^ | 2018 | Haematological | Secondary | Hazard ratio and P-value from a logrank test | Yes | Yes | No | Yes | Not Specified | Unsure - Not Specified | No | N/A | N/A | Yes | No | No | No | Yes | The uptake of subsequent treatments is given as a reason for better OS results or a reduced event rate than expected. | No |
| 135 | A randomized, controlled trial evaluating the efficacy and safety of BTH1677 in combination with bevacizumab, carboplatin, and paclitaxel in first-line treatment of advanced non-small cell lung cancer. ^24^ | 2018 | Lung | Secondary | Hazard ratio and P-value from a logrank test | Yes | Yes | No | Unsure | N/A | N/A | No | N/A | N/A | No | N/A | N/A | N/A | N/A | N/A | N/A |
| 138 | Neoadjuvant Chemotherapy Followed by Radical Surgery Versus Concomitant Chemotherapy and Radiotherapy in Patients With Stage IB2, IIA, or IIB Squamous Cervical Cancer: A Randomized Controlled Trial. ^25^ | 2018 | Gynaecological | Secondary | Hazard ratio and P-value from a logrank test | Yes | Yes | No | Yes | Not Specified | Unsure - Not Specified | Yes | Adjusted Analysis | Yes - Adjusted for both strat and non-strat factors | Yes | Yes | No | Yes | No | N/A | No |
| 142 | Bevacizumab Maintenance Versus No Maintenance During Chemotherapy-Free Intervals in Metastatic Colorectal Cancer: A Randomized Phase III Trial (PRODIGE 9). ^26^ | 2018 | Bowel / Lower GI | Secondary | Hazard ratio and P-value from a logrank test | Yes | Yes | No | Yes | Unadjusted Analysis | No - Only Unadjusted analysis | Yes | Adjusted Analysis | Yes - Adjusted for some or all stratification factors | Yes | Yes | No | Yes | No | N/A | No |
| 144 | Atezolizumab versus chemotherapy in patients with platinum-treated locally advanced or metastatic urothelial carcinoma (IMvigor211): a multicentre, open-label, phase 3 randomised controlled trial. ^27^ | 2018 | Genito-urinary | Primary or Co-Primary | Hazard ratio and p-value from an adjusted Cox model | Yes | Yes | No | Yes | Not Specified | Unsure - Not Specified | Yes | Adjusted Analysis | Yes - Adjusted for some or all stratification factors | Yes | Yes | No | Yes | No | N/A | No |
| 152 | Adding Celecoxib With or Without Zoledronic Acid for Hormone-Naive Prostate Cancer: Long-Term Survival Results From an Adaptive, Multiarm, Multistage, Platform, Randomized Controlled Trial. ^28^ | 2017 | Genito-urinary | Primary or Co-Primary | Hazard ratio and p-value from an adjusted Cox model | Yes | Yes | Yes | No | N/A | N/A | Yes | Adjusted Analysis | Yes - Adjusted for some or all stratification factors | Yes | Yes | No | Yes | No | N/A | No |
| 153 | Randomized controlled trial of S-1 versus docetaxel in patients with non-small-cell lung cancer previously treated with platinum-based chemotherapy (East Asia S-1 Trial in Lung Cancer). ^29^ | 2017 | Lung | Primary or Co-Primary | Hazard ratio and p-value from an unadjusted Cox model | Yes | Yes | No | No | N/A | N/A | Yes | Both Adjusted and Unadjusted Analysis | Yes - Unclear on what adjusted for | Yes | Yes | No | Yes | No | N/A | No |
| 155 | Peri-operative chemotherapy with or without bevacizumab in operable oesophagogastric adenocarcinoma (UK Medical Research Council ST03): primary analysis results of a multicentre, open-label, randomised phase 2-3 trial. ^30^ | 2017 | Upper GI/HPB | Primary or Co-Primary | Hazard ratio and P-value from a logrank test | Yes | Yes | Yes | Yes | Unadjusted Analysis | No - Only Unadjusted analysis | No | N/A | N/A | No | N/A | N/A | N/A | N/A | N/A | N/A |
| 160 | Results of a phase II randomized controlled clinical trial comparing efficacy of Cabazitaxel versus Docetaxel as second line or above therapy in recurrent head and neck cancer. ^31^ | 2017 | Other | Secondary | Hazard ratio and p-value from an unadjusted Cox model | Yes | Yes | No | No | N/A | N/A | Yes | Unadjusted Analysis | No - Only Unadjusted analysis | No | N/A | N/A | N/A | N/A | N/A | N/A |
| 161 | Effect of docetaxel duration on clinical outcomes: exploratory analysis of CLEOPATRA, a phase III randomized controlled trial. ^32^ | 2017 | Breast | Exploratory | Hazard ratio and p-value from an adjusted Cox model | Yes | Yes | No | Yes | Adjusted Analysis | Yes - Unclear on what adjusted for | Yes | Adjusted Analysis | Yes - Unclear on what adjusted for | Yes | No | No | No | No | N/A | Yes (Time Dependent Covariate) |
| 166 | Carfilzomib or bortezomib in relapsed or refractory multiple myeloma (ENDEAVOR): an interim overall survival analysis of an open-label, randomised, phase 3 trial. ^33^ | 2017 | Haematological | Secondary | Hazard ratio from an adjusted Cox model and p-value from an stratified logrank test. | Yes | Yes | Yes | Yes | Adjusted Analysis | Yes - Unclear on what adjusted for | Yes | Adjusted Analysis | Yes - Unclear on what adjusted for | Yes | Yes | No | Yes | Yes | The uptake of subsequent treatments is given as a reason for reduced OS effect / stated as may have affected the results / used to caveat the results. | Yes (Landmark analysis to calculate post-progression survival) |
| 167 | Randomized Controlled Trial Testing the Efficacy of Platinum-Free Interval Prolongation in Advanced Ovarian Cancer: The MITO-8, MaNGO, BGOG-Ov1, AGO-Ovar2.16, ENGOT-Ov1, GCIG Study. ^34^ | 2017 | Gynaecological | Primary or Co-Primary | Hazard ratio and p-value from an adjusted Cox model | Yes | No | N/A | No | N/A | N/A | Yes | Adjusted Analysis | Yes - Adjusted for both strat and non-strat factors | No | N/A | N/A | N/A | N/A | N/A | N/A |
| 177 | Atezolizumab versus docetaxel in patients with previously treated non-small-cell lung cancer (OAK): a phase 3, open-label, multicentre randomised controlled trial. ^35^ | 2017 | Lung | Primary or Co-Primary | Hazard ratio from an adjusted Cox model and p-value from a logrank test. | Yes | Yes | No | Yes | Not Specified | Unsure - Not Specified | Yes | Adjusted Analysis | Yes - Adjusted for some or all stratification factors | Yes | Yes | No | Yes | Yes | The uptake of subsequent treatments is given as a reason for reduced OS effect / stated as may have affected the results / used to caveat the results. | No |
| 179 | A randomized trial of TLR-2 agonist CADI-05 targeting desmocollin-3 for advanced non-small-cell lung cancer. ^36^ | 2017 | Lung | Primary or Co-Primary | Hazard ratio and P-value from a stratified logrank test. | Yes | No | N/A | Yes | Adjusted Analysis | Yes - Unclear on what adjusted for | Yes | Adjusted Analysis | Yes - Adjusted for some or all stratification factors | Yes | Yes | N/A | N/A | No | N/A | N/A |
| 183 | Randomized controlled trial of lobaplatin plus etoposide vs. cisplatin plus etoposide as first-line therapy in patients with extensive-stage small cell lung cancer. ^37^ | 2019 | Lung | Secondary | Hazard ratio from an unadjusted cox model and p-value from a logrank test. | Yes | Yes | No | Yes | Not Specified | Unsure - Not Specified | Yes | Adjusted Analysis | Yes - Adjusted for some or all stratification factors | Yes | Yes | No | Yes | No | N/A | No |
| 187 | A Phase II Randomized Controlled Trial: Definitive Concurrent Chemoradiotherapy with Docetaxel Plus Cisplatin versus 5-Fluorouracil plus Cisplatin in Patients with Oesophageal Squamous Cell Carcinoma. ^38^ | 2017 | Upper GI/HPB | Primary or Co-Primary | Other (p-value from a logrank test with estimates of OS at 1 and 2 years) | Yes | No | N/A | Yes | Not Specified | Unsure - Not Specified | No | N/A | N/A | No | N/A | N/A | N/A | N/A | N/A | N/A |
| 192 | Addition of docetaxel, zoledronic acid, or both to first-line long-term hormone therapy in prostate cancer (STAMPEDE): survival results from an adaptive, multiarm, multistage, platform randomised controlled trial. ^39^ | 2016 | Genito-urinary | Primary or Co-Primary | Hazard ratio and p-value from an adjusted Cox model | Yes | Yes | Yes | No | N/A | N/A | Yes | Adjusted Analysis | Yes - Adjusted for some or all stratification factors | Yes | Yes | No | Yes | Yes | The uptake of subsequent treatments is given as a reason for better OS results or a reduced event rate than expected. | No |
| 199 | Combined nivolumab and ipilimumab versus ipilimumab alone in patients with advanced melanoma: 2-year overall survival outcomes in a multicentre, randomised, controlled, phase 2 trial. ^40^ | 2016 | Other | Exploratory | Hazard ratio from an adjusted Cox model and p-value from an stratified logrank test. | Yes | Yes | Yes | Yes | Adjusted Analysis | Yes - Adjusted for some or all stratification factors | Yes | Adjusted Analysis | Yes - Adjusted for some or all stratification factors | Yes | Yes | No | Yes | Yes | The uptake of subsequent treatments is given as a reason for better OS results or a reduced event rate than expected. | Yes (Censor point of subsequent therapies) |
| 201 | Adjuvant Oral Uracil-Tegafur with Leucovorin for Colorectal Cancer Liver Metastases: A Randomized Controlled Trial. ^41^ | 2016 | Bowel / Lower GI | Secondary | Hazard ratio from an adjusted Cox model and p-value from a logrank test. | Yes | Yes | No | Yes | Adjusted Analysis | Yes - Unclear on what adjusted for | Unsure | N/A | N/A | Yes | Yes | No | Yes | Yes | The uptake of subsequent treatments is given as a reason for reduced OS effect / stated as may have affected the results / used to caveat the results. | No |
| 202 | TRAPEZE: a randomised controlled trial of the clinical effectiveness and cost-effectiveness of chemotherapy with zoledronic acid, strontium-89, or both, in men with bony metastatic castration-refractory prostate cancer. ^42^ | 2016 | Genito-urinary | Secondary | Hazard ratio from an adjusted Cox model and p-value from an stratified logrank test. | Yes | Yes | No | Yes | Adjusted Analysis | Yes - Adjusted for non-stratification factors | Yes | Adjusted Analysis | Yes - Adjusted for some or all stratification factors | No | N/A | N/A | N/A | N/A | N/A | N/A |
| 208 | Salvage radiotherapy with or without short-term hormone therapy for rising prostate-specific antigen concentration after radical prostatectomy (GETUG-AFU 16): a randomised, multicentre, open-label phase 3 trial. ^43^ | 2016 | Genito-urinary | Secondary | Hazard ratio and P-value from a stratified logrank test. | No | N/A | N/A | Yes | Adjusted Analysis | Yes - Adjusted for some or all stratification factors | No | N/A | N/A | No | N/A | N/A | N/A | N/A | N/A | N/A |
| 213 | Afatinib versus gefitinib as first-line treatment of patients with EGFR mutation-positive non-small-cell lung cancer (LUX-Lung 7): a phase 2B, open-label, randomised controlled trial. ^44^ | 2016 | Lung | Primary or Co-Primary | N/A | N/A | N/A | N/A | N/A | N/A | N/A | N/A | N/A | N/A | Yes | Yes | No | Yes | Yes | The uptake of subsequent treatments is given as a reason for better OS results or a reduced event rate than expected. | No |
| 215 | Atezolizumab versus docetaxel for patients with previously treated non-small-cell lung cancer (POPLAR): a multicentre, open-label, phase 2 randomised controlled trial. ^45^ | 2016 | Lung | Primary or Co-Primary | Hazard ratio and p-value from an adjusted Cox model | Yes | Yes | No | Yes | Adjusted Analysis | Yes - Adjusted for some or all stratification factors | Yes | Adjusted Analysis | Yes - Adjusted for some or all stratification factors | Yes | Yes | No | Yes | No | N/A | No |
| 224 | Gastrectomy plus chemotherapy versus chemotherapy alone for advanced gastric cancer with a single non-curable factor (REGATTA): a phase 3, randomised controlled trial. ^46^ | 2016 | Upper GI/HPB | Primary or Co-Primary | Hazard ratio from an adjusted Cox model and p-value from an stratified logrank test. | Yes | Yes | No | Yes | Adjusted Analysis | Yes - Adjusted for non-stratification factors | Yes | Adjusted Analysis | Yes - Adjusted for non-stratification factors | Yes | Yes | No | Yes | Yes | Other (Cross-over trial design) | No |
| 226 | Pembrolizumab versus docetaxel for previously treated, PD-L1-positive, advanced non-small-cell lung cancer (KEYNOTE-010): a randomised controlled trial. ^47^ | 2016 | Lung | Primary or Co-Primary | Hazard ratio from an adjusted Cox model and p-value from an stratified logrank test. | Yes | Yes | No | Yes | Adjusted Analysis | Yes - Adjusted for some or all stratification factors | Yes | Adjusted Analysis | Yes - Adjusted for some or all stratification factors | Yes | Yes | No | Yes | Yes | Subsequent treatments are stated to not have affected the OS results as an OS benefit was observed or OS was similar between those who did and did not receive a subsequent treatment. | No |
| 234 | Standard chemotherapy with or without bevacizumab for women with newly diagnosed ovarian cancer (ICON7): overall survival results of a phase 3 randomised trial. ^48^ | 2015 | Gynaecological | Secondary | Other (P-value from a logrank test difference in restricted mean survival time at 5 years.) | Yes | Yes | No | Yes | Unadjusted Analysis | No - Only Unadjusted analysis | Yes | Not Specified | Unsure - Not Specified | Yes | No | No | No | Yes | Randomisation / balance of subsequent treatment lines is given as a reason as to why subsequent treatment lines will not have affected the OS results. | No |
| 235 | Methotrexate, Doxorubicin, and Cisplatin (MAP) Plus Maintenance Pegylated Interferon Alfa-2b Versus MAP Alone in Patients With Resectable High-Grade Osteosarcoma and Good Histologic Response to Preoperative MAP: First Results of the EURAMOS-1 Good Response Randomized Controlled Trial. ^49^ | 2015 | Other | Secondary | Hazard ratio from an adjusted Cox model and p-value from a logrank test. | Yes | Yes | Yes | Yes | Not Specified | Unsure - Not Specified | Yes | Adjusted Analysis | Yes - Adjusted for some or all stratification factors | No | N/A | N/A | N/A | N/A | N/A | N/A |
| 236 | Primary chemotherapy versus primary surgery for newly diagnosed advanced ovarian cancer (CHORUS): an open-label, randomised, controlled, non-inferiority trial. ^50^ | 2015 | Gynaecological | Primary or Co-Primary | Hazard ratio and 95% CI from an adjusted Cox model | Yes | Yes | No | Yes | Adjusted Analysis | Yes - Adjusted for some or all stratification factors | Yes | Adjusted Analysis | Yes - Adjusted for some or all stratification factors | No | N/A | N/A | N/A | N/A | N/A | N/A |
| 237 | Final Report of the Intergroup Randomized Study of Combined Androgen-Deprivation Therapy Plus Radiotherapy Versus Androgen-Deprivation Therapy Alone in Locally Advanced Prostate Cancer. ^51^ | 2015 | Genito-urinary | Primary or Co-Primary | Hazard ratio and p-value from an unadjusted Cox model | Yes | Yes | No | Yes | Adjusted Analysis | Yes - Adjusted for some or all stratification factors | Yes | Both Adjusted and Unadjusted Analysis | Yes - Adjusted for both strat and non-strat factors | No | N/A | N/A | N/A | N/A | N/A | N/A |
| 245 | Locoregional treatment versus no treatment of the primary tumour in metastatic breast cancer: an open-label randomised controlled trial. ^52^ | 2015 | Breast | Primary or Co-Primary | Hazard ratio and P-value from a logrank test | Yes | Yes | No | Yes | Not Specified | Unsure - Not Specified | Yes | Adjusted Analysis | Yes - Adjusted for some or all stratification factors | Yes | No | Yes | No | Yes | Randomisation / balance of subsequent treatment lines is given as a reason as to why subsequent treatment lines will not have affected the OS results. | No |
| 247 | Neoadjuvant plus adjuvant bevacizumab in early breast cancer (NSABP B-40 [NRG Oncology]): secondary outcomes of a phase 3, randomised controlled trial. ^53^ | 2015 | Breast | Exploratory | Hazard ratio from an adjusted Cox model and p-value from an stratified logrank test. | Yes | Yes | No | Yes | Adjusted Analysis | Yes - Adjusted for some or all stratification factors | Yes | Adjusted Analysis | Yes - Adjusted for some or all stratification factors | No | N/A | N/A | N/A | N/A | N/A | N/A |
| 248 | Neoadjuvant chemoradiotherapy plus surgery versus surgery alone for oesophageal or junctional cancer (CROSS): long-term results of a randomised controlled trial. ^54^ | 2015 | Upper GI/HPB | Primary or Co-Primary | Hazard ratio from an unadjusted cox model and p-value from a logrank test. | Yes | Yes | No | Yes | Unadjusted Analysis | No - Only Unadjusted analysis | Yes | Both Adjusted and Unadjusted Analysis | Yes - Adjusted for both strat and non-strat factors | No | N/A | N/A | N/A | N/A | N/A | N/A |
| 250 | Adjuvant lymph-node field radiotherapy versus observation only in patients with melanoma at high risk of further lymph-node field relapse after lymphadenectomy (ANZMTG 01.02/TROG 02.01): 6-year follow-up of a phase 3, randomised controlled trial. ^55^ | 2015 | Other | Secondary | Hazard ratio and P-value from a stratified logrank test. | Yes | Yes | No | Yes | Adjusted Analysis | Yes - Unclear on what adjusted for | Yes | Adjusted Analysis | Yes - Adjusted for some or all stratification factors | Yes | Yes | No | Yes | No | N/A | No |
| 254 | Afatinib versus erlotinib as second-line treatment of patients with advanced squamous cell carcinoma of the lung (LUX-Lung 8): an open-label randomised controlled phase 3 trial. ^56^ | 2015 | Lung | Secondary | Hazard ratio from an unadjusted cox model and p-value from an stratified logrank test. | Yes | Yes | No | Yes | Adjusted Analysis | Yes - Adjusted for some or all stratification factors | Yes | Unadjusted Analysis | No - Only Unadjusted analysis | Yes | Yes | No | Yes | No | N/A | No |
| 263 | High-dose radiotherapy with short-term or long-term androgen deprivation in localised prostate cancer (DART01/05 GICOR): a randomised, controlled, phase 3 trial. ^57^ | 2015 | Genito-urinary | Secondary | Hazard ratio from a Cox model and p-value from a logrank test | Yes | Yes | No | Yes | Unadjusted Analysis | No - Only Unadjusted analysis | Yes | Unadjusted Analysis | No - Only Unadjusted analysis | No | N/A | N/A | N/A | N/A | N/A | N/A |
| 267 | Breast-conserving surgery with or without irradiation in women aged 65 years or older with early breast cancer (PRIME II): a randomised controlled trial. ^58^ | 2015 | Breast | Secondary | Other (5 year OS rate and I think a p-value from a logrank test.) | No | N/A | N/A | Yes | Not Specified | Unsure - Not Specified | No | N/A | N/A | No | N/A | N/A | N/A | N/A | N/A | N/A |
| 269 | Whole-breast irradiation with or without a boost for patients treated with breast-conserving surgery for early breast cancer: 20-year follow-up of a randomised phase 3 trial. ^59^ | 2015 | Breast | Primary or Co-Primary | Hazard ratio from a Cox model and p-value from a logrank test | Yes | Yes | No | Yes | Not Specified | Unsure - Not Specified | Yes | Not Specified | Unsure - Not Specified | Yes | Yes | No | Yes | Yes | The uptake of subsequent treatments is given as a reason for reduced OS effect / stated as may have affected the results / used to caveat the results. | No |
| 275 | Use of thoracic radiotherapy for extensive stage small-cell lung cancer: a phase 3 randomised controlled trial. ^60^ | 2015 | Lung | Primary or Co-Primary | Other (Overall survival at 1 year  but their HR is clearly for  the entire follow-up  period.) | Yes | Yes | No | Yes | Not Specified | Unsure - Not Specified | No | N/A | N/A | No | N/A | N/A | N/A | N/A | N/A | N/A |
| 283 | Gemcitabine and capecitabine with or without telomerase peptide vaccine GV1001 in patients with locally advanced or metastatic pancreatic cancer (TeloVac): an open-label, randomised, phase 3 trial. ^61^ | 2014 | Upper GI/HPB | Primary or Co-Primary | Hazard ratio from an adjusted Cox model and p-value from a logrank test. | Yes | Yes | No | Yes | Adjusted Analysis | Yes - Adjusted for some or all stratification factors | Yes | Adjusted Analysis | Yes - Adjusted for some or all stratification factors | Yes | Yes | No | Yes | Yes | Subsequent treatments are stated to not have affected the OS results as an OS benefit was observed or OS was similar between those who did and did not receive a subsequent treatment. | Yes (Post-Hoc separating out the groups in an OS analysis.) |
| 285 | Augmented post-remission therapy for a minimal residual disease-defined high-risk subgroup of children and young people with clinical standard-risk and intermediate-risk acute lymphoblastic leukaemia (UKALL 2003): a randomised controlled trial. ^62^ | 2014 | Haematological | Primary or Co-Primary | Other (Odds Ratio with 95% CI and p-value calculated at 5 years.) | No | N/A | N/A | No | N/A | N/A | No | N/A | N/A | Yes | Yes | No | Yes | No | N/A | No |
| 286 | Adjuvant bevacizumab in patients with melanoma at high risk of recurrence (AVAST-M): preplanned interim results from a multicentre, open-label, randomised controlled phase 3 study. ^63^ | 2014 | Other | Primary or Co-Primary | Hazard ratio from a Cox model and p-value from a logrank test | Yes | Yes | No | Yes | Not Specified | Unsure - Not Specified | Yes | Both Adjusted and Unadjusted Analysis | Yes - Adjusted for both strat and non-strat factors | Yes | Yes | No | Yes | Yes | Other (Limited Follow-up) | No |
| 290 | Escalated-dose versus control-dose conformal radiotherapy for prostate cancer: long-term results from the MRC RT01 randomised controlled trial. ^64^ | 2014 | Genito-urinary | Primary or Co-Primary | Hazard ratio and p-value from an adjusted Cox model | Yes | Yes | No | No | N/A | N/A | Yes | Adjusted Analysis | Yes - Adjusted for some or all stratification factors | Yes | Yes | No | No | Yes | The lack of uptake of subsequent treatments is given as a reason for no OS effect / may have negatively affected OS. | No |
| 299 | Doxorubicin alone versus intensified doxorubicin plus ifosfamide for first-line treatment of advanced or metastatic soft-tissue sarcoma: a randomised controlled phase 3 trial. ^65^ | 2014 | Other | Primary or Co-Primary | Hazard ratio and P-value from a logrank test | Yes | Yes | No | Yes | Adjusted Analysis | Yes - Unclear on what adjusted for | No | N/A | N/A | Yes | Yes | No | Yes | Yes | The uptake of subsequent treatments is given as a reason for reduced OS effect / stated as may have affected the results / used to caveat the results. | No |
| 300 | Concomitant cisplatin plus radiotherapy and high-dose-rate brachytherapy versus radiotherapy alone for stage IIIB epidermoid cervical cancer: a randomized controlled trial. ^66^ | 2014 | Gynaecological | Secondary | Hazard ratio and p-value from an adjusted Cox model | Yes | Yes | No | No | N/A | N/A | Yes | Adjusted Analysis | Yes - Unclear on what adjusted for | No | N/A | N/A | N/A | N/A | N/A | N/A |
| 301 | Docetaxel plus nintedanib versus docetaxel plus placebo in patients with previously treated non-small-cell lung cancer (LUME-Lung 1): a phase 3, double-blind, randomised controlled trial. ^67^ | 2014 | Lung | Secondary | Hazard ratio from an unadjusted cox model and p-value from a logrank test. | Yes | Yes | No | Yes | Adjusted Analysis | Yes - Unclear on what adjusted for | Yes | Adjusted Analysis | Yes - Adjusted for some or all stratification factors | Yes | Yes | No | Yes | Yes | Randomisation / balance of subsequent treatment lines is given as a reason as to why subsequent treatment lines will not have affected the OS results. | No |
| 302 | Docetaxel versus active symptom control for refractory oesophagogastric adenocarcinoma (COUGAR-02): an open-label, phase 3 randomised controlled trial. ^68^ | 2014 | Upper GI/HPB | Primary or Co-Primary | Hazard Ratio and 95% CI from an unadjusted Cox model | Yes | Yes | No | No | N/A | N/A | Yes | Both Adjusted and Unadjusted Analysis | Yes - Adjusted for some or all stratification factors | Yes | Yes | No | Yes | Yes | The uptake of subsequent treatments is given as a reason for reduced OS effect / stated as may have affected the results / used to caveat the results. | No |
| 306 | Randomized, multicenter, phase II study of CO-101 versus gemcitabine in patients with metastatic pancreatic ductal adenocarcinoma: including a prospective evaluation of the role of hENT1 in gemcitabine or CO-101 sensitivity. ^69^ | 2013 | Upper GI/HPB | Primary or Co-Primary | Hazard ratio and P-value from a logrank test | Yes | Yes | Yes | Yes | Not Specified | Unsure - Not Specified | No | N/A | N/A | Yes | Yes | No | No | No | N/A | No |
| 312 | 2 years versus 1 year of adjuvant trastuzumab for HER2-positive breast cancer (HERA): an open-label, randomised controlled trial. ^70^ | 2013 | Breast | Secondary | Hazard ratio from an unadjusted cox model and p-value from a logrank test. | No | N/A | N/A | Yes | Not Specified | Unsure - Not Specified | Yes | Unadjusted Analysis | No - Only Unadjusted analysis | Yes | Yes | No | Yes | Yes | Subsequent treatments are stated to not have affected the OS results as an OS benefit was observed or OS was similar between those who did and did not receive a subsequent treatment. | Yes (Cut-off of follow-up time to see how the effect changed.) |
| 318 | Randomized controlled trial of toremifene 120 mg compared with exemestane 25 mg after prior treatment with a non-steroidal aromatase inhibitor in postmenopausal women with hormone receptor-positive metastatic breast cancer. ^71^ | 2013 | Breast | Secondary | Hazard ratio and P-value from a logrank test | Yes | Yes | No | Yes | Not Specified | Unsure - Not Specified | No | N/A | N/A | No | N/A | N/A | N/A | N/A | N/A | N/A |
| 319 | Chemoradiotherapy with or without cetuximab in patients with oesophageal cancer (SCOPE1): a multicentre, phase 2/3 randomised trial. ^72^ | 2013 | Upper GI/HPB | Secondary | Hazard ratio from an adjusted Cox model and p-value from a logrank test. | Yes | Yes | No | Yes | Unadjusted Analysis | No - Only Unadjusted analysis | Yes | Both Adjusted and Unadjusted Analysis | Yes - Adjusted for some or all stratification factors | Yes | Yes | No | Yes | No | N/A | No |
| 326 | Intraoperative radiotherapy versus external radiotherapy for early breast cancer (ELIOT): a randomised controlled equivalence trial. ^73^ | 2013 | Breast | Secondary | Hazard ratio from an unadjusted cox model and p-value from a logrank test. | Yes | Yes | No | Yes | Not Specified | Unsure - Not Specified | Yes | Unadjusted Analysis | No - Only Unadjusted analysis | Yes | Yes | No | Yes | No | N/A | No |
| 328 | Long-term results of dose-dense paclitaxel and carboplatin versus conventional paclitaxel and carboplatin for treatment of advanced epithelial ovarian, fallopian tube, or primary peritoneal cancer (JGOG 3016): a randomised, controlled, open-label trial. ^74^ | 2013 | Gynaecological | Secondary | Hazard ratio from an adjusted Cox model and p-value from a logrank test. | Yes | Yes | No | Yes | Adjusted Analysis | Yes - Adjusted for some or all stratification factors | Yes | Adjusted Analysis | Yes - Adjusted for some or all stratification factors | Yes | No | No | No | Yes | The uptake of subsequent treatments is given as a reason for reduced OS effect / stated as may have affected the results / used to caveat the results. | No |
| 329 | Erlotinib versus docetaxel as second-line treatment of patients with advanced non-small-cell lung cancer and wild-type EGFR tumours (TAILOR): a randomised controlled trial. ^75^ | 2013 | Lung | Primary or Co-Primary | Hazard ratio and p-value from an adjusted Cox model | Yes | Yes | No | No | N/A | N/A | Yes | Both Adjusted and Unadjusted Analysis | Yes - Adjusted for both strat and non-strat factors | Yes | Yes | No | Yes | No | N/A | No |
| 333 | Randomized controlled trial of cetuximab plus chemotherapy for patients with KRAS wild-type unresectable colorectal liver-limited metastases. ^76^ | 2013 | Bowel / Lower GI | Secondary | Hazard ratio and P-value from a logrank test | Yes | Yes | No | Yes | Adjusted Analysis | Yes - Unclear on what adjusted for | No | N/A | N/A | No | N/A | N/A | N/A | N/A | N/A | N/A |
| 335 | Taking into account successive treatment lines in the analysis of a colorectal cancer randomised trial. ^77^ | 2013 | Bowel / Lower GI | Primary or Co-Primary | Hazard ratio and p-value from an adjusted Cox model | No | N/A | N/A | No | N/A | N/A | Yes | Adjusted Analysis | Yes - Adjusted for some or all stratification factors | Yes | Yes | Yes | Yes | Yes | Other (Sequence) | Yes (Shared Frailty Models and Time Dependent Covariates.) |
| 337 | Radiofrequency ablation with or without transcatheter arterial chemoembolization in the treatment of hepatocellular carcinoma: a prospective randomized trial. ^78^ | 2013 | Upper GI/HPB | Primary or Co-Primary | Hazard ratio and p-value from an adjusted Cox model | Yes | Yes | No | No | N/A | N/A | Yes | Adjusted Analysis | Yes - Adjusted for both strat and non-strat factors | Yes | Yes | No | Yes | No | N/A | No |
| 344 | Vinorelbine and gemcitabine vs vinorelbine and carboplatin as first-line treatment of advanced NSCLC. A phase III randomised controlled trial by the Norwegian Lung Cancer Study Group. ^79^ | 2012 | Lung | Primary or Co-Primary | Hazard ratio and P-value from a logrank test | Yes | Yes | No | Yes | Not Specified | Unsure - Not Specified | Yes | Not Specified | Unsure - Not Specified | Yes | Yes | No | Yes | Yes | The lack of uptake of subsequent treatments is given as a reason for no OS effect / may have negatively affected OS. | No |
| 345 | Celecoxib plus hormone therapy versus hormone therapy alone for hormone-sensitive prostate cancer: first results from the STAMPEDE multiarm, multistage, randomised controlled trial. ^80^ | 2012 | Genito-urinary | Primary or Co-Primary | N/A | N/A | N/A | N/A | N/A | N/A | N/A | N/A | N/A | N/A | Yes | N/A | N/A | N/A | Yes | The uptake of subsequent treatments is given as a reason for better OS results or a reduced event rate than expected. | N/A |
| 349 | Bevacizumab plus oxaliplatin-based chemotherapy as adjuvant treatment for colon cancer (AVANT): a phase 3 randomised controlled trial. ^81^ | 2012 | Bowel / Lower GI | Secondary | Hazard ratio from a Cox model and p-value from a logrank test | Yes | Yes | No | Unsure | N/A | N/A | Unsure | N/A | N/A | No | N/A | N/A | N/A | N/A | N/A | N/A |
| 351 | Adjuvant chemotherapy with doxorubicin, ifosfamide, and lenograstim for resected soft-tissue sarcoma (EORTC 62931): a multicentre randomised controlled trial. ^82^ | 2012 | Other | Primary or Co-Primary | Hazard ratio and P-value from a logrank test | Yes | Yes | No | Yes | Not Specified | Unsure - Not Specified | No | N/A | N/A | No | N/A | N/A | N/A | N/A | N/A | N/A |
| 354 | Adjuvant radiotherapy versus observation alone for patients at risk of lymph-node field relapse after therapeutic lymphadenectomy for melanoma: a randomised trial. ^83^ | 2012 | Other | Secondary | Hazard ratio and P-value from a logrank test | Yes | Yes | No | Yes | Both Adjusted and Unadjusted Analysis | Yes - Unclear on what adjusted for | Yes | Adjusted Analysis | Yes - Adjusted for some or all stratification factors | No | N/A | N/A | N/A | N/A | N/A | N/A |
| 358 | Adjuvant capecitabine and oxaliplatin for gastric cancer after D2 gastrectomy (CLASSIC): a phase 3 open-label, randomised controlled trial. ^84^ | 2012 | Upper GI/HPB | Secondary | Hazard ratio from a Cox model and p-value from a logrank test | Yes | Yes | No | Yes | Not Specified | Unsure - Not Specified | Yes | Not Specified | Unsure - Not Specified | No | N/A | N/A | N/A | N/A | N/A | N/A |
| 366 | Intermittent versus continuous oxaliplatin and fluoropyrimidine combination chemotherapy for first-line treatment of advanced colorectal cancer: results of the randomised phase 3 MRC COIN trial. ^85^ | 2011 | Bowel / Lower GI | Primary or Co-Primary | Other (Upper bound of the confidence interval from an 80% logrank test.) | Yes | Yes | No | Yes | Not Specified | Unsure - Not Specified | No | N/A | N/A | Yes | Yes | No | No | Yes | The lack of uptake of subsequent treatments is given as a reason for no OS effect / may have negatively affected OS. | No |
| 367 | Addition of cetuximab to oxaliplatin-based first-line combination chemotherapy for treatment of advanced colorectal cancer: results of the randomised phase 3 MRC COIN trial. ^86^ | 2011 | Bowel / Lower GI | Primary or Co-Primary | Hazard ratio and P-value from a logrank test | Yes | Yes | No | Yes | Not Specified | Unsure - Not Specified | No | N/A | N/A | Yes | Yes | No | Yes | Yes | The lack of uptake of subsequent treatments is given as a reason for no OS effect / may have negatively affected OS. | No |
| 373 | First-line treatment with zoledronic acid as compared with clodronic acid in multiple myeloma (MRC Myeloma IX): a randomised controlled trial. ^87^ | 2010 | Haematological | Primary or Co-Primary | Hazard ratio and p-value from an adjusted Cox model | Yes | Yes | No | Yes | Adjusted Analysis | Yes - Adjusted for non-stratification factors | Yes | Adjusted Analysis | Yes - Adjusted for both strat and non-strat factors | No | N/A | N/A | N/A | N/A | N/A | N/A |
| 374 | Effect of anastrozole and tamoxifen as adjuvant treatment for early-stage breast cancer: 10-year analysis of the ATAC trial. ^88^ | 2010 | Breast | Secondary | Hazard ratio and p-value from an unadjusted Cox model | No | N/A | N/A | No | N/A | N/A | Yes | Unadjusted Analysis | No - Only Unadjusted analysis | No | N/A | N/A | N/A | N/A | N/A | N/A |
| 376 | Early versus delayed treatment of relapsed ovarian cancer (MRC OV05/EORTC 55955): a randomised trial. ^89^ | 2010 | Gynaecological | Primary or Co-Primary | Hazard ratio and P-value from a stratified logrank test. | Yes | Yes | No | Yes | Adjusted Analysis | Yes - Adjusted for non-stratification factors | Yes | Adjusted Analysis | Yes - Adjusted for both strat and non-strat factors | Yes | Yes | No | No | Yes | Other (Early Access) | No |
| 379 | Neo-adjuvant chemotherapy alone or with regional hyperthermia for localised high-risk soft-tissue sarcoma: a randomised phase 3 multicentre study. ^90^ | 2010 | Other | Secondary | Hazard ratio and P-value from a stratified logrank test. | Yes | Yes | No | Yes | Adjusted Analysis | Yes - Unclear on what adjusted for | No | N/A | N/A | No | N/A | N/A | N/A | N/A | N/A | N/A |
| 381 | Randomized trial comparing conventional-dose with high-dose conformal radiation therapy in early-stage adenocarcinoma of the prostate: long-term results from proton radiation oncology group/american college of radiology 95-09. ^91^ | 2010 | Genito-urinary | Secondary | Other  (Proportions and a logrank p-value) | No | N/A | N/A | Yes | Not Specified | Unsure - Not Specified | No | N/A | N/A | No | N/A | N/A | N/A | N/A | N/A | N/A |
| 382 | Overall survival analysis of a phase II randomized controlled trial of a Poxviral-based PSA-targeted immunotherapy in metastatic castration-resistant prostate cancer. ^92^ | 2010 | Genito-urinary | Secondary | Hazard ratio from an adjusted Cox model and p-value from an stratified logrank test. | Yes | No | N/A | Yes | Adjusted Analysis | Yes - Adjusted for some or all stratification factors | Yes | Adjusted Analysis | Yes - Adjusted for some or all stratification factors | Yes | No | No | No | Yes | None or limited options of subsequent treatment lines for patients are given as a reason as to why subsequent treatment lines will not have affected the OS results. | No |
| 384 | Lenalidomide plus high-dose dexamethasone versus lenalidomide plus low-dose dexamethasone as initial therapy for newly diagnosed multiple myeloma: an open-label randomised controlled trial. ^93^ | 2010 | Haematological | Exploratory | Other (OS at one year and p-value from logrank test.) | Yes | Yes | No | No | N/A | N/A | Yes | Both Adjusted and Unadjusted Analysis | Yes - Adjusted for some or all stratification factors | Yes | No | No | No | Yes | The uptake of subsequent treatments is given as a reason for reduced OS effect / stated as may have affected the results / used to caveat the results. | No |
| 385 | Autologous stem cell transplantation as a first-line treatment strategy for chronic lymphocytic leukemia: a multicenter, randomized, controlled trial from the SFGM-TC and GFLLC. ^94^ | 2011 | Haematological | Secondary | Other (% of OS at 3 and 5 years) | Yes | No | N/A | No | N/A | N/A | Yes | Adjusted Analysis | Yes - Adjusted for some or all stratification factors | No | N/A | N/A | N/A | N/A | N/A | N/A |
| 390 | Single-injection depot progesterone before surgery and survival in women with operable breast cancer: a randomized controlled trial. ^95^ | 2011 | Breast | Secondary | Hazard ratio and P-value from a logrank test | Yes | Yes | No | Yes | Not Specified | Unsure - Not Specified | Yes | Adjusted Analysis | Yes - Adjusted for both strat and non-strat factors | No | N/A | N/A | N/A | N/A | N/A | N/A |
| 395 | Treatment with trastuzumab for 1 year after adjuvant chemotherapy in patients with HER2-positive early breast cancer: a 4-year follow-up of a randomised controlled trial. ^96^ | 2011 |  | Secondary | Hazard ratio and p-value from an unadjusted Cox model | Yes | Yes | No | No | N/A | N/A | Yes | Unadjusted Analysis | No - Only Unadjusted analysis | Yes | Yes | No | Yes | Yes | The uptake of subsequent treatments is given as a reason for the discrepancy between OS and surrogate endpoints. | Yes (Censored at point of cross-over.) |
| 404 | Best supportive care compared with chemotherapy for unresectable gall bladder cancer: a randomized controlled study. ^97^ | 2010 |  | Primary or Co-Primary | Hazard ratio from a Cox model and p-value from a logrank test | Yes | No | N/A | Yes | Not Specified | Unsure - Not Specified | Yes | Not Specified | Unsure - Not Specified | No | N/A | N/A | N/A | N/A | N/A | N/A |
| 405 | Trastuzumab in combination with chemotherapy versus chemotherapy alone for treatment of HER2-positive advanced gastric or gastro-oesophageal junction cancer (ToGA): a phase 3, open-label, randomised controlled trial. ^98^ | 2010 |  | Primary or Co-Primary | Hazard ratio and P-value from a logrank test | Yes | Yes | No | Yes | Adjusted Analysis | Yes - Adjusted for some or all stratification factors | Yes | Unadjusted Analysis | No - Only Unadjusted analysis | Yes | Yes | No | Yes | Yes | The uptake of subsequent treatments is given as a reason for better OS results or a reduced event rate than expected. | No |

Supplementary Table 4: Summary of the clinical effectiveness papers by year of publication

|  | **2010 (n=9)** | **2011 (n=5)** | **2012 (n=6)** | **2013 (n=10)** | **2014 (n=8)** | **2015 (n=13)** | **2016 (n=9)** | **2017 (n=10)** | **2018 (n=5)** | **2019 (n=8)** | **2020 (n=7)** | **2021 (n=8)** | **Total (n=98)** |
| --- | --- | --- | --- | --- | --- | --- | --- | --- | --- | --- | --- | --- | --- |
| **OS Endpoint** |  |  |  |  |  |  |  |  |  |  |  |  |  |
| Primary or Co-Primary | 4  (44.4%) | 2 (40.0%) | 3 (50.0%) | 4 (40.0%) | 6 (75.0%) | 6 (46.2%) | 5 (55.6%) | 7 (70.0%) | 1 (20.0%) | 3 (37.5%) | 1 (14.3%) | 3 (37.5%) | 45  (45.9%) |
| Secondary | 4 (44.4%) | 3 (60.0%) | 3 (50.0%) | 6 (60.0%) | 2 (25.0%) | 6 (46.2%) | 3 (33.3%) | 2 (20.0%) | 4 (80.0%) | 5 (62.5%) | 5 (71.4%) | 5 (62.5%) | 48  (49.0%) |
| Exploratory | 1 (11.1%) | 0  (0.0%) | 0  (0.0%) | 0  (0.0%) | 0  (0.0%) | 1  (7.7%) | 1 (11.1%) | 1 (10.0%) | 0  (0.0%) | 0  (0.0%) | 1 (14.3%) | 0  (0.0%) | 5  (5.1%) |
| **Was a Kaplan-Meier curve for OS presented in the main text?** | | | | | | | | | | | | | |
| Yes | 7 (77.8%) | 5 (100.0%) | 5 (83.3%) | 8 (80.0%) | 7 (87.5%) | 12 (92.3%) | 7 (77.8%) | 10 (100.0%) | 5 (100.0%) | 6 (75.0%) | 7 (100.0%) | 7 (87.5%) | 86 (87.8%) |
| No | 2 (22.2%) | 0  (0.0%) | 0  (0.0%) | 2 (20.0%) | 1 (12.5%) | 1  (7.7%) | 1 (11.1%) | 0  (0.0%) | 0  (0.0%) | 0  (0.0%) | 0  (0.0%) | 1 (12.5%) | 8 (8.2%) |
| N/A^1^ | 0  (0.0%) | 0  (0.0%) | 1 (16.7%) | 0  (0.0%) | 0  (0.0%) | 0  (0.0%) | 1 (11.1%) | 0  (0.0%) | 0  (0.0%) | 2 (25.0%) | 0  (0.0%) | 0  (0.0%) | 4 (4.1%) |
| **Were the numbers at risk included in the Kaplan-Meier curve?** | | | | | | | | | | | | | |
| Yes | 5 (55.6%) | 4 (80.0%) | 5 (83.3%) | 8 (80.0%) | 7 (87.5%) | 12 (92.3%) | 7 (77.8%) | 7 (70.0%) | 5 (100.0%) | 6 (75.0%) | 6 (85.7%) | 7 (87.5%) | 79 (80.6%) |
| No | 2 (22.2%) | 1 (20.0%) | 0  (0.0%) | 0  (0.0%) | 0  (0.0%) | 0  (0.0%) | 0  (0.0%) | 3 (30.0%) | 0  (0.0%) | 0  (0.0%) | 1 (14.3%) | 0  (0.0%) | 7 (7.1%) |
| N/A | 2 (22.2%) | 0  (0.0%) | 1 (16.7%) | 2 (20.0%) | 1 (12.5%) | 1 (7.7%) | 2 (22.2%) | 0  (0.0%) | 0  (0.0%) | 2 (25.0%) | 0  (0.0%) | 1 (12.5%) | 12 (12.2%) |
| **Were the number of patients censored or number of events included in the at risk table** | | | | | | | | | | | | | |
| Yes | 0  (0.0%) | 0  (0.0%) | 0  (0.0%) | 1 (10.0%) | 0  (0.0%) | 1  (7.7%) | 2 (22.2%) | 3 (30.0%) | 0  (0.0%) | 2 (25.0%) | 2 (28.6%) | 2 (25.0%) | 13 (13.3%) |
| No | 5 (55.6%) | 4 (80.0%) | 5 (83.3%) | 7 (70.0%) | 7 (87.5%) | 11 (84.6%) | 5 (55.6%) | 4 (40.0%) | 5 (100.0%) | 4 (50.0%) | 4 (57.1%) | 5 (62.5%) | 66 (67.3%) |
| N/A | 4 (44.4%) | 1 (20.0%) | 1 (16.7%) | 2 (20.0%) | 1 (12.5%) | 1  (7.7%) | 2 (22.2%) | 3 (30.0%) | 0  (0.0%) | 2 (25.0%) | 1 (14.3%) | 1 (12.5%) | 19 (19.4%) |
| **Were confidence intervals included in the Kaplan-Meier curve?** | | | | | | | | | | | | | |
| No | 7 (77.8%) | 5 (100.0%) | 5 (83.3%) | 8 (80.0%) | 7 (87.5%) | 12 (92.3%) | 7 (77.8%) | 10 (100.0%) | 5 (100.0%) | 6 (75.0%) | 7 (100.0%) | 7 (87.5%) | 86 (87.8%) |
| N/A | 2 (22.2%) | 0  (0.0%) | 1 (16.7%) | 2 (20.0%) | 1 (12.5%) | 1  (7.7%) | 2 (22.2%) | 0  (0.0%) | 0  (0.0%) | 2 (25.0%) | 0  (0.0%) | 1 (12.5%) | 12 (12.2%) |
| **Primary analysis** |  |  |  |  |  |  |  |  |  |  |  |  |  |
| Hazard Ratio and 95% CI from an unadjusted Cox model | 0  (0.0%) | 0  (0.0%) | 0  (0.0%) | 0  (0.0%) | 1 (12.5%) | 0  (0.0%) | 0  (0.0%) | 0  (0.0%) | 0  (0.0%) | 0  (0.0%) | 0  (0.0%) | 0  (0.0%) | 1 (1.0%) |
| Hazard ratio and 95% CI from an adjusted Cox model | 0  (0.0%) | 0  (0.0%) | 0  (0.0%) | 0  (0.0%) | 0  (0.0%) | 1  (7.7%) | 0  (0.0%) | 0  (0.0%) | 0  (0.0%) | 0  (0.0%) | 0  (0.0%) | 1 (12.5%) | 2 (2.0%) |
| Hazard ratio and P-value from a logrank test | 1 (11.1%) | 2 (40.0%) | 3 (50.0%) | 3 (30.0%) | 1 (12.5%) | 1  (7.7%) | 0  (0.0%) | 1 (10.0%) | 4 (80.0%) | 1 (12.5%) | 1 (14.3%) | 1 (12.5%) | 19 (19.4%) |
| Hazard ratio and P-value from a stratified logrank test. | 2 (22.2%) | 0  (0.0%) | 0  (0.0%) | 0  (0.0%) | 0  (0.0%) | 1  (7.7%) | 1 (11.1%) | 1 (10.0%) | 0  (0.0%) | 1 (12.5%) | 0  (0.0%) | 0  (0.0%) | 6 (6.1%) |
| Hazard ratio and p-value from an adjusted Cox model | 1 (11.1%) | 0  (0.0%) | 0  (0.0%) | 3 (30.0%) | 2 (25.0%) | 0  (0.0%) | 2 (22.2%) | 3 (30.0%) | 1 (20.0%) | 1 (12.5%) | 0  (0.0%) | 2 (25.0%) | 15 (15.3%) |
| Hazard ratio and p-value from an unadjusted Cox model | 1 (11.1%) | 1 (20.0%) | 0  (0.0%) | 0  (0.0%) | 0  (0.0%) | 1  (7.7%) | 0  (0.0%) | 2 (20.0%) | 0  (0.0%) | 0  (0.0%) | 0  (0.0%) | 0  (0.0%) | 5 (5.1%) |
| Hazard ratio from a Cox model and p-value from a logrank test | 1 (11.1%) | 0  (0.0%) | 2 (33.3%) | 0  (0.0%) | 1 (12.5%) | 2 (15.4%) | 0  (0.0%) | 0  (0.0%) | 0  (0.0%) | 1 (12.5%) | 2 (28.6%) | 1 (12.5%) | 10 (10.2%) |
| Hazard ratio from an adjusted Cox model and p-value from a logrank test. | 0  (0.0%) | 0  (0.0%) | 0  (0.0%) | 2 (20.0%) | 1 (12.5%) | 1  (7.7%) | 1 (11.1%) | 1 (10.0%) | 0  (0.0%) | 0  (0.0%) | 0  (0.0%) | 0  (0.0%) | 6 (6.1%) |
| Hazard ratio from an adjusted Cox model and p-value from an stratified logrank test. | 1 (11.1%) | 0  (0.0%) | 0  (0.0%) | 0  (0.0%) | 0  (0.0%) | 1  (7.7%) | 4 (44.4%) | 1 (10.0%) | 0  (0.0%) | 1 (12.5%) | 3 (42.9%) | 2 (25.0%) | 13 (13.3%) |
| Hazard ratio from an unadjusted cox model and p-value from a logrank test. | 0  (0.0%) | 0  (0.0%) | 0  (0.0%) | 2 (20.0%) | 1 (12.5%) | 1  (7.7%) | 0  (0.0%) | 0  (0.0%) | 0  (0.0%) | 1 (12.5%) | 0  (0.0%) | 0  (0.0%) | 5 (5.1%) |
| Hazard ratio from an unadjusted cox model and p-value from an stratified logrank test. | 0  (0.0%) | 0  (0.0%) | 0  (0.0%) | 0  (0.0%) | 0  (0.0%) | 1  (7.7%) | 0  (0.0%) | 0  (0.0%) | 0  (0.0%) | 0  (0.0%) | 1 (14.3%) | 0  (0.0%) | 2 (2.0%) |
| N/A^1^ | 0  (0.0%) | 0  (0.0%) | 1 (16.7%) | 0  (0.0%) | 0  (0.0%) | 0  (0.0%) | 1 (11.1%) | 0  (0.0%) | 0  (0.0%) | 2 (25.0%) | 0  (0.0%) | 0  (0.0%) | 4 (4.1%) |
| Other | 2 (22.2%) | 2 (40.0%) | 0  (0.0%) | 0  (0.0%) | 1 (12.5%) | 3 (23.1%) | 0  (0.0%) | 1 (10.0%) | 0  (0.0%) | 0  (0.0%) | 0  (0.0%) | 1 (12.5%) | 10 (10.2%) |
| **Were subsequent treatments mentioned in the paper?** | | | | | | | | | | | | | |
| Yes | 4 (44.4%) | 3 (60.0%) | 2 (33.3%) | 8 (80.0%) | 7 (87.5%) | 5 (38.5%) | 7 (77.8%) | 6 (60.0%) | 4 (80.0%) | 5 (62.5%) | 3 (42.9%) | 5 (62.5%) | 59 (60.2%) |
| No | 5 (55.6%) | 2 (40.0%) | 4 (66.7%) | 2 (20.0%) | 1 (12.5%) | 8 (61.5%) | 2 (22.2%) | 4 (40.0%) | 1 (20.0%) | 3 (37.5%) | 4 (57.1%) | 3 (37.5%) | 39 (39.8%) |
| **Was the percentage or number of participants who received later lines included in the main text or appendix?** | | | | | | | | | | | | | |
| Yes | 2 (22.2%) | 3 (60.0%) | 1 (16.7%) | 7 (70.0%) | 7 (87.5%) | 3 (23.1%) | 7 (77.8%) | 5 (50.0%) | 3 (60.0%) | 5 (62.5%) | 2 (28.6%) | 5 (62.5%) | 50 (51.0%) |
| No | 2 (22.2%) | 0  (0.0%) | 0  (0.0%) | 1 (10.0%) | 0  (0.0%) | 2 (15.4%) | 0  (0.0%) | 1 (10.0%) | 1 (20.0%) | 0  (0.0%) | 1 (14.3%) | 0  (0.0%) | 8 (8.2%) |
| N/A | 5 (55.6%) | 2 (40.0%) | 5 (83.3%) | 2 (20.0%) | 1 (12.5%) | 8 (61.5%) | 2 (22.2%) | 4 (40.0%) | 1 (20.0%) | 3 (37.5%) | 4 (57.1%) | 3 (37.5%) | 40 (40.8%) |
| **Was a breakdown or summary of the number of subsequent treatments (Two, Three, Four) included in the main text or appendix?** | | | | | | | | | | | | | |
| Yes | 0  (0.0%) | 0  (0.0%) | 0  (0.0%) | 1 (10.0%) | 0  (0.0%) | 1  (7.7%) | 0  (0.0%) | 0  (0.0%) | 0  (0.0%) | 0  (0.0%) | 1 (14.3%) | 1 (12.5%) | 4 (4.1%) |
| No | 4 (44.4%) | 3 (60.0%) | 1 (16.7%) | 7 (70.0%) | 7 (87.5%) | 4 (30.8%) | 7 (77.8%) | 5 (50.0%) | 4 (80.0%) | 5 (62.5%) | 2 (28.6%) | 4 (50.0%) | 53 (54.1%) |
| N/A^2^ | 5 (55.6%) | 2 (40.0%) | 5 (83.3%) | 2 (20.0%) | 1 (12.5%) | 8 (61.5%) | 2 (22.2%) | 5 (50.0%) | 1 (20.0%) | 3 (37.5%) | 4 (57.1%) | 3 (37.5%) | 41 (41.8%) |
| **Was a breakdown or summary of the type of subsequent treatments (Treatment 1, Treatment 2, Treatment 3) included in the main text or appendix?** | | | | | | | | | | | | | |
| Yes | 1 (11.1%) | 2 (40.0%) | 1 (16.7%) | 6 (60.0%) | 6 (75.0%) | 3 (23.1%) | 7 (77.8%) | 4 (40.0%) | 3 (60.0%) | 5 (62.5%) | 2 (28.6%) | 5 (62.5%) | 45 (45.9%) |
| No | 3 (33.3%) | 1 (20.0%) | 0  (0.0%) | 2 (20.0%) | 1 (12.5%) | 2 (15.4%) | 0  (0.0%) | 1 (10.0%) | 1 (20.0%) | 0  (0.0%) | 1 (14.3%) | 0  (0.0%) | 12 (12.2%) |
| N/A^2^ | 5 (55.6%) | 2 (40.0%) | 5 (83.3%) | 2 (20.0%) | 1 (12.5%) | 8 (61.5%) | 2 (22.2%) | 5 (50.0%) | 1 (20.0%) | 3 (37.5%) | 4 (57.1%) | 3 (37.5%) | 41 (41.8%) |
| **Was additional analysis conducted to account for subsequent treatment lines?** | | | | | | | | | | | | | |
| Yes | 0  (0.0%) | 1 (20.0%) | 0  (0.0%) | 2 (20.0%) | 1 (12.5%) | 0  (0.0%) | 1 (11.1%) | 2 (20.0%) | 0  (0.0%) | 0  (0.0%) | 0  (0.0%) | 0  (0.0%) | 7 (7.1%) |
| No | 4 (44.4%) | 2 (40.0%) | 1 (16.7%) | 6 (60.0%) | 6 (75.0%) | 5 (38.5%) | 6 (66.7%) | 3 (30.0%) | 4 (80.0%) | 5 (62.5%) | 3 (42.9%) | 5 (62.5%) | 50 (51.0%) |
| N/A^2^ | 5 (55.6%) | 2 (40.0%) | 5 (83.3%) | 2 (20.0%) | 1 (12.5%) | 8 (61.5%) | 2 (22.2%) | 5 (50.0%) | 1 (20.0%) | 3 (37.5%) | 4 (57.1%) | 3 (37.5%) | 41 (41.8%) |
| **Were subsequent treatment lines mentioned in the discussion?** | | | | | | | | | | | | | |
| Yes | 4 (44.4%) | 3 (60.0%) | 2 (33.3%) | 3 (30.0%) | 6 (75.0%) | 3 (23.1%) | 6 (66.7%) | 2 (20.0%) | 1 (20.0%) | 1 (12.5%) | 3 (42.9%) | 5 (62.5%) | 39 (39.8%) |
| No | 0  (0.0%) | 0  (0.0%) | 0  (0.0%) | 5 (50.0%) | 1 (12.5%) | 2 (15.4%) | 1 (11.1%) | 4 (40.0%) | 3 (60.0%) | 4 (50.0%) | 0  (0.0%) | 0  (0.0%) | 20 (20.4%) |
| N/A | 5 (55.6%) | 2 (40.0%) | 4 (66.7%) | 2 (20.0%) | 1 (12.5%) | 8 (61.5%) | 2 (22.2%) | 4 (40.0%) | 1 (20.0%) | 3 (37.5%) | 4 (57.1%) | 3 (37.5%) | 39 (39.8%) |
| **How were subsequent treatments mentioned in the discussion?** | | | | | | | | | | | | | |
| The uptake of subsequent treatments is given as a reason for the discrepancy between OS and surrogate endpoints. | 0  (0.0%) | 1 (20.0%) | 0  (0.0%) | 0  (0.0%) | 0  (0.0%) | 0  (0.0%) | 0  (0.0%) | 0  (0.0%) | 0  (0.0%) | 0  (0.0%) | 0  (0.0%) | 1 (12.5%) | 2 (2.0%) |
| The uptake of subsequent treatments is given as a reason for better OS results or a reduced event rate than expected. | 1 (11.1%) | 0  (0.0%) | 1 (16.7%) | 0  (0.0%) | 0  (0.0%) | 0  (0.0%) | 3 (33.3%) | 0  (0.0%) | 1 (20.0%) | 0  (0.0%) | 0  (0.0%) | 0  (0.0%) | 6 (6.1%) |
| The uptake of subsequent treatments is given as a reason for reduced OS effect / stated as may have affected the results / used to caveat the results. | 1 (11.1%) | 0  (0.0%) | 0  (0.0%) | 1 (10.0%) | 2 (25.0%) | 1  (7.7%) | 1 (11.1%) | 2 (20.0%) | 0  (0.0%) | 1 (12.5%) | 2 (28.6%) | 3 (37.5%) | 14 (14.3%) |
| The lack of uptake of subsequent treatments is given as a reason for no OS effect / may have negatively affected OS. | 0  (0.0%) | 2 (40.0%) | 1 (16.7%) | 0  (0.0%) | 1 (12.5%) | 0  (0.0%) | 0  (0.0%) | 0  (0.0%) | 0  (0.0%) | 0  (0.0%) | 0  (0.0%) | 0  (0.0%) | 4 (4.1%) |
| Subsequent treatments are stated to not have affected the OS results as an OS benefit was observed or OS was similar between those who did and did not receive a subsequent treatment. | 0  (0.0%) | 0  (0.0%) | 0  (0.0%) | 1 (10.0%) | 1 (12.5%) | 0  (0.0%) | 1 (11.1%) | 0  (0.0%) | 0  (0.0%) | 0  (0.0%) | 0  (0.0%) | 0  (0.0%) | 3 (3.1%) |
| Randomisation / balance of subsequent treatment lines is given as a reason as to why subsequent treatment lines will not have affected the OS results. | 0  (0.0%) | 0  (0.0%) | 0  (0.0%) | 0  (0.0%) | 1 (12.5%) | 2 (15.4%) | 0  (0.0%) | 0  (0.0%) | 0  (0.0%) | 0  (0.0%) | 0  (0.0%) | 1 (12.5%) | 4 (4.1%) |
| None or limited options of subsequent treatment lines for patients are given as a reason as to why subsequent treatment lines will not have affected the OS results. | 1 (11.1%) | 0  (0.0%) | 0  (0.0%) | 0  (0.0%) | 0  (0.0%) | 0  (0.0%) | 0  (0.0%) | 0  (0.0%) | 0  (0.0%) | 0  (0.0%) | 1 (14.3%) | 0  (0.0%) | 2 (2.0%) |
| Other | 1 (11.1%) | 0  (0.0%) | 0  (0.0%) | 1 (10.0%) | 1 (12.5%) | 0  (0.0%) | 1 (11.1%) | 0  (0.0%) | 0  (0.0%) | 0  (0.0%) | 0  (0.0%) | 0  (0.0%) | 4 (4.1%) |
| N/A | 5 (55.6%) | 2 (40.0%) | 4 (66.7%) | 7 (70.0%) | 2 (25.0%) | 10 (76.9%) | 3 (33.3%) | 8 (80.0%) | 4 (80.0%) | 7 (87.5%) | 4 (57.1%) | 3 (37.5%) | 59 (60.2%) |

^1^ Not-Applicable here refers to papers where OS is a pre-specified endpoint, but the data is not mature at the time of the publication.

^2^ Note one paper stated no patients received subsequent treatments so is classed as N/A for these summaries.

N/A: Not Applicable; OS: Overall survival.

Supplementary Table 5: Summary of the Effectiveness papers by cancer

|  | **Bowel Lower GI (n=12)** | **Breast (n=12)** | **Genito-urinary (n=15)** | **Gynaecological (n=10)** | **Haematological (n=7)** | **Lung (n=18)** | **Upper GI HPB (n=15)** | **Other (n=9)** | **Total**  **(n=98)** |
| --- | --- | --- | --- | --- | --- | --- | --- | --- | --- |
| **OS Endpoint** |  |  |  |  |  |  |  |  |  |
| Primary or Co-Primary | 4 (33.3%) | 2 (16.7%) | 7 (46.7%) | 4 (40.0%) | 2 (28.6%) | 10 (55.6%) | 13 (86.7%) | 3 (33.3%) | 45 (45.9%) |
| Secondary | 8 (66.7%) | 7 (58.3%) | 8 (53.3%) | 6 (60.0%) | 4 (57.1%) | 8 (44.4%) | 2 (13.3%) | 5 (55.6%) | 48 (49.0%) |
| Exploratory | 0 (0.0%) | 3 (25.0%) | 0 (0.0%) | 0 (0.0%) | 1 (14.3%) | 0 (0.0%) | 0 (0.0%) | 1 (11.1%) | 5 (5.1%) |
| **Was a Kaplan-Meier curve for OS presented in the main text?** | | | | | | | | | |
| Yes | 11 (91.7%) | 9 (75.0%) | 12 (80.0%) | 7 (70.0%) | 6 (85.7%) | 17 (94.4%) | 15 (100.0%) | 9 (100.0%) | 86 (87.8%) |
| No | 1 (8.3%) | 3 (25.0%) | 2 (13.3%) | 1 (10.0%) | 1 (14.3%) | 0 (0.0%) | 0 (0.0%) | 0 (0.0%) | 8 (8.2%) |
| N/A^1^ | 0 (0.0%) | 0 (0.0%) | 1 (6.7%) | 2 (20.0%) | 0 (0.0%) | 1 (5.6%) | 0 (0.0%) | 0 (0.0%) | 4 (4.1%) |
| **Were the numbers at risk included in the Kaplan-Meier curve?** | | | | | | | | | |
| Yes | 10 (83.3%) | 9 (75.0%) | 11 (73.3%) | 6 (60.0%) | 5 (71.4%) | 16 (88.9%) | 13 (86.7%) | 9 (100.0%) | 79 (80.6%) |
| No | 1 (8.3%) | 0 (0.0%) | 1 (6.7%) | 1 (10.0%) | 1 (14.3%) | 1 (5.6%) | 2 (13.3%) | 0 (0.0%) | 7 (7.1%) |
| N/A | 1 (8.3%) | 3 (25.0%) | 3 (20.0%) | 3 (30.0%) | 1 (14.3%) | 1 (5.6%) | 0 (0.0%) | 0 (0.0%) | 12 (12.2%) |
| **Were the number of patients censored or number of events included in the at risk table** | | | | | | | | | |
| Yes | 1 (8.3%) | 0 (0.0%) | 3 (20.0%) | 0 (0.0%) | 2 (28.6%) | 1 (5.6%) | 4 (26.7%) | 2 (22.2%) | 13 (13.3%) |
| No | 9 (75.0%) | 9 (75.0%) | 8 (53.3%) | 6 (60.0%) | 3 (42.9%) | 15 (83.3%) | 9 (60.0%) | 7 (77.8%) | 66 (67.3%) |
| N/A | 2 (16.7%) | 3 (25.0%) | 4 (26.7%) | 4 (40.0%) | 2 (28.6%) | 2 (11.1%) | 2 (13.3%) | 0 (0.0%) | 19 (19.4%) |
| **Were confidence intervals included in the Kaplan-Meier curve?** | | | | | | | | | |
| No | 11 (91.7%) | 9 (75.0%) | 12 (80.0%) | 7 (70.0%) | 6 (85.7%) | 17 (94.4%) | 15 (100.0%) | 9 (100.0%) | 86 (87.8%) |
| N/A | 1 (8.3%) | 3 (25.0%) | 3 (20.0%) | 3 (30.0%) | 1 (14.3%) | 1 (5.6%) | 0 (0.0%) | 0 (0.0%) | 12 (12.2%) |
| **Primary analysis** |  |  |  |  |  |  |  |  |  |
| Hazard Ratio and 95% CI from an unadjusted Cox model | 0 (0.0%) | 0 (0.0%) | 0 (0.0%) | 0 (0.0%) | 0 (0.0%) | 0 (0.0%) | 1 (6.7%) | 0 (0.0%) | 1 (1.0%) |
| Hazard ratio and 95% CI from an adjusted Cox model | 0 (0.0%) | 0 (0.0%) | 0 (0.0%) | 1 (10.0%) | 0 (0.0%) | 1 (5.6%) | 0 (0.0%) | 0 (0.0%) | 2 (2.0%) |
| Hazard ratio and P-value from a logrank test | 4 (33.3%) | 3 (25.0%) | 0 (0.0%) | 2 (20.0%) | 1 (14.3%) | 3 (16.7%) | 3 (20.0%) | 3 (33.3%) | 19 (19.4%) |
| Hazard ratio and P-value from a stratified logrank test. | 1 (8.3%) | 0 (0.0%) | 1 (6.7%) | 1 (10.0%) | 0 (0.0%) | 1 (5.6%) | 0 (0.0%) | 2 (22.2%) | 6 (6.1%) |
| Hazard ratio and p-value from an adjusted Cox model | 2 (16.7%) | 1 (8.3%) | 4 (26.7%) | 2 (20.0%) | 1 (14.3%) | 2 (11.1%) | 3 (20.0%) | 0 (0.0%) | 15 (15.3%) |
| Hazard ratio and p-value from an unadjusted Cox model | 0 (0.0%) | 2 (16.7%) | 1 (6.7%) | 0 (0.0%) | 0 (0.0%) | 1 (5.6%) | 0 (0.0%) | 1 (11.1%) | 5 (5.1%) |
| Hazard ratio from a Cox model and p-value from a logrank test | 2 (16.7%) | 1 (8.3%) | 3 (20.0%) | 0 (0.0%) | 0 (0.0%) | 1 (5.6%) | 2 (13.3%) | 1 (11.1%) | 10 (10.2%) |
| Hazard ratio from an adjusted Cox model and p-value from a logrank test. | 1 (8.3%) | 0 (0.0%) | 0 (0.0%) | 1 (10.0%) | 0 (0.0%) | 1 (5.6%) | 2 (13.3%) | 1 (11.1%) | 6 (6.1%) |
| Hazard ratio from an adjusted Cox model and p-value from an stratified logrank test. | 1 (8.3%) | 2 (16.7%) | 3 (20.0%) | 0 (0.0%) | 2 (28.6%) | 2 (11.1%) | 2 (13.3%) | 1 (11.1%) | 13 (13.3%) |
| Hazard ratio from an unadjusted cox model and p-value from a logrank test. | 0 (0.0%) | 2 (16.7%) | 0 (0.0%) | 0 (0.0%) | 0 (0.0%) | 2 (11.1%) | 1 (6.7%) | 0 (0.0%) | 5 (5.1%) |
| Hazard ratio from an unadjusted cox model and p-value from an stratified logrank test. | 0 (0.0%) | 0 (0.0%) | 1 (6.7%) | 0 (0.0%) | 0 (0.0%) | 1 (5.6%) | 0 (0.0%) | 0 (0.0%) | 2 (2.0%) |
| N/A^1^ | 0 (0.0%) | 0 (0.0%) | 1 (6.7%) | 2 (20.0%) | 0 (0.0%) | 1 (5.6%) | 0 (0.0%) | 0 (0.0%) | 4 (4.1%) |
| Other | 1 (8.3%) | 1 (8.3%) | 1 (6.7%) | 1 (10.0%) | 3 (42.9%) | 2 (11.1%) | 1 (6.7%) | 0 (0.0%) | 10 (10.2%) |
| **Were subsequent treatments mentioned in the paper?** | | | | | | | | | |
| Yes | 10 (83.3%) | 6 (50.0%) | 8 (53.3%) | 4 (40.0%) | 4 (57.1%) | 14 (77.8%) | 9 (60.0%) | 4 (44.4%) | 59 (60.2%) |
| No | 2 (16.7%) | 6 (50.0%) | 7 (46.7%) | 6 (60.0%) | 3 (42.9%) | 4 (22.2%) | 6 (40.0%) | 5 (55.6%) | 39 (39.8%) |
| **Was the percentage or number of participants who received later lines included in the main text or appendix?** | | | | | | | | | |
| Yes | 9 (75.0%) | 4 (33.3%) | 6 (40.0%) | 2 (20.0%) | 2 (28.6%) | 14 (77.8%) | 9 (60.0%) | 4 (44.4%) | 50 (51.0%) |
| No | 1 (8.3%) | 2 (16.7%) | 1 (6.7%) | 2 (20.0%) | 2 (28.6%) | 0 (0.0%) | 0 (0.0%) | 0 (0.0%) | 8 (8.2%) |
| N/A | 2 (16.7%) | 6 (50.0%) | 8 (53.3%) | 6 (60.0%) | 3 (42.9%) | 4 (22.2%) | 6 (40.0%) | 5 (55.6%) | 40 (40.8%) |
| **Was a breakdown or summary of the number of subsequent treatments (Two, Three, Four) included in the main text or appendix?** | | | | | | | | | |
| Yes | 1 (8.3%) | 1 (8.3%) | 1 (6.7%) | 0 (0.0%) | 0 (0.0%) | 0 (0.0%) | 1 (6.7%) | 0 (0.0%) | 4 (4.1%) |
| No | 9 (75.0%) | 5 (41.7%) | 6 (40.0%) | 4 (40.0%) | 4 (57.1%) | 13 (72.2%) | 8 (53.3%) | 4 (44.4%) | 53 (54.1%) |
| N/A^2^ | 2 (16.7%) | 6 (50.0%) | 8 (53.3%) | 6 (60.0%) | 3 (42.9%) | 5 (27.8%) | 6 (40.0%) | 5 (55.6%) | 41 (41.8%) |
| **Was a breakdown or summary of the type of subsequent treatments (Treatment 1, Treatment 2, Treatment 3) included in the main text or appendix?** | | | | | | | | | |
| Yes | 8 (66.7%) | 4 (33.3%) | 5 (33.3%) | 1 (10.0%) | 2 (28.6%) | 13 (72.2%) | 8 (53.3%) | 4 (44.4%) | 45 (45.9%) |
| No | 2 (16.7%) | 2 (16.7%) | 2 (13.3%) | 3 (30.0%) | 2 (28.6%) | 0 (0.0%) | 1 (6.7%) | 0 (0.0%) | 12 (12.2%) |
| N/A^2^ | 2 (16.7%) | 6 (50.0%) | 8 (53.3%) | 6 (60.0%) | 3 (42.9%) | 5 (27.8%) | 6 (40.0%) | 5 (55.6%) | 41 (41.8%) |
| **Was additional analysis conducted to account for subsequent treatment lines?** | | | | | | | | | |
| Yes | 1 (8.3%) | 3 (25.0%) | 0 (0.0%) | 0 (0.0%) | 1 (14.3%) | 0 (0.0%) | 1 (6.7%) | 1 (11.1%) | 7 (7.1%) |
| No | 9 (75.0%) | 3 (25.0%) | 7 (46.7%) | 4 (40.0%) | 3 (42.9%) | 13 (72.2%) | 8 (53.3%) | 3 (33.3%) | 50 (51.0%) |
| N/A^2^ | 2 (16.7%) | 6 (50.0%) | 8 (53.3%) | 6 (60.0%) | 3 (42.9%) | 5 (27.8%) | 6 (40.0%) | 5 (55.6%) | 41 (41.8%) |
| **Were subsequent treatment lines mentioned in the discussion?** | | | | | | | | | |
| Yes | 7 (58.3%) | 4 (33.3%) | 5 (33.3%) | 3 (30.0%) | 3 (42.9%) | 8 (44.4%) | 6 (40.0%) | 3 (33.3%) | 39 (39.8%) |
| No | 3 (25.0%) | 2 (16.7%) | 3 (20.0%) | 1 (10.0%) | 1 (14.3%) | 6 (33.3%) | 3 (20.0%) | 1 (11.1%) | 20 (20.4%) |
| N/A | 2 (16.7%) | 6 (50.0%) | 7 (46.7%) | 6 (60.0%) | 3 (42.9%) | 4 (22.2%) | 6 (40.0%) | 5 (55.6%) | 39 (39.8%) |
| **How were subsequent treatments mentioned in the discussion?** | | | | | | | | | |
| The uptake of subsequent treatments is given as a reason for the discrepancy between OS and surrogate endpoints. | 1 (8.3%) | 1 (8.3%) | 0 (0.0%) | 0 (0.0%) | 0 (0.0%) | 0 (0.0%) | 0 (0.0%) | 0 (0.0%) | 2 (2.0%) |
| The uptake of subsequent treatments is given as a reason for better OS results or a reduced event rate than expected. | 0 (0.0%) | 0 (0.0%) | 2 (13.3%) | 0 (0.0%) | 1 (14.3%) | 1 (5.6%) | 1 (6.7%) | 1 (11.1%) | 6 (6.1%) |
| The uptake of subsequent treatments is given as a reason for reduced OS effect / stated as may have affected the results / used to caveat the results. | 3 (25.0%) | 1 (8.3%) | 0 (0.0%) | 1 (10.0%) | 2 (28.6%) | 4 (22.2%) | 2 (13.3%) | 1 (11.1%) | 14 (14.3%) |
| The lack of uptake of subsequent treatments is given as a reason for no OS effect / may have negatively affected OS. | 2 (16.7%) | 0 (0.0%) | 1 (6.7%) | 0 (0.0%) | 0 (0.0%) | 1 (5.6%) | 0 (0.0%) | 0 (0.0%) | 4 (4.1%) |
| Subsequent treatments are stated to not have affected the OS results as an OS benefit was observed or OS was similar between those who did and did not receive a subsequent treatment. | 0 (0.0%) | 1 (8.3%) | 0 (0.0%) | 0 (0.0%) | 0 (0.0%) | 1 (5.6%) | 1 (6.7%) | 0 (0.0%) | 3 (3.1%) |
| Randomisation / balance of subsequent treatment lines is given as a reason as to why subsequent treatment lines will not have affected the OS results. | 0 (0.0%) | 1 (8.3%) | 0 (0.0%) | 1 (10.0%) | 0 (0.0%) | 1 (5.6%) | 1 (6.7%) | 0 (0.0%) | 4 (4.1%) |
| None or limited options of subsequent treatment lines for patients are given as a reason as to why subsequent treatment lines will not have affected the OS results. | 0 (0.0%) | 0 (0.0%) | 2 (13.3%) | 0 (0.0%) | 0 (0.0%) | 0 (0.0%) | 0 (0.0%) | 0 (0.0%) | 2 (2.0%) |
| Other | 1 (8.3%) | 0 (0.0%) | 0 (0.0%) | 1 (10.0%) | 0 (0.0%) | 0 (0.0%) | 1 (6.7%) | 1 (11.1%) | 4 (4.1%) |
| N/A | 5 (41.7%) | 8 (66.7%) | 10 (66.7%) | 7 (70.0%) | 4 (57.1%) | 10 (55.6%) | 9 (60.0%) | 6 (66.7%) | 59 (60.2%) |

^1^ Not-Applicable here refers to papers where OS is a pre-specified endpoint, but the data is not mature at the time of the publication.

^2^ Note one paper stated no patients received subsequent treatments so is classed as N/A for these summaries.

N/A: Not Applicable; OS: Overall survival; GI: Gastrointestinal; HPB: Hepato-biliary

Supplementary Table 6: Summarised cost-effectiveness papers

| **ORN** | **Title** | **What analysis method was used for the Cost-Effectiveness analysis?** | **What OS estimate is used for the Cost-Effectiveness analysis?** | **Are the costs of subsequent treatment taken into account (in any form)** | **Was any additional analysis (e.g. RSPFTM, Two-stage etc.) presented to account for subsequent treatment lines or cross-over?** |
| --- | --- | --- | --- | --- | --- |
| 36 | The cost-effectiveness of atezolizumab in first-line for metastatic triple negative breast cancer is heavily linked to PD-L1 level. ^99^ | Unclear | HR | No Adjustment for subsequent costs | No |
| 49 | Cost-effectiveness analysis of pembrolizumab monotherapy versus chemotherapy for previously untreated advanced non-small cell lung cancer. ^100^ | Partitioned Survival Model | KM | Adjustment for subsequent costs | No |
| 53 | Cost-effectiveness of Pembrolizumab as Second-line Therapy for the Treatment of Locally Advanced or Metastatic Urothelial Carcinoma in Sweden. ^101^ | Partitioned Survival Model | KM | Adjustment for subsequent costs | Yes |
| 74 | Cost-effectiveness of pembrolizumab for advanced non-small cell lung cancer patients with varying comorbidity burden. ^102^ | Microsimulation | HR | Unclear | No |
| 84 | Cost effectiveness of pembrolizumab vs chemotherapy as first-line treatment for metastatic NSCLC that expresses high levels of PD-L1 in Switzerland. ^103^ | Partitioned Survival Model | KM | Adjustment for subsequent costs | Yes |
| 147 | Economic evaluation of rituximab in addition to standard of care chemotherapy for adult patients with acute lymphoblastic leukemia. ^104^ | Partitioned Survival Model | KM | Adjustment for subsequent costs | No |
| 173 | Cost-effectiveness of Pazopanib Versus Sunitinib as First-line Treatment for Locally Advanced or Metastatic Renal Cell Carcinoma from an Italian National Health Service Perspective. ^105^ | Partitioned Survival Model | KM | Adjustment for subsequent costs | No |
| 211 | Exploratory Cost-Effectiveness Analysis of Response-Guided Neoadjuvant Chemotherapy for Hormone Positive Breast Cancer Patients. ^106^ | Markov Model | HR | Unclear | Yes |
| 334 | Cost-effectiveness of cetuximab, cetuximab plus irinotecan, and panitumumab for third and further lines of treatment for KRAS wild-type patients with metastatic colorectal cancer. ^107^ | Partitioned Survival Model | Mean Survival | No Adjustment for subsequent costs | Yes |

Supplementary Table 7: Summarised Methodology / Review papers

| **ORN** | **Title** | **Year of Publication** | **Methodology / Review Category** | **If Mentioned - Summary** |
| --- | --- | --- | --- | --- |
| 8 | Two decades of research toward the treatment of locally advanced and metastatic pancreatic cancer: Remarkable effort and limited gain. ^108^ | 2021 | Treatment overview / Analysis - mention | First and second treatment lines and the evidence used to make them available are discussed. However, the impact of subsequent treatment lines on that evidence is not mentioned. |
| 14 | Sunitinib malate for gastrointestinal stromal tumour in imatinib mesylate-resistant patients: recommendations and evidence. ^109^ | 2010 | Treatment overview / Analysis - no mention | N/A |
| 32 | A comparison of partitioned survival analysis and state transition multi-state modelling approaches using a case study in oncology. ^110^ | 2020 | Methodology paper - mention | Only considered in the sense that subsequent costs have been considered in various models. |
| 35 | The Landmark Series: Locally Advanced Pancreatic Cancer and Ablative Therapy Options. [Review] ^111^ | 2021 | Treatment overview / Analysis - no mention | N/A |
| 42 | Hazard ratio of progression-free survival is an excellent predictor of overall survival in phase III randomized controlled trials evaluating the first-line chemotherapy for extensive-disease small-cell lung cancer. ^112^ | 2020 | Methodology paper - mention | It is mentioned in passing that sensitivity to first-line chemotherapy predict response to later-line chemotherapy and post-progression survival. They don't mention that the use of surrogate endpoints is motivated by the effect of subsequent lines. |
| 85 | Propensity Score Weighting Using Overlap Weights: A New Method Applied to Regorafenib Clinical Data and a Cost-Effectiveness Analysis. ^113^ | 2019 | Methodology paper - no mention | N/A |
| 115 | Pembrolizumab monotherapy versus chemotherapy for treatment of advanced urothelial carcinoma with disease progression during or following platinum-containing chemotherapy. A Cochrane Rapid Review. [Review] ^114^ | 2018 | Treatment overview / Analysis - no mention | N/A |
| 146 | Sample size determination for the current strategy in oncology Phase 3 trials that tests progression-free survival and overall survival in a two-stage design framework. ^115^ | 2018 | Methodology paper - mention | Subsequent lines are one of the motivations for the publication and said to be one of the reasons PFS is becoming commonly the primary endpoint and OS the secondary endpoint, but appropriate sample size calculations are required when they are Co-primary |
| 174 | Checkpoint inhibition: new treatment options in urologic cancer. [Review] ^116^ | 2017 | Treatment overview / Analysis - mention | Discussing the patient treatment pathway as a whole and where different types of treatment should fall. |
| 175 | First-line non-cytotoxic therapy in chemotherapy-naive patients with metastatic castration-resistant prostate cancer: a systematic review of 10 randomised clinical trials. [Review] ^117^ | 2017 | Treatment overview / Analysis - no mention | N/A |
| 185 | Cabozantinib in the treatment of advanced renal cell carcinoma in adults following prior vascular endothelial growth factor targeted therapy: clinical trial evidence and experience. [Review] ^118^ | 2018 | Treatment overview / Analysis - mention | Discussing the patient treatment pathway and where a certain drug should fall. However, the do mention that the availability of subsequent treatments may have affected the OS and PFS results they are considering. |
| 209 | Review of the Reporting of Survival Analyses within Randomised Controlled Trials and the Implications for Meta-Analysis. [Review] ^119^ | 2016 | Methodology paper - no mention | N/A |
| 217 | Evolution of Randomized Trials in Advanced/Metastatic Soft Tissue Sarcoma: End Point Selection, Surrogacy, and Quality of Reporting. [Review] ^120^ | 2016 | Methodology paper - mention | They use the issue of subsequent treatment lines as motivation for why they want to investigate surrogate endpoints. |
| 219 | First-Line Treatments for Poor-Prognosis Metastatic Renal Cell Carcinoma: Experts' Prescribing Practices and Systematic Literature Review. [Review] ^121^ | 2016 | Treatment overview / Analysis - no mention | N/A |
| 228 | Systemic therapy beyond first-line in advanced gastric cancer: An overview of the main randomized clinical trials. [Review] ^122^ | 2016 | Treatment overview / Analysis - no mention | N/A |
| 229 | Evaluating Intermittent Androgen-Deprivation Therapy Phase III Clinical Trials: The Devil Is in the Details. ^123^ | 2016 | Methodology paper - mention | Subsequent lines are mentioned in passing as affecting OS. |
| 232 | Why post-progression survival and post-relapse survival are not appropriate measures of efficacy in cancer randomized clinical trials. ^124^ | 2015 | Methodology paper - mention | The objective of the paper is to show that post-progression survival is not a way to account for subsequent treatments. Instead, causal methods should be used. The author suggests using a weighted cox-model to find the "per-protocol" effect and estimate what would have happened had everyone followed the protocol. |
| 239 | Systemic therapies for hepatocellular carcinoma. [Review] ^125^ | 2015 | Treatment overview / Analysis - no mention | N/A |
| 244 | Adaptive truncated weighting for improving marginal structural model estimation of treatment effects informally censored by subsequent therapy. ^126^ | 2015 | Methodology paper - mention | The objective of the paper is to compare different ways to account for subsequent lines and suggest a new way to calculate the weights in a marginal structural model. |
| 258 | MET inhibitors for treatment of advanced hepatocellular carcinoma: A review. [Review] ^127^ | 2015 | Treatment overview / Analysis - no mention | N/A |
| 274 | Intermediate clinical endpoints: a bridge between progression-free survival and overall survival in ovarian cancer trials. [Review] ^128^ | 2015 | Methodology paper - mention | The objective of the paper is to discuss the issues around subsequent treatment lines and whether PFS can be trusted. PFS2 and time to next therapy are suggested to be used alongside OS to provide a more comprehensive interpretation of the overall clinical benefit of an experimental treatment in a trial. |
| 276 | Progression-free survival as primary endpoint in randomized clinical trials of targeted agents for advanced renal cell carcinoma. Correlation with overall survival, benchmarking and power analysis. [Review] ^129^ | 2015 | Methodology paper - mention | The objective of the paper is to consider whether PFS can be classed as a surrogate endpoint for OS because of the potential confounding of subsequent lines of therapy. |
| 313 | Systematic review of surgical resection vs radiofrequency ablation for hepatocellular carcinoma. [Review] ^130^ | 2013 | Treatment overview / Analysis - no mention | N/A |
| 336 | Systematic chemotherapy for inoperable, locally advanced, recurrent, or metastatic uterine leiomyosarcoma: a systematic review. [Review] ^131^ | 2013 | Treatment overview / Analysis - no mention | N/A |
| 362 | Potential pitfalls of crossover and thoughts on iniparib in triple-negative breast cancer. [Review] ^132^ | 2011 | Methodology paper - mention | The paper discusses the impact of patients crossing over from the experimental to the control and control to the experimental and how this can impact the overall survival results. It's a good argument for why we need treatment switching methods. |
| 369 | Overall survival as the outcome for randomized clinical trials with effective subsequent therapies. [Review] ^133^ | 2011 | Methodology paper - mention | This talks about what the clinical benefit is in a number of scenarios. If the subsequent treatments are standard practice then the OS results even if non-sig show what would happen in the real world. |
| 397 | Current and future directions of clinical trials for ovarian cancer. [Review] ^134^ | 2011 | Treatment overview / Analysis - no mention | N/A |

# References

1. Kanemitsu Y, Shimizu Y, Mizusawa J, et al. Hepatectomy Followed by mFOLFOX6 Versus Hepatectomy Alone for Liver-Only Metastatic Colorectal Cancer (JCOG0603): A Phase II or III Randomized Controlled Trial. *J Clin Oncol* 2021; 39: 3789-3799. DOI: <https://dx.doi.org/10.1200/JCO.21.01032>.

2. Chopra S, Gupta S, Kannan S, et al. Late Toxicity After Adjuvant Conventional Radiation Versus Image-Guided Intensity-Modulated Radiotherapy for Cervical Cancer (PARCER): A Randomized Controlled Trial. *J Clin Oncol* 2021; 39: 3682-3692. DOI: <https://dx.doi.org/10.1200/JCO.20.02530>.

3. Reck M, Rodriguez-Abreu D, Robinson AG, et al. Five-Year Outcomes With Pembrolizumab Versus Chemotherapy for Metastatic Non-Small-Cell Lung Cancer With PD-L1 Tumor Proportion Score >= 50. *J Clin Oncol* 2021; 39: 2339-2349. Randomized Controlled Trial, Research Support, Non-U.S. Gov't. DOI: <https://dx.doi.org/10.1200/JCO.21.00174>.

4. Go SI, Lee SC, Bae WK, et al. Modified FOLFIRINOX versus S-1 as second-line chemotherapy in gemcitabine-failed metastatic pancreatic cancer patients: A randomised controlled trial (MPACA-3). *Eur J Cancer* 2021; 157: 21-30. Clinical Trial, Phase III, Comparative Study, Randomized Controlled Trial, Research Support, Non-U.S. Gov't. DOI: <https://dx.doi.org/10.1016/j.ejca.2021.08.002>.

5. Tang W, Ren L, Liu T, et al. Bevacizumab Plus mFOLFOX6 Versus mFOLFOX6 Alone as First-Line Treatment for RAS Mutant Unresectable Colorectal Liver-Limited Metastases: The BECOME Randomized Controlled Trial. *J Clin Oncol* 2020; 38: 3175-3184. Comparative Study, Randomized Controlled Trial, Research Support, Non-U.S. Gov't. DOI: <https://dx.doi.org/10.1200/JCO.20.00174>.

6. Shi Y, Lei K, Jia Y, et al. Bevacizumab biosimilar LY01008 compared with bevacizumab (Avastin) as first-line treatment for Chinese patients with unresectable, metastatic, or recurrent non-squamous non-small-cell lung cancer: A multicenter, randomized, double-blinded, phase III trial. *Cancer Commun (Lond)* 2021; 41: 889-903. Clinical Trial, Phase III, Multicenter Study, Randomized Controlled Trial, Research Support, Non-U.S. Gov't. DOI: <https://dx.doi.org/10.1002/cac2.12179>.

7. Murthy V, Maitre P, Kannan S, et al. Prostate-Only Versus Whole-Pelvic Radiation Therapy in High-Risk and Very High-Risk Prostate Cancer (POP-RT): Outcomes From Phase III Randomized Controlled Trial. *J Clin Oncol* 2021; 39: 1234-1242. Clinical Trial, Phase III, Randomized Controlled Trial, Research Support, Non-U.S. Gov't. DOI: <https://dx.doi.org/10.1200/JCO.20.03282>.

8. Lamarca A, Palmer DH, Wasan HS, et al. Second-line FOLFOX chemotherapy versus active symptom control for advanced biliary tract cancer (ABC-06): a phase 3, open-label, randomised, controlled trial. *Lancet Oncol* 2021; 22: 690-701. Clinical Trial, Phase III, Comparative Study, Randomized Controlled Trial, Research Support, Non-U.S. Gov't. DOI: <https://dx.doi.org/10.1016/S1470-2045(21)00027-9>.

9. Lin NU, Borges V, Anders C, et al. Intracranial Efficacy and Survival With Tucatinib Plus Trastuzumab and Capecitabine for Previously Treated HER2-Positive Breast Cancer With Brain Metastases in the HER2CLIMB Trial. *J Clin Oncol* 2020; 38: 2610-2619. Randomized Controlled Trial, Research Support, Non-U.S. Gov't. DOI: <https://dx.doi.org/10.1200/JCO.20.00775>.

10. Tomita Y, Naito S, Sassa N, et al. Sunitinib Versus Sorafenib as Initial Targeted Therapy for mCC-RCC With Favorable/Intermediate Risk: Multicenter Randomized Trial CROSS-J-RCC. *Clin Genitourin Cancer* 2020; 18: e374-e385. Clinical Trial, Phase III, Comparative Study, Multicenter Study, Randomized Controlled Trial, Research Support, Non-U.S. Gov't. DOI: <https://dx.doi.org/10.1016/j.clgc.2020.01.001>.

11. Sezer A, Kilickap S, Gumus M, et al. Cemiplimab monotherapy for first-line treatment of advanced non-small-cell lung cancer with PD-L1 of at least 50%: a multicentre, open-label, global, phase 3, randomised, controlled trial. *Lancet* 2021; 397: 592-604. Clinical Trial, Phase III, Multicenter Study, Randomized Controlled Trial, Research Support, Non-U.S. Gov't. DOI: <https://dx.doi.org/10.1016/S0140-6736(21)00228-2>.

12. Geng R, Wang G, Qiu L, et al. Metronomic capecitabine as maintenance treatment after first line induction with XELOX for metastatic colorectal cancer patients. *Medicine (Baltimore)* 2020; 99: e23719. Randomized Controlled Trial. DOI: <https://dx.doi.org/10.1097/MD.0000000000023719>.

13. Kumar SK, Jacobus SJ, Cohen AD, et al. Carfilzomib or bortezomib in combination with lenalidomide and dexamethasone for patients with newly diagnosed multiple myeloma without intention for immediate autologous stem-cell transplantation (ENDURANCE): a multicentre, open-label, phase 3, randomised, controlled trial. *The Lancet Oncology* 2020; 21: 1317-1330. DOI: 10.1016/s1470-2045(20)30452-6.

14. Malone S, Roy S, Eapen L, et al. Sequencing of Androgen-Deprivation Therapy With External-Beam Radiotherapy in Localized Prostate Cancer: A Phase III Randomized Controlled Trial. *J Clin Oncol* 2020; 38: 593-601. Clinical Trial, Phase III, Randomized Controlled Trial, Research Support, Non-U.S. Gov't. DOI: <https://dx.doi.org/10.1200/JCO.19.01904>.

15. Mazzaferro V, Citterio D, Bhoori S, et al. Liver transplantation in hepatocellular carcinoma after tumour downstaging (XXL): a randomised, controlled, phase 2b/3 trial. *Lancet Oncol* 2020; 21: 947-956. Clinical Trial, Phase II, Clinical Trial, Phase III, Multicenter Study, Randomized Controlled Trial, Research Support, Non-U.S. Gov't. DOI: <https://dx.doi.org/10.1016/S1470-2045(20)30224-2>.

16. Clamp AR, James EC, McNeish IA, et al. Weekly dose-dense chemotherapy in first-line epithelial ovarian, fallopian tube, or primary peritoneal carcinoma treatment (ICON8): primary progression free survival analysis results from a GCIG phase 3 randomised controlled trial. *Lancet* 2019; 394: 2084-2095. Clinical Trial, Phase III, Randomized Controlled Trial, Research Support, Non-U.S. Gov't. DOI: <https://dx.doi.org/10.1016/S0140-6736(19)32259-7>.

17. Hong YS, Kim SY, Lee JS, et al. Oxaliplatin-Based Adjuvant Chemotherapy for Rectal Cancer After Preoperative Chemoradiotherapy (ADORE): Long-Term Results of a Randomized Controlled Trial. *J Clin Oncol* 2019; 37: 3111-3123. Clinical Trial, Phase II, Multicenter Study, Randomized Controlled Trial, Research Support, Non-U.S. Gov't. DOI: <https://dx.doi.org/10.1200/JCO.19.00016>.

18. Minnaar CA, Kotzen JA, Ayeni OA, et al. The effect of modulated electro-hyperthermia on local disease control in HIV-positive and -negative cervical cancer women in South Africa: Early results from a phase III randomised controlled trial. *PLoS ONE* 2019; 14: e0217894. Clinical Trial, Phase III, Randomized Controlled Trial, Research Support, Non-U.S. Gov't. DOI: <https://dx.doi.org/10.1371/journal.pone.0217894>.

19. Xu L, Qi Q, Zhang Y, et al. Combination of icotinib and chemotherapy as first-line treatment for advanced lung adenocarcinoma in patients with sensitive EGFR mutations: A randomized controlled study. *Lung Cancer* 2019; 133: 23-31. Multicenter Study, Randomized Controlled Trial. DOI: <https://dx.doi.org/10.1016/j.lungcan.2019.05.008>.

20. Zhong WZ, Chen KN, Chen C, et al. Erlotinib Versus Gemcitabine Plus Cisplatin as Neoadjuvant Treatment of Stage IIIA-N2 EGFR-Mutant Non-Small-Cell Lung Cancer (EMERGING-CTONG 1103): A Randomized Phase II Study. *J Clin Oncol* 2019; 37: 2235-2245. Clinical Trial, Phase II, Multicenter Study, Randomized Controlled Trial, Research Support, Non-U.S. Gov't. DOI: <https://dx.doi.org/10.1200/JCO.19.00075>.

21. Rini BI, Powles T, Atkins MB, et al. Atezolizumab plus bevacizumab versus sunitinib in patients with previously untreated metastatic renal cell carcinoma (IMmotion151): a multicentre, open-label, phase 3, randomised controlled trial. *Lancet* 2019; 393: 2404-2415. Clinical Trial, Phase III, Comparative Study, Multicenter Study, Randomized Controlled Trial, Research Support, Non-U.S. Gov't. DOI: <https://dx.doi.org/10.1016/S0140-6736(19)30723-8>.

22. Eng C, Kim TW, Bendell J, et al. Atezolizumab with or without cobimetinib versus regorafenib in previously treated metastatic colorectal cancer (IMblaze370): a multicentre, open-label, phase 3, randomised, controlled trial. *Lancet Oncol* 2019; 20: 849-861. Clinical Trial, Phase III, Multicenter Study, Randomized Controlled Trial, Research Support, Non-U.S. Gov't. DOI: <https://dx.doi.org/10.1016/S1470-2045(19)30027-0>.

23. MacManus M, Fisher R, Roos D, et al. Randomized Trial of Systemic Therapy After Involved-Field Radiotherapy in Patients With Early-Stage Follicular Lymphoma: TROG 99.03. *J Clin Oncol* 2018; 36: 2918-2925. Clinical Trial, Phase III, Multicenter Study, Randomized Controlled Trial, Research Support, Non-U.S. Gov't. DOI: <https://dx.doi.org/10.1200/JCO.2018.77.9892>.

24. Engel-Riedel W, Lowe J, Mattson P, et al. A randomized, controlled trial evaluating the efficacy and safety of BTH1677 in combination with bevacizumab, carboplatin, and paclitaxel in first-line treatment of advanced non-small cell lung cancer. *J Immunother Cancer* 2018; 6: 16. Clinical Trial, Phase II, Multicenter Study, Randomized Controlled Trial, Research Support, Non-U.S. Gov't. DOI: <https://dx.doi.org/10.1186/s40425-018-0324-z>.

25. Gupta S, Maheshwari A, Parab P, et al. Neoadjuvant Chemotherapy Followed by Radical Surgery Versus Concomitant Chemotherapy and Radiotherapy in Patients With Stage IB2, IIA, or IIB Squamous Cervical Cancer: A Randomized Controlled Trial. *J Clin Oncol* 2018; 36: 1548-1555. Clinical Trial, Phase III, Comparative Study, Randomized Controlled Trial, Research Support, Non-U.S. Gov't. DOI: <https://dx.doi.org/10.1200/JCO.2017.75.9985>.

26. Aparicio T, Ghiringhelli F, Boige V, et al. Bevacizumab Maintenance Versus No Maintenance During Chemotherapy-Free Intervals in Metastatic Colorectal Cancer: A Randomized Phase III Trial (PRODIGE 9). *J Clin Oncol* 2018; 36: 674-681. Clinical Trial, Phase III, Comparative Study, Multicenter Study, Randomized Controlled Trial, Research Support, Non-U.S. Gov't. DOI: <https://dx.doi.org/10.1200/JCO.2017.75.2931>.

27. Powles T, Duran I, van der Heijden MS, et al. Atezolizumab versus chemotherapy in patients with platinum-treated locally advanced or metastatic urothelial carcinoma (IMvigor211): a multicentre, open-label, phase 3 randomised controlled trial. *Lancet* 2018; 391: 748-757. Clinical Trial, Phase III, Comparative Study, Multicenter Study, Randomized Controlled Trial Research Support, Non-U.S. Gov't. DOI: <https://dx.doi.org/10.1016/S0140-6736(17)33297-X>.

28. Mason MD, Clarke NW, James ND, et al. Adding Celecoxib With or Without Zoledronic Acid for Hormone-Naive Prostate Cancer: Long-Term Survival Results From an Adaptive, Multiarm, Multistage, Platform, Randomized Controlled Trial. *J Clin Oncol* 2017; 35: 1530-1541. Randomized Controlled Trial. DOI: <https://dx.doi.org/10.1200/JCO.2016.69.0677>.

29. Nokihara H, Lu S, Mok TSK, et al. Randomized controlled trial of S-1 versus docetaxel in patients with non-small-cell lung cancer previously treated with platinum-based chemotherapy (East Asia S-1 Trial in Lung Cancer). *Ann Oncol* 2017; 28: 2698-2706. Clinical Trial, Phase III, Multicenter Study, Randomized Controlled Trial. DOI: <https://dx.doi.org/10.1093/annonc/mdx419>.

30. Cunningham D, Stenning SP, Smyth EC, et al. Peri-operative chemotherapy with or without bevacizumab in operable oesophagogastric adenocarcinoma (UK Medical Research Council ST03): primary analysis results of a multicentre, open-label, randomised phase 2-3 trial. *Lancet Oncol* 2017; 18: 357-370. Clinical Trial, Phase II, Clinical Trial, Phase III, Comparative Study, Multicenter Study, Randomized Controlled Trial, Research Support, Non-U.S. Gov't. DOI: <https://dx.doi.org/10.1016/S1470-2045(17)30043-8>.

31. Joshi A, Patil V, Noronha V, et al. Results of a phase II randomized controlled clinical trial comparing efficacy of Cabazitaxel versus Docetaxel as second line or above therapy in recurrent head and neck cancer. *Oral Oncol* 2017; 75: 54-60. Clinical Trial, Phase II, Comparative Study, Randomized Controlled Trial. DOI: <https://dx.doi.org/10.1016/j.oraloncology.2017.10.018>.

32. Miles D, Im YH, Fung A, et al. Effect of docetaxel duration on clinical outcomes: exploratory analysis of CLEOPATRA, a phase III randomized controlled trial. *Ann Oncol* 2017; 28: 2761-2767. Clinical Trial, Phase III, Randomized Controlled Trial, Research Support, Non-U.S. Gov't. DOI: <https://dx.doi.org/10.1093/annonc/mdx406>.

33. Dimopoulos MA, Goldschmidt H, Niesvizky R, et al. Carfilzomib or bortezomib in relapsed or refractory multiple myeloma (ENDEAVOR): an interim overall survival analysis of an open-label, randomised, phase 3 trial. *Lancet Oncol* 2017; 18: 1327-1337. Clinical Trial, Phase III, Comparative Study, Multicenter Study, Randomized Controlled Trial, Research Support, Non-U.S. Gov't. DOI: <https://dx.doi.org/10.1016/S1470-2045(17)30578-8>.

34. Pignata S, Scambia G, Bologna A, et al. Randomized Controlled Trial Testing the Efficacy of Platinum-Free Interval Prolongation in Advanced Ovarian Cancer: The MITO-8, MaNGO, BGOG-Ov1, AGO-Ovar2.16, ENGOT-Ov1, GCIG Study. *J Clin Oncol* 2017; 35: 3347-3353. Clinical Trial, Phase III, Multicenter Study, Randomized Controlled Trial. DOI: <https://dx.doi.org/10.1200/JCO.2017.73.4293>.

35. Rittmeyer A, Barlesi F, Waterkamp D, et al. Atezolizumab versus docetaxel in patients with previously treated non-small-cell lung cancer (OAK): a phase 3, open-label, multicentre randomised controlled trial. *The Lancet* 2017; 389: 255-265. DOI: 10.1016/s0140-6736(16)32517-x.

36. Belani CP, Chakraborty BC, Modi RI, et al. A randomized trial of TLR-2 agonist CADI-05 targeting desmocollin-3 for advanced non-small-cell lung cancer. *Ann Oncol* 2017; 28: 298-304. Multicenter Study, Randomized Controlled Trial, Research Support, Non-U.S. Gov't. DOI: <https://dx.doi.org/10.1093/annonc/mdw608>.

37. Cheng Y, Fan Y, Liu X, et al. Randomized controlled trial of lobaplatin plus etoposide vs. cisplatin plus etoposide as first-line therapy in patients with extensive-stage small cell lung cancer. *Oncol* 2019; 17: 4701-4709. DOI: <https://dx.doi.org/10.3892/ol.2019.10125>.

38. Zhu Y, Zhang W, Li Q, et al. A Phase II Randomized Controlled Trial: Definitive Concurrent Chemoradiotherapy with Docetaxel Plus Cisplatin versus 5-Fluorouracil plus Cisplatin in Patients with Oesophageal Squamous Cell Carcinoma. *J* 2017; 8: 3657-3666. DOI: <https://dx.doi.org/10.7150/jca.20053>.

39. James ND, Sydes MR, Clarke NW, et al. Addition of docetaxel, zoledronic acid, or both to first-line long-term hormone therapy in prostate cancer (STAMPEDE): survival results from an adaptive, multiarm, multistage, platform randomised controlled trial. *Lancet* 2016; 387: 1163-1177. Multicenter Study, Randomized Controlled Trial, Research Support, Non-U.S. Gov't. DOI: <https://dx.doi.org/10.1016/S0140-6736(15)01037-5>.

40. Hodi FS, Chesney J, Pavlick AC, et al. Combined nivolumab and ipilimumab versus ipilimumab alone in patients with advanced melanoma: 2-year overall survival outcomes in a multicentre, randomised, controlled, phase 2 trial. *Lancet Oncol* 2016; 17: 1558-1568. Clinical Trial, Phase II, Multicenter Study, Randomized Controlled Trial. DOI: <https://dx.doi.org/10.1016/S1470-2045(16)30366-7>.

41. Hasegawa K, Saiura A, Takayama T, et al. Adjuvant Oral Uracil-Tegafur with Leucovorin for Colorectal Cancer Liver Metastases: A Randomized Controlled Trial. *PLoS ONE* 2016; 11: e0162400. Clinical Trial, Phase III, Multicenter Study, Randomized Controlled Trial. DOI: <https://dx.doi.org/10.1371/journal.pone.0162400>.

42. James N, Pirrie S, Pope A, et al. TRAPEZE: a randomised controlled trial of the clinical effectiveness and cost-effectiveness of chemotherapy with zoledronic acid, strontium-89, or both, in men with bony metastatic castration-refractory prostate cancer. *Health Technol Assess* 2016; 20: 1-288. Clinical Trial, Phase II, Clinical Trial, Phase III, Randomized Controlled Trial. DOI: <https://dx.doi.org/10.3310/hta20530>.

43. Carrie C, Hasbini A, de Laroche G, et al. Salvage radiotherapy with or without short-term hormone therapy for rising prostate-specific antigen concentration after radical prostatectomy (GETUG-AFU 16): a randomised, multicentre, open-label phase 3 trial. *Lancet Oncol* 2016; 17: 747-756. Clinical Trial, Phase III, Comparative Study, Multicenter Study, Randomized Controlled Trial. DOI: <https://dx.doi.org/10.1016/S1470-2045(16)00111-X>.

44. Park K, Tan EH, O'Byrne K, et al. Afatinib versus gefitinib as first-line treatment of patients with EGFR mutation-positive non-small-cell lung cancer (LUX-Lung 7): a phase 2B, open-label, randomised controlled trial. *Lancet Oncol* 2016; 17: 577-589. Clinical Trial, Phase II, Randomized Controlled Trial. DOI: <https://dx.doi.org/10.1016/S1470-2045(16)30033-X>.

45. Fehrenbacher L, Spira A, Ballinger M, et al. Atezolizumab versus docetaxel for patients with previously treated non-small-cell lung cancer (POPLAR): a multicentre, open-label, phase 2 randomised controlled trial. *Lancet* 2016; 387: 1837-1846. Clinical Trial, Phase II, Multicenter Study, Randomized Controlled Trial, Research Support, Non-U.S. Gov't. DOI: <https://dx.doi.org/10.1016/S0140-6736(16)00587-0>.

46. Fujitani K, Yang HK, Mizusawa J, et al. Gastrectomy plus chemotherapy versus chemotherapy alone for advanced gastric cancer with a single non-curable factor (REGATTA): a phase 3, randomised controlled trial. *Lancet Oncol* 2016; 17: 309-318. Clinical Trial, Phase III, Comparative Study, Multicenter Study, Randomized Controlled Trial, Research Support, Non-U.S. Gov't. DOI: <https://dx.doi.org/10.1016/S1470-2045(15)00553-7>.

47. Herbst RS, Baas P, Kim DW, et al. Pembrolizumab versus docetaxel for previously treated, PD-L1-positive, advanced non-small-cell lung cancer (KEYNOTE-010): a randomised controlled trial. *Lancet* 2016; 387: 1540-1550. Multicenter Study, Randomized Controlled Trial, Research Support, Non-U.S. Gov't. DOI: <https://dx.doi.org/10.1016/S0140-6736(15)01281-7>.

48. Oza AM, Cook AD, Pfisterer J, et al. Standard chemotherapy with or without bevacizumab for women with newly diagnosed ovarian cancer (ICON7): overall survival results of a phase 3 randomised trial. *Lancet Oncol* 2015; 16: 928-936. Clinical Trial, Phase III, Multicenter Study, Randomized Controlled Trial, Research Support, Non-U.S. Gov't. DOI: <https://dx.doi.org/10.1016/S1470-2045(15)00086-8>.

49. Bielack SS, Smeland S, Whelan JS, et al. Methotrexate, Doxorubicin, and Cisplatin (MAP) Plus Maintenance Pegylated Interferon Alfa-2b Versus MAP Alone in Patients With Resectable High-Grade Osteosarcoma and Good Histologic Response to Preoperative MAP: First Results of the EURAMOS-1 Good Response Randomized Controlled Trial. *J Clin Oncol* 2015; 33: 2279-2287. Clinical Trial, Phase III, Comparative Study, Multicenter Study, Randomized Controlled Trial, Research Support, Non-U.S. Gov't, Webcast. DOI: <https://dx.doi.org/10.1200/JCO.2014.60.0734>.

50. Kehoe S, Hook J, Nankivell M, et al. Primary chemotherapy versus primary surgery for newly diagnosed advanced ovarian cancer (CHORUS): an open-label, randomised, controlled, non-inferiority trial. *Lancet* 2015; 386: 249-257. Clinical Trial, Phase III, Comparative Study, Multicenter Study, Randomized Controlled Trial, Research Support, Non-U.S. Gov't. DOI: <https://dx.doi.org/10.1016/S0140-6736(14)62223-6>.

51. Mason MD, Parulekar WR, Sydes MR, et al. Final Report of the Intergroup Randomized Study of Combined Androgen-Deprivation Therapy Plus Radiotherapy Versus Androgen-Deprivation Therapy Alone in Locally Advanced Prostate Cancer. *J Clin Oncol* 2015; 33: 2143-2150. Randomized Controlled Trial, Research Support, N.I.H., Extramural, Research Support, Non-U.S. Gov't. DOI: <https://dx.doi.org/10.1200/JCO.2014.57.7510>.

52. Badwe R, Hawaldar R, Nair N, et al. Locoregional treatment versus no treatment of the primary tumour in metastatic breast cancer: an open-label randomised controlled trial. *Lancet Oncol* 2015; 16: 1380-1388. Comparative Study, Randomized Controlled Trial, Research Support, Non-U.S. Gov't. DOI: <https://dx.doi.org/10.1016/S1470-2045(15)00135-7>.

53. Bear HD, Tang G, Rastogi P, et al. Neoadjuvant plus adjuvant bevacizumab in early breast cancer (NSABP B-40 [NRG Oncology]): secondary outcomes of a phase 3, randomised controlled trial. *Lancet Oncol* 2015; 16: 1037-1048. Clinical Trial, Phase III, Randomized Controlled Trial, Research Support, N.I.H., Extramural, Research Support, Non-U.S. Gov't. DOI: <https://dx.doi.org/10.1016/S1470-2045(15)00041-8>.

54. Shapiro J, van Lanschot JJB, Hulshof M, et al. Neoadjuvant chemoradiotherapy plus surgery versus surgery alone for oesophageal or junctional cancer (CROSS): long-term results of a randomised controlled trial. *Lancet Oncol* 2015; 16: 1090-1098. Randomized Controlled Trial, Research Support, Non-U.S. Gov't. DOI: <https://dx.doi.org/10.1016/S1470-2045(15)00040-6>.

55. Henderson MA, Burmeister BH, Ainslie J, et al. Adjuvant lymph-node field radiotherapy versus observation only in patients with melanoma at high risk of further lymph-node field relapse after lymphadenectomy (ANZMTG 01.02/TROG 02.01): 6-year follow-up of a phase 3, randomised controlled trial. *Lancet Oncol* 2015; 16: 1049-1060. Clinical Trial, Phase III, Randomized Controlled Trial, Research Support, Non-U.S. Gov't. DOI: <https://dx.doi.org/10.1016/S1470-2045(15)00187-4>.

56. Soria JC, Felip E, Cobo M, et al. Afatinib versus erlotinib as second-line treatment of patients with advanced squamous cell carcinoma of the lung (LUX-Lung 8): an open-label randomised controlled phase 3 trial. *Lancet Oncol* 2015; 16: 897-907. Clinical Trial, Phase III, Comparative Study, Multicenter Study, Randomized Controlled Trial, Research Support, Non-U.S. Gov't. DOI: <https://dx.doi.org/10.1016/S1470-2045(15)00006-6>.

57. Zapatero A, Guerrero A, Maldonado X, et al. High-dose radiotherapy with short-term or long-term androgen deprivation in localised prostate cancer (DART01/05 GICOR): a randomised, controlled, phase 3 trial. *Lancet Oncol* 2015; 16: 320-327. Clinical Trial, Phase III, Multicenter Study, Randomized Controlled Trial, Research Support, Non-U.S. Gov't. DOI: <https://dx.doi.org/10.1016/S1470-2045(15)70045-8>.

58. Kunkler IH, Williams LJ, Jack WJ, et al. Breast-conserving surgery with or without irradiation in women aged 65 years or older with early breast cancer (PRIME II): a randomised controlled trial. *Lancet Oncol* 2015; 16: 266-273. Clinical Trial, Phase III, Multicenter Study, Randomized Controlled Trial, Research Support, Non-U.S. Gov't. DOI: <https://dx.doi.org/10.1016/S1470-2045(14)71221-5>.

59. Bartelink H, Maingon P, Poortmans P, et al. Whole-breast irradiation with or without a boost for patients treated with breast-conserving surgery for early breast cancer: 20-year follow-up of a randomised phase 3 trial. *Lancet Oncol* 2015; 16: 47-56. Clinical Trial, Phase III, Multicenter Study, Randomized Controlled Trial, Research Support, Non-U.S. Gov't. DOI: <https://dx.doi.org/10.1016/S1470-2045(14)71156-8>.

60. Slotman BJ, van Tinteren H, Praag JO, et al. Use of thoracic radiotherapy for extensive stage small-cell lung cancer: a phase 3 randomised controlled trial. *Lancet* 2015; 385: 36-42. Clinical Trial, Phase III, Multicenter Study, Randomized Controlled Trial, Research Support, Non-U.S. Gov't. DOI: <https://dx.doi.org/10.1016/S0140-6736(14)61085-0>.

61. Middleton G, Silcocks P, Cox T, et al. Gemcitabine and capecitabine with or without telomerase peptide vaccine GV1001 in patients with locally advanced or metastatic pancreatic cancer (TeloVac): an open-label, randomised, phase 3 trial. *Lancet Oncol* 2014; 15: 829-840. Clinical Trial, Phase III, Multicenter Study, Randomized Controlled Trial, Research Support, Non-U.S. Gov't. DOI: <https://dx.doi.org/10.1016/S1470-2045(14)70236-0>.

62. Vora A, Goulden N, Mitchell C, et al. Augmented post-remission therapy for a minimal residual disease-defined high-risk subgroup of children and young people with clinical standard-risk and intermediate-risk acute lymphoblastic leukaemia (UKALL 2003): a randomised controlled trial. *Lancet Oncol* 2014; 15: 809-818. Randomized Controlled Trial, Research Support, Non-U.S. Gov't. DOI: <https://dx.doi.org/10.1016/S1470-2045(14)70243-8>.

63. Corrie PG, Marshall A, Dunn JA, et al. Adjuvant bevacizumab in patients with melanoma at high risk of recurrence (AVAST-M): preplanned interim results from a multicentre, open-label, randomised controlled phase 3 study. *Lancet Oncol* 2014; 15: 620-630. Clinical Trial, Phase III, Multicenter Study, Randomized Controlled Trial, Research Support, Non-U.S. Gov't. DOI: <https://dx.doi.org/10.1016/S1470-2045(14)70110-X>.

64. Dearnaley DP, Jovic G, Syndikus I, et al. Escalated-dose versus control-dose conformal radiotherapy for prostate cancer: long-term results from the MRC RT01 randomised controlled trial. *Lancet Oncol* 2014; 15: 464-473. Clinical Trial, Phase III, Comparative Study, Multicenter Study, Randomized Controlled Trial, Research Support, Non-U.S. Gov't. DOI: <https://dx.doi.org/10.1016/S1470-2045(14)70040-3>.

65. Judson I, Verweij J, Gelderblom H, et al. Doxorubicin alone versus intensified doxorubicin plus ifosfamide for first-line treatment of advanced or metastatic soft-tissue sarcoma: a randomised controlled phase 3 trial. *Lancet Oncol* 2014; 15: 415-423. Clinical Trial, Phase III, Comparative Study, Randomized Controlled Trial, Research Support, Non-U.S. Gov't. DOI: <https://dx.doi.org/10.1016/S1470-2045(14)70063-4>.

66. Zuliani AC, Esteves SC, Teixeira LC, et al. Concomitant cisplatin plus radiotherapy and high-dose-rate brachytherapy versus radiotherapy alone for stage IIIB epidermoid cervical cancer: a randomized controlled trial. *J Clin Oncol* 2014; 32: 542-547. Randomized Controlled Trial. DOI: <https://dx.doi.org/10.1200/JCO.2013.50.1205>.

67. Reck M, Kaiser R, Mellemgaard A, et al. Docetaxel plus nintedanib versus docetaxel plus placebo in patients with previously treated non-small-cell lung cancer (LUME-Lung 1): a phase 3, double-blind, randomised controlled trial. *Lancet Oncol* 2014; 15: 143-155. Clinical Trial, Phase III, Comparative Study, Multicenter Study, Randomized Controlled Trial, Research Support, Non-U.S. Gov't. DOI: <https://dx.doi.org/10.1016/S1470-2045(13)70586-2>.

68. Ford HE, Marshall A, Bridgewater JA, et al. Docetaxel versus active symptom control for refractory oesophagogastric adenocarcinoma (COUGAR-02): an open-label, phase 3 randomised controlled trial. *Lancet Oncol* 2014; 15: 78-86. Clinical Trial, Phase III, Comparative Study, Multicenter Study, Randomized Controlled Trial, Research Support, Non-U.S. Gov't. DOI: <https://dx.doi.org/10.1016/S1470-2045(13)70549-7>.

69. Poplin E, Wasan H, Rolfe L, et al. Randomized, multicenter, phase II study of CO-101 versus gemcitabine in patients with metastatic pancreatic ductal adenocarcinoma: including a prospective evaluation of the role of hENT1 in gemcitabine or CO-101 sensitivity. *J Clin Oncol* 2013; 31: 4453-4461. Clinical Trial, Phase II, Multicenter Study, Randomized Controlled Trial, Research Support, Non-U.S. Gov't. DOI: <https://dx.doi.org/10.1200/JCO.2013.51.0826>.

70. Goldhirsch A, Gelber RD, Piccart-Gebhart MJ, et al. 2 years versus 1 year of adjuvant trastuzumab for HER2-positive breast cancer (HERA): an open-label, randomised controlled trial. *Lancet* 2013; 382: 1021-1028. Clinical Trial, Phase III, Comparative Study, Multicenter Study, Randomized Controlled Trial, Research Support, Non-U.S. Gov't. DOI: <https://dx.doi.org/10.1016/S0140-6736(13)61094-6>.

71. Yamamoto Y, Ishikawa T, Hozumi Y, et al. Randomized controlled trial of toremifene 120 mg compared with exemestane 25 mg after prior treatment with a non-steroidal aromatase inhibitor in postmenopausal women with hormone receptor-positive metastatic breast cancer. *BMC Cancer* 2013; 13: 239. Comparative Study, Randomized Controlled Trial. DOI: <https://dx.doi.org/10.1186/1471-2407-13-239>.

72. Crosby T, Hurt CN, Falk S, et al. Chemoradiotherapy with or without cetuximab in patients with oesophageal cancer (SCOPE1): a multicentre, phase 2/3 randomised trial. *Lancet Oncol* 2013; 14: 627-637. Clinical Trial, Phase II, Clinical Trial, Phase III, Comparative Study, Multicenter Study, Randomized Controlled Trial, Research Support, Non-U.S. Gov't. DOI: <https://dx.doi.org/10.1016/S1470-2045(13)70136-0>.

73. Veronesi U, Orecchia R, Maisonneuve P, et al. Intraoperative radiotherapy versus external radiotherapy for early breast cancer (ELIOT): a randomised controlled equivalence trial. *Lancet Oncol* 2013; 14: 1269-1277. Randomized Controlled Trial, Research Support, Non-U.S. Gov't. DOI: <https://dx.doi.org/10.1016/S1470-2045(13)70497-2>.

74. Katsumata N, Yasuda M, Isonishi S, et al. Long-term results of dose-dense paclitaxel and carboplatin versus conventional paclitaxel and carboplatin for treatment of advanced epithelial ovarian, fallopian tube, or primary peritoneal cancer (JGOG 3016): a randomised, controlled, open-label trial. *Lancet Oncol* 2013; 14: 1020-1026. Randomized Controlled Trial, Research Support, Non-U.S. Gov't. DOI: <https://dx.doi.org/10.1016/S1470-2045(13)70363-2>.

75. Garassino MC, Martelli O, Broggini M, et al. Erlotinib versus docetaxel as second-line treatment of patients with advanced non-small-cell lung cancer and wild-type EGFR tumours (TAILOR): a randomised controlled trial. *Lancet Oncol* 2013; 14: 981-988. Comparative Study, Randomized Controlled Trial, Research Support, Non-U.S. Gov't. DOI: <https://dx.doi.org/10.1016/S1470-2045(13)70310-3>.

76. Ye LC, Liu TS, Ren L, et al. Randomized controlled trial of cetuximab plus chemotherapy for patients with KRAS wild-type unresectable colorectal liver-limited metastases. *J Clin Oncol* 2013; 31: 1931-1938. Randomized Controlled Trial, Research Support, Non-U.S. Gov't. DOI: <https://dx.doi.org/10.1200/JCO.2012.44.8308>.

77. Penichoux J, Michiels S, Bouche O, et al. Taking into account successive treatment lines in the analysis of a colorectal cancer randomised trial. *Eur J Cancer* 2013; 49: 1882-1888. Research Support, Non-U.S. Gov't. DOI: <https://dx.doi.org/10.1016/j.ejca.2013.02.006>.

78. Peng ZW, Zhang YJ, Chen MS, et al. Radiofrequency ablation with or without transcatheter arterial chemoembolization in the treatment of hepatocellular carcinoma: a prospective randomized trial. *J Clin Oncol* 2013; 31: 426-432. Randomized Controlled Trial, Research Support, Non-U.S. Gov't. DOI: <https://dx.doi.org/10.1200/JCO.2012.42.9936>.

79. Flotten O, Gronberg BH, Bremnes R, et al. Vinorelbine and gemcitabine vs vinorelbine and carboplatin as first-line treatment of advanced NSCLC. A phase III randomised controlled trial by the Norwegian Lung Cancer Study Group. *Br J Cancer* 2012; 107: 442-447. Clinical Trial, Phase III, Multicenter Study, Randomized Controlled Trial. DOI: <https://dx.doi.org/10.1038/bjc.2012.284>.

80. James ND, Sydes MR, Mason MD, et al. Celecoxib plus hormone therapy versus hormone therapy alone for hormone-sensitive prostate cancer: first results from the STAMPEDE multiarm, multistage, randomised controlled trial. *Lancet Oncol* 2012; 13: 549-558. Clinical Trial, Phase II, Clinical Trial, Phase III, Multicenter Study, Randomized Controlled Trial, Research Support, Non-U.S. Gov't. DOI: <https://dx.doi.org/10.1016/S1470-2045(12)70088-8>.

81. de Gramont A, Van Cutsem E, Schmoll HJ, et al. Bevacizumab plus oxaliplatin-based chemotherapy as adjuvant treatment for colon cancer (AVANT): a phase 3 randomised controlled trial. *Lancet Oncol* 2012; 13: 1225-1233. Clinical Trial, Phase III, Multicenter Study, Randomized Controlled Trial, Research Support, Non-U.S. Gov't. DOI: <https://dx.doi.org/10.1016/S1470-2045(12)70509-0>.

82. Woll PJ, Reichardt P, Le Cesne A, et al. Adjuvant chemotherapy with doxorubicin, ifosfamide, and lenograstim for resected soft-tissue sarcoma (EORTC 62931): a multicentre randomised controlled trial. *Lancet Oncol* 2012; 13: 1045-1054. Multicenter Study, Randomized Controlled Trial, Research Support, N.I.H., Extramural, Research Support, Non-U.S. Gov't. DOI: <https://dx.doi.org/10.1016/S1470-2045(12)70346-7>.

83. Burmeister BH, Henderson MA, Ainslie J, et al. Adjuvant radiotherapy versus observation alone for patients at risk of lymph-node field relapse after therapeutic lymphadenectomy for melanoma: a randomised trial. *Lancet Oncol* 2012; 13: 589-597. Multicenter Study, Randomized Controlled Trial, Research Support, Non-U.S. Gov't. DOI: <https://dx.doi.org/10.1016/S1470-2045(12)70138-9>.

84. Bang YJ, Kim YW, Yang HK, et al. Adjuvant capecitabine and oxaliplatin for gastric cancer after D2 gastrectomy (CLASSIC): a phase 3 open-label, randomised controlled trial. *Lancet* 2012; 379: 315-321. Clinical Trial, Phase III, Multicenter Study, Randomized Controlled Trial, Research Support, Non-U.S. Gov't. DOI: <https://dx.doi.org/10.1016/S0140-6736(11)61873-4>.

85. Adams RA, Meade AM, Seymour MT, et al. Intermittent versus continuous oxaliplatin and fluoropyrimidine combination chemotherapy for first-line treatment of advanced colorectal cancer: results of the randomised phase 3 MRC COIN trial. *Lancet Oncol* 2011; 12: 642-653. Clinical Trial, Clinical Trial, Phase III, Multicenter Study, Randomized Controlled Trial, Research Support, Non-U.S. Gov't. DOI: <https://dx.doi.org/10.1016/S1470-2045(11)70102-4>.

86. Maughan TS, Adams RA, Smith CG, et al. Addition of cetuximab to oxaliplatin-based first-line combination chemotherapy for treatment of advanced colorectal cancer: results of the randomised phase 3 MRC COIN trial. *Lancet* 2011; 377: 2103-2114. Clinical Trial, Phase III, Multicenter Study, Randomized Controlled Trial, Research Support, Non-U.S. Gov't. DOI: <https://dx.doi.org/10.1016/S0140-6736(11)60613-2>.

87. Morgan GJ, Davies FE, Gregory WM, et al. First-line treatment with zoledronic acid as compared with clodronic acid in multiple myeloma (MRC Myeloma IX): a randomised controlled trial. *Lancet* 2010; 376: 1989-1999. Comparative Study, Multicenter Study Randomized Controlled Trial, Research Support, Non-U.S. Gov't. DOI: <https://dx.doi.org/10.1016/S0140-6736(10)62051-X>.

88. Cuzick J, Sestak I, Baum M, et al. Effect of anastrozole and tamoxifen as adjuvant treatment for early-stage breast cancer: 10-year analysis of the ATAC trial. *Lancet Oncol* 2010; 11: 1135-1141. Clinical Trial, Randomized Controlled Trial, Research Support, Non-U.S. Gov't. DOI: <https://dx.doi.org/10.1016/S1470-2045(10)70257-6>.

89. Rustin GJ, van der Burg ME, Griffin CL, et al. Early versus delayed treatment of relapsed ovarian cancer (MRC OV05/EORTC 55955): a randomised trial. *Lancet* 2010; 376: 1155-1163. Comparative Study, Multicenter Study, Randomized Controlled Trial, Research Support, Non-U.S. Gov't. DOI: <https://dx.doi.org/10.1016/S0140-6736(10)61268-8>.

90. Issels RD, Lindner LH, Verweij J, et al. Neo-adjuvant chemotherapy alone or with regional hyperthermia for localised high-risk soft-tissue sarcoma: a randomised phase 3 multicentre study. *Lancet Oncol* 2010; 11: 561-570. Clinical Trial, Phase III, Multicenter Study, Randomized Controlled Trial, Research Support, N.I.H., Extramural Research Support, Non-U.S. Gov't. DOI: <https://dx.doi.org/10.1016/S1470-2045(10)70071-1>.

91. Zietman AL, Bae K, Slater JD, et al. Randomized trial comparing conventional-dose with high-dose conformal radiation therapy in early-stage adenocarcinoma of the prostate: long-term results from proton radiation oncology group/american college of radiology 95-09. *J Clin Oncol* 2010; 28: 1106-1111. Clinical Trial, Phase I, Clinical Trial, Phase II, Comparative Study, Randomized Controlled Trial, Research Support, N.I.H., Extramural. DOI: <https://dx.doi.org/10.1200/JCO.2009.25.8475>.

92. Kantoff PW, Schuetz TJ, Blumenstein BA, et al. Overall survival analysis of a phase II randomized controlled trial of a Poxviral-based PSA-targeted immunotherapy in metastatic castration-resistant prostate cancer. *J Clin Oncol* 2010; 28: 1099-1105. Clinical Trial, Phase II, Multicenter Study, Randomized Controlled Trial, Research Support, N.I.H., Extramural. DOI: <https://dx.doi.org/10.1200/JCO.2009.25.0597>.

93. Rajkumar SV, Jacobus S, Callander NS, et al. Lenalidomide plus high-dose dexamethasone versus lenalidomide plus low-dose dexamethasone as initial therapy for newly diagnosed multiple myeloma: an open-label randomised controlled trial. *Lancet Oncol* 2010; 11: 29-37. Comparative Study, Multicenter Study, Randomized Controlled Trial, Research Support, N.I.H., Extramural Research Support, U.S. Gov't, Non-P.H.S. DOI: <https://dx.doi.org/10.1016/S1470-2045(09)70284-0>.

94. Sutton L, Chevret S, Tournilhac O, et al. Autologous stem cell transplantation as a first-line treatment strategy for chronic lymphocytic leukemia: a multicenter, randomized, controlled trial from the SFGM-TC and GFLLC. *Blood* 2011; 117: 6109-6119. Multicenter Study, Randomized Controlled Trial, Research Support, Non-U.S. Gov't. DOI: <https://dx.doi.org/10.1182/blood-2010-11-317073>.

95. Badwe R, Hawaldar R, Parmar V, et al. Single-injection depot progesterone before surgery and survival in women with operable breast cancer: a randomized controlled trial. *J Clin Oncol* 2011; 29: 2845-2851. Randomized Controlled Trial, Research Support, Non-U.S. Gov't. DOI: <https://dx.doi.org/10.1200/JCO.2010.33.0738>.

96. Gianni L, Dafni U, Gelber RD, et al. Treatment with trastuzumab for 1 year after adjuvant chemotherapy in patients with HER2-positive early breast cancer: a 4-year follow-up of a randomised controlled trial. *Lancet Oncol* 2011; 12: 236-244. Clinical Trial, Phase III, Randomized Controlled Trial, Research Support, Non-U.S. Gov't. DOI: <https://dx.doi.org/10.1016/S1470-2045(11)70033-X>.

97. Sharma A, Dwary AD, Mohanti BK, et al. Best supportive care compared with chemotherapy for unresectable gall bladder cancer: a randomized controlled study. *J Clin Oncol* 2010; 28: 4581-4586. Comparative Study, Randomized Controlled Trial. DOI: <https://dx.doi.org/10.1200/JCO.2010.29.3605>.

98. Bang YJ, Van Cutsem E, Feyereislova A, et al. Trastuzumab in combination with chemotherapy versus chemotherapy alone for treatment of HER2-positive advanced gastric or gastro-oesophageal junction cancer (ToGA): a phase 3, open-label, randomised controlled trial. *Lancet* 2010; 376: 687-697. Clinical Trial, Phase III, Comparative Study, Multicenter Study, Randomized Controlled Trial, Research Support, Non-U.S. Gov't. DOI: <https://dx.doi.org/10.1016/S0140-6736(10)61121-X>.

99. Giuliani J, Mantoan B and Bonetti A. The cost-effectiveness of atezolizumab in first-line for metastatic triple negative breast cancer is heavily linked to PD-L1 level. *J Oncol Pharm Pract* 2021; 27: 1245-1247. DOI: <https://dx.doi.org/10.1177/10781552211019462>.

100. Aziz MIA, Tan LE, Tan WHG, et al. Cost-effectiveness analysis of pembrolizumab monotherapy versus chemotherapy for previously untreated advanced non-small cell lung cancer. *J Med Econ* 2020; 23: 952-960. Randomized Controlled Trial. DOI: <https://dx.doi.org/10.1080/13696998.2020.1775620>.

101. Srivastava T, Prabhu VS, Li H, et al. Cost-effectiveness of Pembrolizumab as Second-line Therapy for the Treatment of Locally Advanced or Metastatic Urothelial Carcinoma in Sweden. *Eur Urol Oncol* 2020; 3: 663-670. Research Support, Non-U.S. Gov't. DOI: <https://dx.doi.org/10.1016/j.euo.2018.09.012>.

102. Criss SD, Palazzo L, Watson TR, et al. Cost-effectiveness of pembrolizumab for advanced non-small cell lung cancer patients with varying comorbidity burden. *PLoS ONE* 2020; 15: e0228288. Research Support, N.I.H., Extramural. DOI: <https://dx.doi.org/10.1371/journal.pone.0228288>.

103. Bhadhuri A, Insinga R, Guggisberg P, et al. Cost effectiveness of pembrolizumab vs chemotherapy as first-line treatment for metastatic NSCLC that expresses high levels of PD-L1 in Switzerland. *Swiss Med Wkly* 2019; 149: w20170. Comparative Study. DOI: <https://dx.doi.org/10.4414/smw.2019.20170>.

104. Nam J, Milenkovski R, Yunger S, et al. Economic evaluation of rituximab in addition to standard of care chemotherapy for adult patients with acute lymphoblastic leukemia. *J Med Econ* 2018; 21: 47-59. Evaluation Study. DOI: <https://dx.doi.org/10.1080/13696998.2017.1372230>.

105. Capri S, Porta C and Delea TE. Cost-effectiveness of Pazopanib Versus Sunitinib as First-line Treatment for Locally Advanced or Metastatic Renal Cell Carcinoma from an Italian National Health Service Perspective. *Clin Ther* 2017; 39: 567-580.e562. Comparative Study. DOI: <https://dx.doi.org/10.1016/j.clinthera.2017.01.017>.

106. Miquel-Cases A, Retel VP, Lederer B, et al. Exploratory Cost-Effectiveness Analysis of Response-Guided Neoadjuvant Chemotherapy for Hormone Positive Breast Cancer Patients. *PLoS ONE* 2016; 11: e0154386. Randomized Controlled Trial, Research Support, Non-U.S. Gov't. DOI: <https://dx.doi.org/10.1371/journal.pone.0154386>.

107. Hoyle M, Peters J, Crathorne L, et al. Cost-effectiveness of cetuximab, cetuximab plus irinotecan, and panitumumab for third and further lines of treatment for KRAS wild-type patients with metastatic colorectal cancer. *Value Health* 2013; 16: 288-296. Comparative Study, Research Support, Non-U.S. Gov't. DOI: <https://dx.doi.org/10.1016/j.jval.2012.11.001>.

108. Yeh C and Bates SE. Two decades of research toward the treatment of locally advanced and metastatic pancreatic cancer: Remarkable effort and limited gain. *Semin Oncol* 2021; 48: 34-46. DOI: <https://dx.doi.org/10.1053/j.seminoncol.2021.01.001>.

109. Younus J, Verma S, Franek J, et al. Sunitinib malate for gastrointestinal stromal tumour in imatinib mesylate-resistant patients: recommendations and evidence. *Curr* 2010; 17: 4-10.

110. Cranmer H, Shields GE and Bullement A. A comparison of partitioned survival analysis and state transition multi-state modelling approaches using a case study in oncology. *J Med Econ* 2020; 23: 1176-1185. DOI: <https://dx.doi.org/10.1080/13696998.2020.1796360>.

111. White RR, Murphy JD and Martin RCG. The Landmark Series: Locally Advanced Pancreatic Cancer and Ablative Therapy Options. *Ann Surg Oncol* 2021; 28: 4173-4180. Review. DOI: <https://dx.doi.org/10.1245/s10434-021-09662-z>.

112. Chen H, Horita N, Ito K, et al. Hazard ratio of progression-free survival is an excellent predictor of overall survival in phase III randomized controlled trials evaluating the first-line chemotherapy for extensive-disease small-cell lung cancer. *Transl* 2020; 9: 1333-1342. DOI: <https://dx.doi.org/10.21037/tlcr-20-377>.

113. Mlcoch T, Hrnciarova T, Tuzil J, et al. Propensity Score Weighting Using Overlap Weights: A New Method Applied to Regorafenib Clinical Data and a Cost-Effectiveness Analysis. *Value Health* 2019; 22: 1370-1377. Evaluation Study, Research Support, Non-U.S. Gov't. DOI: <https://dx.doi.org/10.1016/j.jval.2019.06.010>.

114. Narayan V, Kahlmeyer A, Dahm P, et al. Pembrolizumab monotherapy versus chemotherapy for treatment of advanced urothelial carcinoma with disease progression during or following platinum-containing chemotherapy. A Cochrane Rapid Review. *Cochrane Database Syst Rev* 2018; 7: CD012838. Research Support, Non-U.S. Gov't, Review, Systematic Review. DOI: <https://dx.doi.org/10.1002/14651858.CD012838.pub2>.

115. Nomura S, Hirakawa A and Hamada C. Sample size determination for the current strategy in oncology Phase 3 trials that tests progression-free survival and overall survival in a two-stage design framework. *J Biopharm Stat* 2018; 28: 589-611. Research Support, Non-U.S. Gov't. DOI: <https://dx.doi.org/10.1080/10543406.2017.1372775>.

116. De Maeseneer DJ, Delafontaine B and Rottey S. Checkpoint inhibition: new treatment options in urologic cancer. *Acta Clin Belg* 2017; 72: 24-28. Review. DOI: <https://dx.doi.org/10.1080/17843286.2016.1260890>.

117. Poorthuis MHF, Vernooij RWM, van Moorselaar RJA, et al. First-line non-cytotoxic therapy in chemotherapy-naive patients with metastatic castration-resistant prostate cancer: a systematic review of 10 randomised clinical trials. *BJU Int* 2017; 119: 831-845. Review, Systematic Review. DOI: <https://dx.doi.org/10.1111/bju.13764>.

118. Osanto S and van der Hulle T. Cabozantinib in the treatment of advanced renal cell carcinoma in adults following prior vascular endothelial growth factor targeted therapy: clinical trial evidence and experience. *Ther Adv Urol* 2018; 10: 109-123. Review. DOI: <https://dx.doi.org/10.1177/1756287217748867>.

119. Batson S, Greenall G and Hudson P. Review of the Reporting of Survival Analyses within Randomised Controlled Trials and the Implications for Meta-Analysis. *PLoS ONE* 2016; 11: e0154870. Review, Research Support, Non-U.S. Gov't. DOI: <https://dx.doi.org/10.1371/journal.pone.0154870>.

120. Zer A, Prince RM, Amir E, et al. Evolution of Randomized Trials in Advanced/Metastatic Soft Tissue Sarcoma: End Point Selection, Surrogacy, and Quality of Reporting. *J Clin Oncol* 2016; 34: 1469-1475. Review, Systematic Review. DOI: <https://dx.doi.org/10.1200/JCO.2015.64.3437>.

121. Le Saux O, Freyer G and Negrier S. First-Line Treatments for Poor-Prognosis Metastatic Renal Cell Carcinoma: Experts' Prescribing Practices and Systematic Literature Review. *Clin Drug Invest* 2016; 36: 389-399. Review, Systematic Review. DOI: <https://dx.doi.org/10.1007/s40261-016-0384-0>.

122. Galdy S, Cella CA, Spada F, et al. Systemic therapy beyond first-line in advanced gastric cancer: An overview of the main randomized clinical trials. *Crit Rev Oncol Hematol* 2016; 99: 1-12. Research Support, Non-U.S. Gov't, Review. DOI: <https://dx.doi.org/10.1016/j.critrevonc.2015.09.004>.

123. Hussain M, Tangen C, Higano C, et al. Evaluating Intermittent Androgen-Deprivation Therapy Phase III Clinical Trials: The Devil Is in the Details. *J Clin Oncol* 2016; 34: 280-285. Clinical Trial, Phase III, Randomized Controlled Trial. DOI: <https://dx.doi.org/10.1200/JCO.2015.62.8065>.

124. Garcia-Albeniz X, Maurel J and Hernan MA. Why post-progression survival and post-relapse survival are not appropriate measures of efficacy in cancer randomized clinical trials. *Int J Cancer* 2015; 136: 2444-2447. Research Support, N.I.H., Extramural. DOI: <https://dx.doi.org/10.1002/ijc.29278>.

125. Ge S and Huang D. Systemic therapies for hepatocellular carcinoma. *Drug discov* 2015; 9: 352-362. Review. DOI: <https://dx.doi.org/10.5582/ddt.2015.01047>.

126. Bai X, Liu J, Li L, et al. Adaptive truncated weighting for improving marginal structural model estimation of treatment effects informally censored by subsequent therapy. *Pharm* 2015; 14: 448-454. DOI: <https://dx.doi.org/10.1002/pst.1719>.

127. Qi XS, Guo XZ, Han GH, et al. MET inhibitors for treatment of advanced hepatocellular carcinoma: A review. *World J Gastroenterol* 2015; 21: 5445-5453. Review. DOI: <https://dx.doi.org/10.3748/wjg.v21.i18.5445>.

128. Matulonis UA, Oza AM, Ho TW, et al. Intermediate clinical endpoints: a bridge between progression-free survival and overall survival in ovarian cancer trials. *Cancer* 2015; 121: 1737-1746. Research Support, Non-U.S. Gov't, Review. DOI: <https://dx.doi.org/10.1002/cncr.29082>.

129. Bria E, Massari F, Maines F, et al. Progression-free survival as primary endpoint in randomized clinical trials of targeted agents for advanced renal cell carcinoma. Correlation with overall survival, benchmarking and power analysis. *Crit Rev Oncol Hematol* 2015; 93: 50-59. Research Support, Non-U.S. Gov't, Review. DOI: <https://dx.doi.org/10.1016/j.critrevonc.2014.08.001>.

130. Cucchetti A, Piscaglia F, Cescon M, et al. Systematic review of surgical resection vs radiofrequency ablation for hepatocellular carcinoma. *World J Gastroenterol* 2013; 19: 4106-4118. Research Support, Non-U.S. Gov't, Review, Systematic Review. DOI: <https://dx.doi.org/10.3748/wjg.v19.i26.4106>.

131. Gupta AA, Yao X, Verma S, et al. Systematic chemotherapy for inoperable, locally advanced, recurrent, or metastatic uterine leiomyosarcoma: a systematic review. *Clin Oncol (R Coll Radiol)* 2013; 25: 346-355. Research Support, Non-U.S. Gov't, Review, Systematic Review. DOI: <https://dx.doi.org/10.1016/j.clon.2012.11.008>.

132. Fojo T, Amiri-Kordestani L and Bates SE. Potential pitfalls of crossover and thoughts on iniparib in triple-negative breast cancer. *J Natl Cancer Inst* 2011; 103: 1738-1740. Research Support, N.I.H., Intramural, Review. DOI: <https://dx.doi.org/10.1093/jnci/djr386>.

133. Korn EL, Freidlin B and Abrams JS. Overall survival as the outcome for randomized clinical trials with effective subsequent therapies. *J Clin Oncol* 2011; 29: 2439-2442. Review. DOI: <https://dx.doi.org/10.1200/JCO.2011.34.6056>.

134. Gardner GJ and Jewell EL. Current and future directions of clinical trials for ovarian cancer. *Cancer Control* 2011; 18: 44-51. Review.
